# Supplementary material for: Systematic review of changed smoking behaviour, smoking cessation and psychological states of smokers according to cigarette type during the COVID-19 pandemic
Source: BMJ Open. 2022 Jun 14;12(6):e055179. doi: 10.1136/bmjopen-2021-055179 (PMC9198387; doi:10.1136/bmjopen-2021-055179)
Supplement: Supplementary data [file bmjopen-2021-055179supp001.pdf]

Supplemantary

Online Appendix : Search terms

Table 1 Pubmed search terms

Electronic Nicotine Delivery Systems
(((((((((((((((((((((((((((((((((((((((Electronic Nicotine Delivery System[Title/Abstract]) OR (Electronic Cigarettes[Title/Abstract])) OR (E-Cigs[Title/Abstract])) OR (E Cigs[Title/Abstract])) OR (E-Cig[Title/Abstract])) OR (E Cig[Title/Abstract])) OR (E-Cigarettes[Title/Abstract])) OR (E Cigarettes[Title/Abstract])) OR (E-Cigarette[Title/Abstract])) OR (E Cigarette[Title/Abstract])) OR (Electronic Cigarette[Title/Abstract])) OR (Cigarette, Electronic[Title/Abstract])) OR (Cigarettes, Electronic[Title/Abstract])) OR ("Electronic Nicotine Delivery Systems"[Mesh])) OR ("Vaping"[Mesh])) OR (E-Cig Use[Title/Abstract])) OR (E Cig Use[Title/Abstract])) OR (E-Cig Uses[Title/Abstract])) OR (Use, E-Cig[Title/Abstract])) OR (ECig Use[Title/Abstract])) OR (ECig Uses[Title/Abstract])) OR (Use, ECig[Title/Abstract])) OR (Vape[Title/Abstract])) OR (Vapes[Title/Abstract])) OR (E-Cigarette Use[Title/Abstract])) OR (E Cigarette Use[Title/Abstract])) OR (E-Cigarette Uses[Title/Abstract])) OR (Use, E-Cigarette[Title/Abstract])) OR (Nicotine Vaping[Title/Abstract])) OR (Nicotine Vapings[Title/Abstract])) OR (Vaping, Nicotine[Title/Abstract])) OR (Vapings, Nicotine[Title/Abstract])) OR (Ecigarette Use[Title/Abstract])) OR (Ecigarette Uses[Title/Abstract])) OR (Use, Ecigarette[Title/Abstract])) OR (Uses, Ecigarette[Title/Abstract])) OR (Electronic Cigarette Use[Title/Abstract])) OR (Cigarette Use, Electronic[Title/Abstract])) OR (Electronic Cigarette Uses[Title/Abstract])) OR (Use, Electronic Cigarette[Title/Abstract])
COVID-19
(((((((((((((((((((((((((((((((((((COVID 19[Title/Abstract]) OR (COVID-19 Virus Disease[Title/Abstract])) OR (COVID 19 Virus Disease[Title/Abstract])) OR (COVID-19 Virus Diseases[Title/Abstract])) OR (Disease, COVID-19 Virus[Title/Abstract])) OR (Virus Disease, COVID-19[Title/Abstract])) OR (COVID-19 Virus Infection[Title/Abstract])) OR (COVID 19 Virus Infection[Title/Abstract])) OR (COVID-19 Virus Infections[Title/Abstract])) OR (Infection, COVID-19 Virus[Title/Abstract])) OR (Virus Infection, COVID-19[Title/Abstract])) OR (2019-nCoV Infection[Title/Abstract])) OR (2019 nCoV Infection[Title/Abstract])) OR (2019-nCoV Infections[Title/Abstract])) OR (Infection, 2019-nCoV[Title/Abstract])) OR (Coronavirus Disease-19[Title/Abstract])) OR (Coronavirus Disease 19[Title/Abstract])) OR (2019 Novel Coronavirus Disease[Title/Abstract])) OR (2019 Novel Coronavirus Infection[Title/Abstract])) OR (2019-nCoV Disease[Title/Abstract])) OR (2019 nCoV Disease[Title/Abstract])) OR (2019-nCoV

Diseases[Title/Abstract])) OR (Disease, 2019-nCoV[Title/Abstract])) OR (COVID19[Title/Abstract])) OR (Coronavirus Disease 2019[Title/Abstract])) OR (Disease 2019, Coronavirus[Title/Abstract])) OR (SARS Coronavirus 2 Infection[Title/Abstract])) OR (Infection, SARS-CoV-2[Title/Abstract])) OR (SARS-CoV-2 Infection[Title/Abstract])) OR (SARS CoV 2 Infection[Title/Abstract])) OR (SARS-CoV-2 Infections[Title/Abstract])) OR (COVID-19 Pandemic[Title/Abstract])) OR (COVID 19 Pandemic[Title/Abstract])) OR (COVID-19 Pandemics[Title/Abstract])) OR (Pandemic, COVID-19[Title/Abstract])) OR ("COVID-19"[Mesh])
Tobacco, Tobacco Products, Tobacco Use
(((((Tobacco Uses[Title/Abstract]) OR (Tobacco Consumption[Title/Abstract])) OR (Consumption, Tobacco[Title/Abstract])) OR ("Tobacco Use"[Mesh])) OR ((((((((((((((((Tobaccos[Title/Abstract]) OR (Nicotiana[Title/Abstract])) OR (Nicotianas[Title/Abstract])) OR (Nicotiana tabacum[Title/Abstract])) OR (Nicotiana tabacums[Title/Abstract])) OR (tabacum, Nicotiana[Title/Abstract])) OR ("Tobacco"[Mesh])) OR ("Smoking"[Mesh])) OR (Smoking Behaviors[Title/Abstract])) OR (Behavior, Smoking[Title/Abstract])) OR (Behaviors, Smoking[Title/Abstract])) OR (Smoking Behavior[Title/Abstract])) OR (Smoking Habit[Title/Abstract])) OR (Habit, Smoking[Title/Abstract])) OR (Habits, Smoking[Title/Abstract])) OR (Smoking Habits[Title/Abstract]))) OR (((((((((((((((((((((((((Product, Tobacco[Title/Abstract]) OR (Products, Tobacco[Title/Abstract])) OR (Tobacco Product[Title/Abstract])) OR (Cigarillos[Title/Abstract])) OR (Cigarillo[Title/Abstract])) OR (Pipe Tobacco[Title/Abstract])) OR (Pipe Tobaccos[Title/Abstract])) OR (Tobacco, Pipe[Title/Abstract])) OR (Cigars[Title/Abstract])) OR (Tobaccos, Pipe[Title/Abstract])) OR (Cigar[Title/Abstract])) OR (Kreteks[Title/Abstract])) OR (Kretek[Title/Abstract])) OR (Kreteks Tobacco[Title/Abstract])) OR (Kreteks Tobaccos[Title/Abstract])) OR (Tobacco, Kreteks[Title/Abstract])) OR (Tobaccos, Kreteks[Title/Abstract])) OR (Bidis[Title/Abstract])) OR (Bidi[Title/Abstract])) OR (Bidis Tobacco[Title/Abstract])) OR (Bidis Tobaccos[Title/Abstract])) OR (Tobacco, Bidis[Title/Abstract])) OR (Cigarettes[Title/Abstract])) OR (Cigarette[Title/Abstract])) OR ("Tobacco Products"[Mesh]))
Psychology
(("Psychology"[Mesh]) OR ("Psychology/diagnosis"[Mesh] OR "Psychology/psychology"[Mesh])) OR ((((((((((((((Side Effects, Psychological[Title/Abstract]) OR (Psychological Side Effect[Title/Abstract])) OR (Side Effect, Psychological[Title/Abstract])) OR (Psychological Side Effects[Title/Abstract])) OR (Psychosocial Factors[Title/Abstract])) OR (Factor, Psychosocial[Title/Abstract])) OR (Factors, Psychosocial[Title/Abstract])) OR (Psychosocial Factor[Title/Abstract])) OR (Psychological Factors[Title/Abstract])) OR

(Factor, Psychological[Title/Abstract])) OR (Psychological Factor[Title/Abstract])) OR (Factors, Psychological[Title/Abstract])) OR (Psychologist[Title/Abstract])) OR (Psychologists[Title/Abstract]))
Anxiety
((((((((Angst[Title/Abstract]) OR (Nervousness[Title/Abstract])) OR (Hypervigilance[Title/Abstract])) OR (Anxiousness[Title/Abstract])) OR (Social Anxiety[Title/Abstract])) OR (Anxieties, Social[Title/Abstract])) OR (Anxiety, Social[Title/Abstract])) OR (Social Anxieties[Title/Abstract])) OR (("Anxiety"[Mesh]) OR ("Anxiety/analysis"[Mesh] OR "Anxiety/diagnosis"[Mesh] OR "Anxiety/psychology"[Mesh]))
Stress, Psychological
(((((((((((((Psychological Stresses[Title/Abstract]) OR (Stresses, Psychological[Title/Abstract])) OR (Life Stress[Title/Abstract])) OR (Life Stresses[Title/Abstract])) OR (Stress, Life[Title/Abstract])) OR (Stresses, Life[Title/Abstract])) OR (Stress, Psychologic[Title/Abstract])) OR (Psychologic Stress[Title/Abstract])) OR (Stressor, Psychological[Title/Abstract])) OR (Psychological Stressor[Title/Abstract])) OR (Psychological Stressors[Title/Abstract])) OR (Stressors, Psychological[Title/Abstract])) OR (Psychological Stress[Title/Abstract])) OR (("Stress, Psychological"[Mesh]) OR ("Stress, Psychological/analysis"[Mesh] OR "Stress, Psychological/diagnosis"[Mesh] OR "Stress, Psychological/psychology"[Mesh]))
Depression
(("Depression"[Mesh]) OR ("Depression/analysis"[Mesh] OR "Depression/diagnosis"[Mesh] OR "Depression/psychology"[Mesh])) OR (((((((((Depressions[Title/Abstract]) OR (Depressive Symptoms[Title/Abstract])) OR (Depressive Symptom[Title/Abstract])) OR (Symptom, Depressive[Title/Abstract])) OR (Symptoms, Depressive[Title/Abstract])) OR (Emotional Depression[Title/Abstract])) OR (Depression, Emotional[Title/Abstract])) OR (Depressions, Emotional[Title/Abstract])) OR (Emotional Depressions[Title/Abstract]))

Table 2 Science Direct search terms

Query
COVID AND (e-cigarette OR "electronic cigarette" OR "electronic nicotine delivery" OR "vaping" OR "heat not burn" OR "heated tobacco product")
COVID AND (tobaccos OR cigarette OR kretek OR bidis OR "pipe tobacco" OR cigarillos)
