# Supplementary material for: Systematic review of changed smoking behaviour, smoking cessation and psychological states of smokers according to cigarette type during the COVID-19 pandemic
Source: BMJ Open. 2022 Jun 14;12(6):e055179. doi: 10.1136/bmjopen-2021-055179 (PMC9198387; doi:10.1136/bmjopen-2021-055179)
Supplement: Author's manuscript [file bmjopen-2021-055179.draft_revisions.pdf]

BMJ Open is committed to open peer review. As part of this commitment we make the peer review history of every article we publish publicly available.

When an article is published we post the peer reviewers' comments and the authors' responses online. We also post the versions of the paper that were used during peer review. These are the versions that the peer review comments apply to.

The versions of the paper that follow are the versions that were submitted during the peer review process. They are not the versions of record or the final published versions. They should not be cited or distributed as the published version of this manuscript.

BMJ Open is an open access journal and the full, final, typeset and author-corrected version of record of the manuscript is available on our site with no access controls, subscription charges or pay-per-view fees (http://bmjopen.bmj.com).

If you have any questions on BMJ Open's open peer review process please email info.bmjopen@bmj.com

BMJ Open

A systematic review of changed smoking behavior, smoking cessation, and psychological states of smokers during the COVID-19 pandemic

Journal:	*BMJ Open*
Manuscript ID	bmjopen-2021-055179
Article Type:	Original research
Date Submitted by the Author:	10-Jul-2021
Complete List of Authors:	Chun, Hae-ryong; Seoul National University,<br>Cheon, Eunsil; Seoul National University, Graduate school of public health<br>Hwang, Ji-eun; Dankook University
Keywords:	COVID-19, Public health < INFECTIOUS DISEASES, Anxiety disorders < PSYCHIATRY

SCHOLARONE™
Manuscripts

I, the Submitting Author has the right to grant and does grant on behalf of all authors of the Work (as defined in the below author licence), an exclusive licence and/or a non-exclusive licence for contributions from authors who are: i) UK Crown employees; ii) where BMJ has agreed a CC-BY licence shall apply, and/or iii) in accordance with the terms applicable for US Federal Government officers or employees acting as part of their official duties; on a worldwide, perpetual, irrevocable, royalty-free basis to BMJ Publishing Group Ltd ("BMJ") its licensees and where the relevant Journal is co-owned by BMJ to the co-owners of the Journal, to publish the Work in this journal and any other BMJ products and to exploit all rights, as set out in our licence.

The Submitting Author accepts and understands that any supply made under these terms is made by BMJ to the Submitting Author unless you are acting as an employee on behalf of your employer or a postgraduate student of an affiliated institution which is paying any applicable article publishing charge ("APC") for Open Access articles. Where the Submitting Author wishes to make the Work available on an Open Access basis (and intends to pay the relevant APC), the terms of reuse of such Open Access shall be governed by a Creative Commons licence – details of these licences and which Creative Commons licence will apply to this Work are set out in our licence referred to above.

Other than as permitted in any relevant BMJ Author's Self Archiving Policies, I confirm this Work has not been accepted for publication elsewhere, is not being considered for publication elsewhere and does not duplicate material already published. I confirm all authors consent to publication of this Work and authorise the granting of this licence.

For peer review only

A systematic review of changed smoking behavior, smoking cessation, and psychological states of smokers during the COVID-19 pandemic

1 Chun, Hae-ryong; Seoul National University, Graduate school of public health; mamimi777@snu.ac.kr

2 Cheon, Eunsil; Seoul National University, Graduate school of public health; silverberry2@snu.ac.kr

* Correspondence to: Jieun Hwang Dankook University College of Health Science, 119, Dandae-ro, Dongnam-gu, Cheonan-si, Chungcheongnam-do, Republic of Korea loshjeve@gmail.com

Word count : 3115

For peer review only

ABSTRACT

Objectives : Although the global COVID-19 pandemic has increased interest in research involving high-risk smokers, studies examining altered smoking behaviors, quit attempts, and associated psychological factors among smokers are still scarce. This study intends to systematically review the literature related to this subject.

Design A systematic review of published Articles on cigarettes and COVID-19-related topics

Data Sources : We found papers using the keywords COVID-19, cigarettes, electronic cigarettes (e-cigarettes), and psychological factors in PubMed and ScienceDirect DB.

Data selection : We included Articles on cigarettes and COVID-19-related topics in the full text, written in English, and having surveyed adults.

Data extraction and synthesis : Papers of low quality based on quality assessment were excluded. 13 papers were related to smoking behavioral content. Furthermore, nine papers were related to smoking cessation and four papers were related to psychological factors.

For peer review only - http://bmjopen.bmj.com/site/about/guidelines.xhtml

Results : Due to the lockdown, there was a habit of purchasing large quantities of cigarettes in advance. After the pandemic, smokers who only smoke cigarettes have increased their attempts to quit smoking and their willingness to quit, compared with e-cigarette users.

Conclusions : Due to the COVID-19 outbreak, the intention to quit smoking was different among smokers, according to the type of cigarettes (cigarette only users, electronic cigarette users, dual users). As the COVID-19 pandemic continues, policies and campaigns that could increase smoking cessation intentions and attempts to quit smoking among smokers who are at high risk of COVID-19 should be added. For e-cigarette users with poor health-seeking behavior, interventions to increase the intention to quit smoking are needed.

Keyword : COVID-19 Virus Disease, Quitting Smoking, Smoking Habits

STRENGTHS AND LIMITATIONS OF THIS STUDY

- The limitation of this study is that the papers collected for this systematic review were cross-sectional in design.
- This made it difficult to determine causality, and only their relevance was presented.
- This study is the first to systematically review the literature regarding changes in smoking behaviors, cessation intentions, and psychological changes in smokers after the COVID-19 pandemic.

1. INTRODUCTION

On January 30, 2020, the World Health Organization (WHO) declared COVID-19, a disease of the respiratory tract characterized by a severe acute respiratory syndrome,[1] as a public health emergency of international concern (PHEIC).[2] In 2020, a total of 82,357,480 cases of COVID-19 were reported, including 1,802,390 deaths (https://covid19.who.int/). Due to the pandemic, different standards and steps to control the spread of the disease have been adopted around the world, but lockdown and social distancing measures to prevent COVID-19 infection have been taken by almost all countries.

Smoking is a high-risk factor for COVID-19, and there are many systematic reviews and meta-analyses related to the association between COVID-19 and Smoking. Because tobacco is associated with chronic lung disease, physicians could expect more smokers to develop the severe form of COVID-19[3] given the effect of tobacco on respiratory disease and immune function.[4] Smoking is associated with negative progression and side effects of COVID-19.[5] smoking, including current or previous smoking history, significantly increases the severity and risk of death from COVID-19.[6] Previous studies have suggested a number of public health messages to continue focusing on smoking cessation efforts, but smoking rates have not decreased.[7]

A small number of investigations have been conducted on whether smoking cessation efforts

For peer review only

For peer review only - http://bmjopen.bmj.com/site/about/guidelines.xhtml

or behavior have changed since COVID-19, and what psychological factors are related to COVID-19 that affect smoking behavior change. It is expected that smoking behavior will change given the difficulty in freely purchasing tobacco products as non-essential products, as they are locked down under the pandemic, as well as the need to stay at home for prolonged periods of time.

Changes in daily life due to social distancing and lockdowns are expected to help reduce smoking consumption.[8] However, people experience higher levels of stress than usual with social isolation, employment, finances, responsibilities, and concerns about becoming ill from the virus.[9] Therefore, many smokers smoke to relieve this stress and these negative emotions,[10] but it is often counterproductive.[11] Previous research has shown that stress, anxiety, and depression can influence tobacco use behavior.[12] Currently, there are insufficient studies on psychological pain after COVID-19, and further research is needed because the relationship between psychological pain and health behavior is unclear.[13]

During the COVID-19 lockdown,[14] the daily use of e-cigarettes among e-cigarette smokers slightly increased; therefore, it is necessary to investigate whether smoking behavior changes depending on the type of cigarette. Cigarette users are aware that they are engaging in behaviors that are harmful to their health. On the other hand, it is not yet clear whether e-cigarettes help smokers quit smoking[15] and e-cigarette users believe e-cigarettes are less harmful to health as they contain relatively less harmful ingredients than cigarettes.[16] They believe that smoking e-cigarettes is in itself an effort to quit smoking.[16] Compared to studies examining whether smoking is a high-risk factor for COVID-19[5, 6], studies examining whether smoking behavior and cessation intentions have changed since COVID-19 have not yet been published. Therefore, in this study, we systematically reviewed the literature to determine whether there were any changes in smoking behavior, cessation intentions, and psychological aspects, depending on the products used in the COVID-19 epidemic. In the future, it is expected that this thesis will establish a scientific basis, and the policy response according to the types of cigarettes, for the continued COVID-19 pandemic, and even in the era post- COVID-19.

2. METHODS

2.1. Literature Search Strategy

A systematic review was performed according to the Preferred Reporting Items for Systematic Reviews and Meta-Analyses (PRISMA).

A search was performed using all entry terms from COVID-19, depression, stress, psychological, anxiety, tobacco, tobacco products, tobacco use, and electronic nicotine delivery system. Only papers published between January and December 2020 were included. All entry terms are listed in Supplementary Table 1.

A literature search was conducted on PubMed using the following search terms: "(depression OR stress OR anxiety OR psychology) AND ((tobacco OR e-cig) AND COVID-19))" in the title or the abstract in 2020. A literature search was conducted on Science Direct using the

For peer review only - http://bmjopen.bmj.com/site/about/guidelines.xhtml

following search terms : "COVID-19 AND (e-cigarette OR "electronic cigarette" OR "electronic nicotine delivery" OR "vaping" OR "heat not burn" OR "heated tobacco product")," as well as COVID-19 AND (tobaccos OR cigarette OR kretek OR bidis OR "pipe tobacco" OR cigarillos).

2.2. Patient and public involvement

Patients and the public were not involved in the entire process of conducting this study.

2.3. Inclusion and Exclusion Criteria

Figure 1 shows a flowchart of the database searches, followed by the exclusion/inclusion strategy. In the first phase (Figure 1), certain types of articles were excluded, including comments, letters, editorials, viewpoints, correspondence, and articles without full text. In addition, duplicate works and those not written in English, as well as studies that did not include humans or adults, were also excluded. In the next phase (Figure 1), studies which were not related to tobacco or COVID-19 were further excluded. In addition, articles were removed if the topics were not related to changes in tobacco use and mental health caused by COVID-19. Finally, articles with a high risk of bias were removed using the Risk of Bias Assessment tool for Non-randomized Studies (RoBANS).[17]

2.4. Data Extraction

Two independent reviewers (HR and JE) screened the literature and assessed each study by reading titles, abstracts, and full texts. Data from studies that fulfilled our inclusion criteria were extracted independently by three authors (HR, JE, and ES). The main data points included were the following: study details (author, journal, publication date, country, study design, study period, and funding), total number of participants, types of cigarettes, and changes in smoking behavior.

Two authors (HR and ES) independently assessed the quality of included studies using the Risk of Bias Assessment tool for Non-randomized Studies (RoBANS) [17] which showed moderate reliability, promising feasibility, and validity. If the opinions of two authors were different, the quality evaluation was completed through further discussion with another researcher (JE). RoBANS contains six domains: selection of participants, confounding variables, measurement of exposure, blinding of outcome assessments, incomplete outcome data, and selective outcome reporting(figure 2, figure 3). The level of risk of bias was divided into three stages (high risk, unclear risk, and low risk). When the risk of bias in the literature was high, it was excluded.

3. RESULTS

For peer review only - http://bmjopen.bmj.com/site/about/guidelines.xhtml

3.1. **Literature Search and Literature Selection Results**

A total of 1,659 papers were searched in PubMed and Science Direct DB and 905 non-original papers were excluded. Eleven papers without full text and four papers not written in English were excluded. In addition, 413 papers were selected, excluding 102 papers that were not studied in humans, 114 papers that were not studied in adults, and 110 duplicate papers after the first screening phase. Literature unrelated to tobacco (n =200) and COVID-19 (n=21) were excluded. In addition, a total of 18 papers were included, excluding 166 articles that were not related to tobacco behavior and psychology that changed after the pandemic as well as eight articles targeting COVID-19 patients or medical personnel through the initial phase.

3.2. **Assessment Result of the risk of bias**

A total of 13 articles were selected after excluding five articles with a high risk of bias, as determined through quality evaluation of the 18 articles using the RoBANS.

3.3. Characteristics of included studies by three participants

All 13 selected studies were published in 2020, with 11 studies having surveyed the population from March to May, one from June to July, and one study in August. Three papers were conducted in the United States, three in the UK, two in Italy, two in Turkey, and one each in China, India, and Australia. All were cross-sectional design studies using surveys. The only accepted papers were articles and not articles, such as comments, letters, editorials, viewpoints, and correspondences. According to the literature, the number of study participants varied from 93 to 52,002, and all participants were adults. Eight papers only targeted cigarette smokers, and five articles reviewed e-cigarette and cigarette users together (Table 1).

3.4. Characteristics of included studies by three participants

Table 1 shows that smokers' smoking behavior, smoking cessation intentions, and psychological changes changed due to the COVID-19 pandemic, presented in 13 papers. Seven papers studied changes in smoking behavior, eight papers that examined changes in perception of smoking, and five papers that examined changes in smokers' psychological state after the COVID-19 outbreak (Table 1).

3.4.1. **Changes in smoking behavior in e-cigarette users (or dual users) and cigarette users after the COVID-19 outbreak**

According to the article by E.K. Soule et al.[18], the higher the reliance on e-cigarettes, the more was the incidence of cigarette use. There are also increasing efforts to reduce e-cigarette use among dual users; however, e-cigarette skills are increasing, and e-cigarette use is increasing. According to the article by P.Caponnetto et al.[19], daily cigarette consumption by dual users of

For peer review only

cigarettes, e-cigarettes, and cigarette users decreased slightly. Due to the outbreak of COVID-19, the method of purchasing products has changed by buying a large number of products in advance.

According to the results of article by R. Cancello et al.[7] and Ren et al.[20], tobacco consumption increased by 38% and 30%, respectively. The article by Chopra et al. also shows a significant decrease in the amount of smoking consumption compared to before the COVID-19 pandemic (0.02 [0.03], P <0.05). [21] However, the article by Ozcelik et al.,[22] 67.5% said there was no change in smoking behavior, which cannot be interpreted as a significant change in smoking behavior after the COVID-19 pandemic.

3.4.2. Changes in intentions and attempts to quit smoking among e-cigarette users (or dual users) and cigarette users after the COVID-19 outbreak

H. Tattan-Birch et al.[23] found that only a few smokers have attempted to quit smoking due to the COVID-19 outbreak. Approximately one in 10 current e-cigarette users reported an attempt to quit vaping because of COVID-19. E.K. Soule et al. reported that users with high reliance on e-cigarettes exerted less effort to reduce product usage than users with low reliance. According to the article by P.Caponnetto et al.[24], while most exclusive cigarette smokers have considered quitting smoking, most exclusive e-cigarette users have not considered stopping e-cigarette use. According to the article by Chertok et al.,[25] 36.7% of all smokers attempted to quit smoking after the pandemic regardless of cigarette type because people who quit smoking due to COVID-19 perceived smoking as a high-risk factor for COVID-19 infection.

According to Jackson et al.[26], the COVID-19 shutdown was associated with increased attempts to quit smoking. According to the results of the article by Kayhan Tetik et al.[27], which compared the success rates of smoking cessation before and after the pandemic, the COVID-19 outbreak effectively promoted smoking cessation. Only 12.8% of people who quit smoking after the COVID-19 outbreak did not restart smoking. According to Ozcelik et al.,[22] people with coronaphobia exhibited a significantly higher decrease or cessation of smoking than those with no change in smoking behavior.

3.4.3. The association between smoking and psychological changes in e-cigarette users (or dual users) and cigarette users after the COVID-19 outbreak

According to E.K. Soule et al.[18], the greater the dependence on e-cigarettes, the higher the concern about COVID-19 infection than those with low dependence. In the article by S.D. Kowitt et al.,[28] the higher the awareness regarding the risk of contracting COVID-19, regardless of cigarette type, the greater was the willingness to quit ($B = 0.38$, $p < 0.001$) and the attempt to quit smoking (aOR: 1.31, 95% CI: 1.04, 1.64). In addition, as social distancing ($B = 0.11$, $p = 0.01$) was enforced more strongly, the willingness to quit smoking increased.

R. Stanton et al.[29] found that people with increased tobacco consumption had a higher risk of depression, anxiety, and stress. Jackson et al.[30] reported that current and former smokers had

For peer review only - http://bmjopen.bmj.com/site/about/guidelines.xhtml

increased stress from COVID-19 than non-smokers. Ozcelik et al.[22] indicated that among people with coronaphobia, those who reduced or stopped smoking felt more anxious than those who increased their smoking frequency.

For peer review only

For peer review only - http://bmjopen.bmj.com/site/about/guidelines.xhtml

Table 1 Characteristics of included studies by three subjects

First Author, Setting Period	Type of cigarette(N,%)	Subject 1* Smoking behavior	Subject 2* Smoking cessation	Subject 3* Psychological effect
H. Tattan-Birch[23] England April-May	Total : 3,179<br>-Smoking cigarette<br>never:1,804 (56.7%)<br>long term ex : 834 (26.2%)<br>recent ex : 72 (2.3%)<br>current smoker : 469 (14.8%)<br>- E-cig use<br>no current use : 2,987 (94%)<br>current use : 192 (6%)		Quit Attempt<br>- Cigarette smoker: 12.2%<br>- E-cig: 10%	
E.K. Soule[18] US April	-Total : E-cig use (n=93)<br>- Dual user (E-cig and cigarette use)<br>1) Cigarettes : 50<br>2) Cigar : 11<br>3) Cigarillo or little cigar : 19<br>4) Smokeless : 7<br>5) Waterpipe : 11	1. Efforts to reduce the use of e-cigs have increased for dual users (M = 3.71 vs. M = 3.02; p < 0.05), but e-cig use skills have increased (M = 3.48 vs. M = 2.27; p < 0.05) and usage has increased (M = 4.34 vs. M = 3.72;p < 0.05).<br>2. Users with high dependence on e-cigs have increased use of e-cigs compared to users with low dependence.<br>(M = 4.62 vs. M = 3.35; p < 0.001)	Users who depended more on e-cigs (M = 3.10, SD = 0.63) had less effort to reduce their use of e-cigs than users who were less dependent on e-cigs (M = 3.79, SD = 0.61; p < 0.02).	Users who rely heavily on e-cigs are more concerned about COVID-19 than users who do not rely heavily on e-cigs<br>(M = 3.86 vs. M = 3.06; <0.05).
P.Caponnetto[24] Italy 2nd-26th April	Total : 1,825<br>Dual user of cigarette and heated tobacco products(33,1.8%)<br>Dual user of cigarette and e-cig(64,3.5%)<br>Former smoker(293, 16%)<br>Exclusive cigarette smoker(582, 32%)<br>Never smoker(547, 30%)<br>Exclusive user of heated tobacco products(81,4.4%)<br>Exclusive user of e-cig (225, 12.3%)	1. Dual users of cigarette and e-cig and exclusive cigarette smokers (sr value (-2.7 and -1.9)) daily consumption has decreased.<br>2. "Exclusive combustible tobacco users" and the "Exclusive Ecig users" changed the way of purchasing products and the majority of them changed their habits by having large stocks of cigarettes or e-liquids/ cartridges at home to avoid going out every day.	Most exclusive cigarette smokers(sr=11.6) have considered quitting but most exclusive e-cig users(sr=-4.2) have not considered stopping the use of e-cigs.	
R. Cancello[7] Italy 15th April-4th May	Total: 490<br>Cigarette smoker: 105(21.4%)	Cigarette Consumption increase: 38%		
S.D. Kowitt[28] US 23rd April-7th May	Total: 777<br>Cigarette 651 (83.8%)<br>E-cig 293(37.3%)<br>Smokeless tobacco 170(21.9%)<br>Waterpipe tobacco 103(13.3%)			(Not classified according to the type of cigarette, but surveyed for all smokers.)<br>-Having higher risk perceptions of COVID-19 was associated with higher quit intentions<br>(B = 0.38, p < 0.001)<br>-Higher odds of quit attempt since COVID-19 started (aOR: 1.31, 95% CI: 1.04, 1.64).<br>-Greater social distancing efforts were associated with higher quit intentions<br>(B = 0.11, p = 0.01).
R. Stanton[29] Australia April	Total = 1,491<br>Cigarette Smoker = 172(11.5%)			Those who reported a negative change in smoking, they were more likely to have higher depression (aOR = 1.09, 95% CI = 1.04, 1.13), anxiety (aOR = 1.12, 95% CI = 1.06, 1.18), and stress (aOR = 1.10, 95% CI = 1.05, 1.15).
Jackson[30] England 21st March-20th April	Total : 53,002<br>Ex-smokers(cigarette):13,602(25.7%)<br>Current smokers(cigarette):8,057(15.2%)			Current and ex-smokers had higher odds than never smokers of reporting significant stress about becoming seriously ill from COVID-19 (current: OR=1.34 (1.27–1.43); ex-smokers: OR=1.22 (1.16–1.28)).

For peer review only - http://bmjopen.bmj.com/site/about/guidelines.xhtml

First Author, Setting Period	Type of cigarette(N,%)	Subject 1* Smoking behavior	Subject 2* Smoking cessation	Subject 3* Psychological effect
Jackson[26] England March-April	Total Cigarette users: 20,558	The COVID-19 lockdown was not associated with a significant change in smoking prevalence 17.0% (after) versus 15.9% (before), OR = 1.09, 95% CI = 0.95–1.24.	associated with increases in quit attempts 39.6 vs 29.1%, aOR = 1.56, 95% CI=1.23–1.98, quit success 21.3 vs 13.9%, aOR = 2.01, 95% CI = 1.22–3.33 and cessation (8.8 vs 4.1%, aOR = 2.63, 95% CI = 1.69–4.09) among past-year smokers	
Kayhan Tetik[31] Turkey May	Total Cigarette users: 357		165(46.2%) smokers of participants, 86 (24.1%) did not quit despite this pandemic.	
Ren[20] China 14th February -29th March	Total Cigarette users:1,172	Some (30.1%) increased smoking during the pandemic.		
Ozcelik[22] Turkey 1st January-30th June	Cigarette only Current smoker (N= 111, 98%) Quitted smoking(N= 3, 2%)	77 people (67.5%) had no behavioral change associated with smoking	People with coronaphobia exhibited a significantly higher decrease or cessation of smoking than no change in smoking behavior (p: .006, odds ratio: 18.222 (2.301–144.305).	Decrease or cessation of smoking in people with coronaphobia was found significantly greater in the individuals who had severe anxiety than individuals who increased smoking (p: .36, odds ratio:10.667 [1.171–97.185]).
Chertok[25] US Ohio April	Total : 810 1. Cigarettes only: 115 (62.8%) 2. Both cigarettes and e-cig: 68(37.2%) 3. Not smoker: 627		(Not classified according to the type of cigarette, but surveyed for all smokers.) - Attempt to quit : 36.7% (n = 66) - Those who quit since COVID-19, those who desired to quit, perceived higher risk of infection.	
Chopra[21] India 15th August-30th August	No smoking: 939(94.4%) Cigarette user: 56	Smoking consumption (0.02 [0.03], P <0.05) is significantly reduced.		

* Subject 1 is a case in which an article contains changes in smoking behavior before and after the COVID-19 epidemic. Subject 2 is a case in which an article contains perceptions of smoking or cessation before and after the COVID-19 epidemic. And Subject 3 refers to points in which an article contains information on changed psychological states such as depression, stress, and anxiety before and after the COVID-19 epidemic.

For peer review only - http://bmjopen.bmj.com/site/about/guidelines.xhtml

4. DISCUSSION

We examined the changes in smoking behavior, quitting intentions, and psychological changes caused by the COVID-19 outbreak in smokers (cigarette users, e-cigarette users, and dual users). The smoking behavior of cigarette users has not changed significantly due to COVID-19, but the amount of smoking done by e-cigarette-only users has increased with escalated dependency. For dual users who use cigarettes and e-cigarettes together, efforts to reduce product usage have increased, but there was a tendency to buy cigarettes in bulk, increase e-cigarette usage skills, as well as increased their use. This is thought to be the result of a tendency to recognize that using e-cigarettes is relatively less harmful to health than smoking cigarettes.[16] According to previous studies, many e-cigarette smokers recognize that e-cigarettes are relatively less harmful to health and others [16] because they contain far fewer harmful components [32] than cigarettes. However, it is not yet clear to what extent e-cigarettes can help smokers quit smoking.[15] For this reason, e-cigarette users should consider the risks of using e-cigarettes as much as cigarettes.

Cigarette users' efforts to reduce product usage and attempts to quit smoking after the pandemic have increased, but efforts to reduce product usage by e-cigarette users have decreased. In particular, the higher the dependence of e-cigarette users, the higher were the worries related to COVID-19, but the efforts to reduce the amount of smoking were lower than users with low e-cigarette dependence. The reason for this could be that e-cigarette users think that using e-cigarettes is an attempt to quit smoking [16]. Therefore, it is judged that e-cigarette users' intentions to quit smoking have not increased even during the pandemic. However, a cohort analysis done in the United States found that e-cigarette users did not quit smoking compared to smokers who did not use e-cigarettes, and that more than half of e-cigarette users still used e-cigarettes a year later[33]. In another study, it was not yet clear whether e-cigarette users were more likely to quit smoking.[34] Since many studies are still being conducted on the cessation effect of e-cigarettes, it should be recognized that e-cigarette use could be harmful to health.

Regardless of the cigarette type, the higher the awareness of the risks associated with contracting COVID-19, the higher the level of social distancing since the start of COVID-19, and the higher the intent to quit smoking. In the case of cigarette tobacco users, the presence of coronaphobia increased smoking cessation intentions. Among them, those who reduced smoking felt more anxious than those who increased their amount of smoking. Smokers were more likely to quit after understanding the health risks of smoking due to the "vulnerability hypothesis," wherein smokers who care about their health would be inclined to quit smoking when they know it makes them vulnerable to certain diseases.[35, 36] This presents the same context as the results of this systematic review.

In contrast, previous studies have shown that smokers have higher levels of depression, anxiety, and stress, and also have increased smoking volumes. According to a article by Klemperer et al. [37] although 28.3% of patients reduced their tobacco use due to fear of infectious diseases, while 30.3% showed increased tobacco use, the anxiety levels were high for both. However, in the literature surveyed in this study, it was found that the higher the level of anxiety, the higher the attempt to quit smoking. Therefore, further research exploring the relationship between

For peer review only - http://bmjopen.bmj.com/site/about/guidelines.xhtml

mental health and smoking behaviors during the COVID-19 pandemic using standardized tools to measure mental health conditions, such as anxiety, is needed.

Increased awareness of COVID-19 risk has led to escalated smoking cessation attempts, which can be considered a desirable situation for physical health in the long run. However, for mental health, including the anxiety, depression, and increased stress during the pandemic, the government's response of conducting campaigns for risk awareness is required to protect their citizens' mental health as a way to address the increasing trend of smoking.

It is also meaningful that this study looked at usage behavior after the COVID-19 pandemic based on the type of cigarette. Research on the link between cigarette smoking and COVID-19 is increasing, and WHO also suggests that smokers may be considered at high risk for contracting COVID-19. However, due to the lack of research on the relationship between the use of liquid electronic cigarettes and COVID-19, the willingness or attempts to quit electronic cigarette use has not increased compared to cigarette-only smokers or dual users. As smokers have a high risk for contracting certain diseases both during the COVID-19 epidemic and after it, further studies on the risk of e-cigarettes and their impact on smoking cessation, especially regarding getting infected with COVID-19, should be conducted.

5. CONCLUSION

In this study, we examined smoking behaviors, smoking cessation intentions, and the psychological states of smokers who changed after COVID-19 according to cigarette type. We conducted a systematic review of 13 papers to suggest future research and policy directions that the government may implement for COVID-19 and smokers. During the COVID-19 pandemic, cigarette users' consumption did not change significantly, but dual users and e-cigarette users with a high dependence on e-cigarettes showed increased consumption. Cigarette smokers' quit intentions, attempts, and success rates of cessation have increased, and even those with coronaphobia showed increased intent in quitting smoking. On the other hand, users of e-cigarettes demonstrated fewer attempts to quit. As the COVID-19 pandemic continues, policies and campaigns should be implemented to increase the intent and attempts to stop. Research and interventions on COVID-19, Smoking, and e-cigarette use should also be conducted in the medium to long term.

CONTRIBUTIORS

HC designed the study, analyzed and interpreted the data, and wrote the manuscript. JH and EJ contributed to the study design and interpretation of the results.

FUNDING

This study was supported by the National Research Foundation of Korea (NRF) grant funded by Ministry of Science and ICT (MSIT) (No. 2020R1C1C1012562).

COMPETING INTERESTS

For peer review only - http://bmjopen.bmj.com/site/about/guidelines.xhtml

None.

PATIENT CONSENT FOR PUBLICATION

Not required.

ETHICS APPROVAL

This systematic review was based on published data. As researchers did not access any information that could lead to the identification of an individual patient, no concerning ethical issue was raised in this research. Therefore, obtaining ethical approval and consent of participants was waived.

PROVENANCE AND PEER REVIEW

Not commissioned; externally peer reviewed.

DATA SHARING STATEMENT

The data set used and/or analysed for this review will be available from the corresponding author upon a reasonable request.

REFERENCES

1. Berlin I, Thomas D, Le Faou A-L, Cornuz J. COVID-19 and smoking. *Nicotine and Tobacco Research*. 2020;22(9):1650-1652.
2. Organization WH. COVID 19 Public Health Emergency of International Concern (PHEIC). Global research and innovation forum: towards a research roadmap. 2020;
3. Ni Y, Shi G, Qu J. Indoor PM2. 5, tobacco smoking and chronic lung diseases: A narrative review. *Environmental research*. 2020;181:108910.
4. Health UDo, Services H. The health consequences of smoking—50 years of progress: a report of the Surgeon General. Atlanta, GA: US Department of Health and Human Services, Centers for Disease …; 2014.
5. Vardavas CI, Nikitara K. COVID-19 and smoking: A systematic review of the evidence. *Tobacco induced diseases*. 2020;18
6. Umnuaypornlert A, Kanchanasurakit S, Lucero-Prisno DEI, Saokaew S. Smoking and risk of negative outcomes among COVID-19 patients: A systematic review and meta-analysis. *Tobacco induced diseases*. 2021;19
7. Cancello R, Soranna D, Zambra G, Zambon A, Invitti C. Determinants of the lifestyle changes during COVID-19 pandemic in the residents of Northern Italy. *International journal of environmental research and public health*. 2020;17(17):6287.
8. Wood W, Tam L, Witt MG. Changing circumstances, disrupting habits. *Journal of personality and social psychology*. 2005;88(6):918.
9. Fancourt D, Bu F, Mak HW, Steptoe A. COVID-19 social study. *Results release*. 2020;15
10. Volkow ND. Collision of the COVID-19 and addiction epidemics. American College of Physicians; 2020.
11. Parrott AC. Does cigarette smoking cause stress? *American Psychologist*. 1999;54(10):817.
12. García-Álvarez L, Fuente-Tomás L, Sáiz PA, García-Portilla MP, Bobes J. Will changes in alcohol and tobacco use be seen during the COVID-19 lockdown? *Adicciones*. Apr 1 2020;32(2):85-89. ¿Se observarán cambios en el consumo de alcohol y tabaco durante el confinamiento por COVID-19? doi:10.20882/adicciones.1546
13. Stanton R, To QG, Khalesi S, et al. Depression, Anxiety and Stress during COVID-19: Associations with Changes in Physical Activity, Sleep, Tobacco and Alcohol Use in Australian Adults. *Int J Environ Res Public Health*. Jun 7 2020;17(11)doi:10.3390/ijerph17114065
14. Adriaens K, Van Gucht D, Baeyens F. Differences between dual users and switchers center around

For peer review only - http://bmjopen.bmj.com/site/about/guidelines.xhtml

vaping behavior and its experiences rather than beliefs and attitudes. *International journal of environmental research and public health*. 2018;15(1):12.
15. Grana RA, Popova L, Ling PM. A longitudinal analysis of electronic cigarette use and smoking cessation. *JAMA internal medicine*. 2014;174(5):812-813.
16. Etter JF, Bullen C. Electronic cigarette: users profile, utilization, satisfaction and perceived efficacy. *Addiction*. 2011;106(11):2017-2028.
17. Kim SY, Park JE, Lee YJ, et al. Testing a tool for assessing the risk of bias for nonrandomized studies showed moderate reliability and promising validity. *Journal of clinical epidemiology*. 2013;66(4):408-414.
18. Soule EK, Mayne S, Snipes W, Guy MC, Breland A, Fagan P. Impacts of COVID-19 on electronic cigarette purchasing, use and related behaviors. *International Journal of Environmental Research and Public Health*. 2020;17(18):6762.
19. Caponnetto P, Inguscio L, Saitta C, Maglia M, Benfatto F, Polosa R. Smoking behavior and psychological dynamics during COVID-19 social distancing and stay-at-home policies: A survey. *Health Psychol Res*. May 27 2020;8(1):9124. doi:10.4081/hpr.2020.9124
20. Ren Y, Qian W, Li Z, et al. Public mental health under the long-term influence of COVID-19 in China: Geographical and temporal distribution. *Journal of Affective Disorders*. 2020;277:893-900.
21. Chopra S, Ranjan P, Singh V, et al. Impact of COVID-19 on lifestyle-related behaviours-a cross-sectional audit of responses from nine hundred and ninety-five participants from India. *Diabetes & Metabolic Syndrome: Clinical Research & Reviews*. 2020;14(6):2021-2030.
22. Ozcelik N, Yilmaz Kara B. Effect of coronaphobia on smoking habits. *Journal of addictive diseases*. 2020:1-9.
23. Tattan-Birch H, Perski O, Jackson S, Shahab L, West R, Brown J. COVID-19, smoking, vaping and quitting: a representative population survey in England. *Addiction*. 2020;
24. Caponnetto P, Inguscio L, Saitta C, Maglia M, Benfatto F, Polosa R. Smoking behavior and psychological dynamics during COVID-19 social distancing and stay-at-home policies: A survey. *Health psychology research*. 2020;8(1)
25. Chertok IRA. Perceived risk of infection and smoking behavior change during COVID-19 in Ohio. *Public Health Nursing*. 2020;37(6):854-862.
26. Jackson SE, Garnett C, Shahab L, Oldham M, Brown J. Association of the Covid-19 lockdown with smoking, drinking, and attempts to quit in England: an analysis of 2019-2020 data. *medRxiv*. 2020;
27. Kayhan Tetik B, Gedik Tekinemre I, Taş S. The Effect of the COVID-19 Pandemic on Smoking Cessation Success. *J Community Health*. Jul 8 2020:1-5. doi:10.1007/s10900-020-00880-2
28. Kowitt SD, Cornacchione Ross J, Jarman KL, et al. Tobacco quit intentions and behaviors among cigar smokers in the United States in response to COVID-19. *International journal of environmental research and public health*. 2020;17(15):5368.
29. Stanton R, To QG, Khalesi S, et al. Depression, anxiety and stress during COVID-19: associations with changes in physical activity, sleep, tobacco and alcohol use in Australian adults. *International journal of environmental research and public health*. 2020;17(11):4065.
30. Jackson SE, Brown J, Shahab L, Steptoe A, Fancourt D. COVID-19, smoking and inequalities: a study of 53 002 adults in the UK. *Tobacco control*. 2020;
31. Tetik BK, Tekinemre IG, Taş S. The effect of the COVID-19 pandemic on smoking cessation success. *Journal of Community Health*. 2020:1-5.
32. Goniewicz ML, Knysak J, Gawron M, et al. Levels of selected carcinogens and toxicants in vapour from electronic cigarettes. *Tobacco control*. 2014;23(2):133-139.
33. Pierce JP, Benmarhnia T, Chen R, et al. Role of e-cigarettes and pharmacotherapy during attempts to quit cigarette smoking: The PATH Study 2013-16. *PloS one*. 2020;15(9):e0237938.
34. Pasquereau A, Guignard R, Andler R, Nguyen-Thanh V. Electronic cigarettes, quit attempts and smoking cessation: a 6-month follow-up. *Addiction*. 2017;112(9):1620-1628.
35. Borrelli B, Hayes RB, Dunsiger S, Fava JL. Risk perception and smoking behavior in medically ill smokers: a prospective study. *Addiction*. 2010;105(6):1100-1108.
36. Weinstein ND. The precaution adoption process. *Health psychology*. 1988;7(4):355.
37. Klemperer EM, West JC, Peasley-Miklus C, Villanti AC. Change in Tobacco and Electronic Cigarette Use and Motivation to Quit in Response to COVID-19. *Nicotine Tob Res*. Aug 24 2020;22(9):1662-1663. doi:10.1093/ntr/ntaa072

For peer review only - http://bmjopen.bmj.com/site/about/guidelines.xhtml

Figure legend/caption

Figure 1 Flow Chart Diagram

Caption : The flowchart shows the articles selection process, the criteria for exclusion of articles, and the number of articles excluded.

Figure 2 Assessment of the risk of bias in identified studies using the RoBANS questionnaire

Caption : Author judgment for each RoBANS domain for included articles. Green indicates a low risk of bias, yellow indicates a high risk of ambiguity, and red indicates a high risk of bias. The 6 domains are "Comparability of groups, Participant selection, Confounding variables, Measurement of exposure, Blinding of the outcome, Incomplete outcome data, Selective outcome reporting."

Figure 3 Assessment Summary of the risk of bias in identified studies using the RoBANS questionnaire

Caption : Author judgment for each RoBANS domain for included articles. Green circles indicate low risk of bias, yellow circles indicate that the risk is unclear, and a red circles indicate high risk of bias. Study numbers indicate the order of the articles presented in Table 1.

For peer review only

For peer review only - http://bmjopen.bmj.com/site/about/guidelines.xhtml

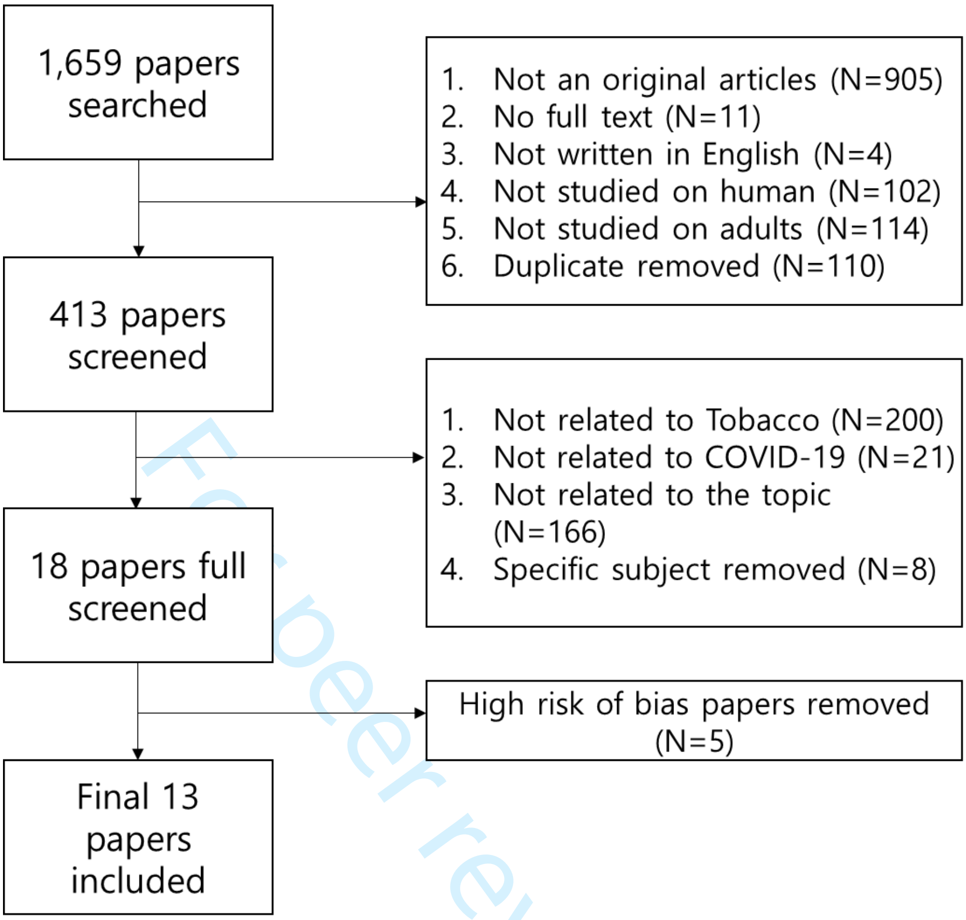
1,659 papers searched
1. Not an original articles (N=905)
2. No full text (N=11)
3. Not written in English (N=4)
4. Not studied on human (N=102)
5. Not studied on adults (N=114)
6. Duplicate removed (N=110)
413 papers screened
1. Not related to Tobacco (N=200)
2. Not related to COVID-19 (N=21)
3. Not related to the topic (N=166)
4. Specific subject removed (N=8)
18 papers full screened
High risk of bias papers removed (N=5)
Final 13 papers included

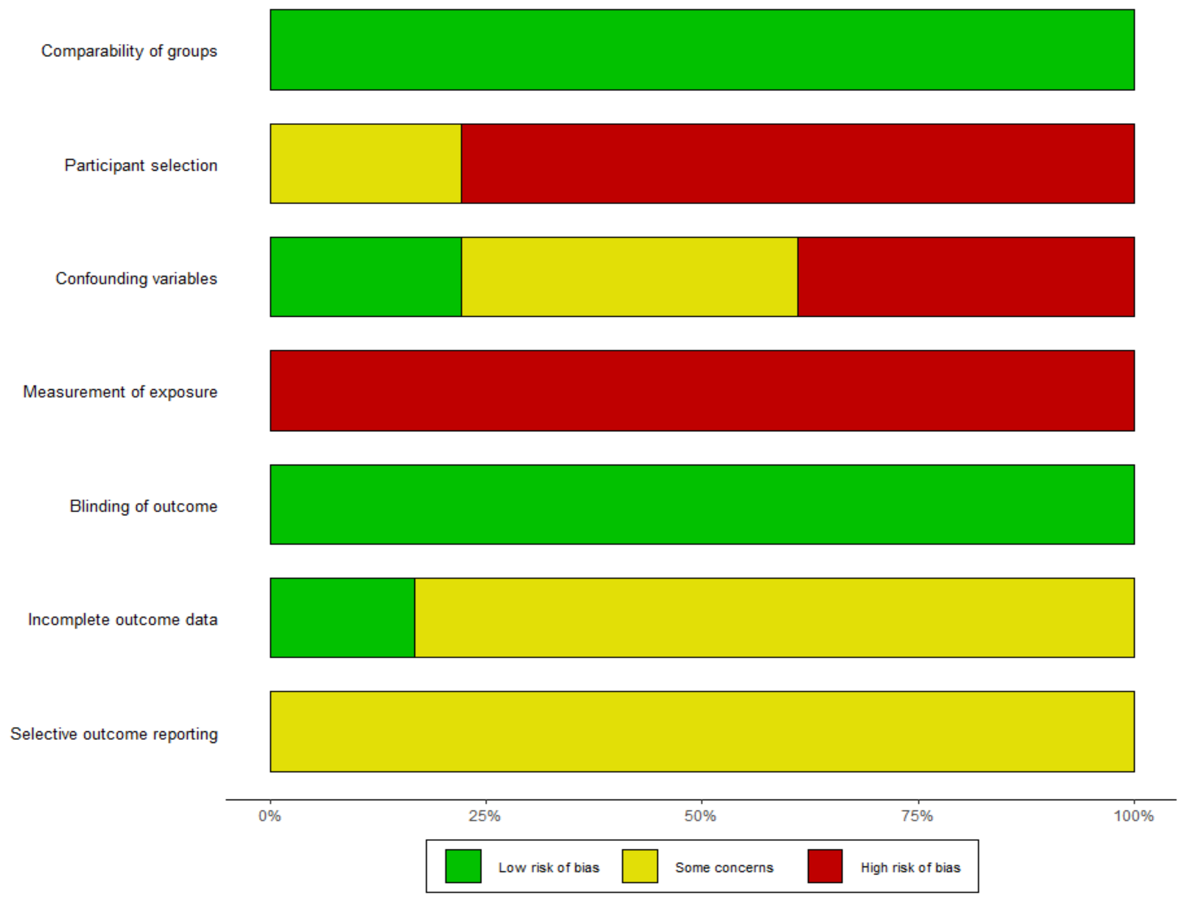

For peer review only - http://bmjopen.bmj.com/site/about/guidelines.xhtml

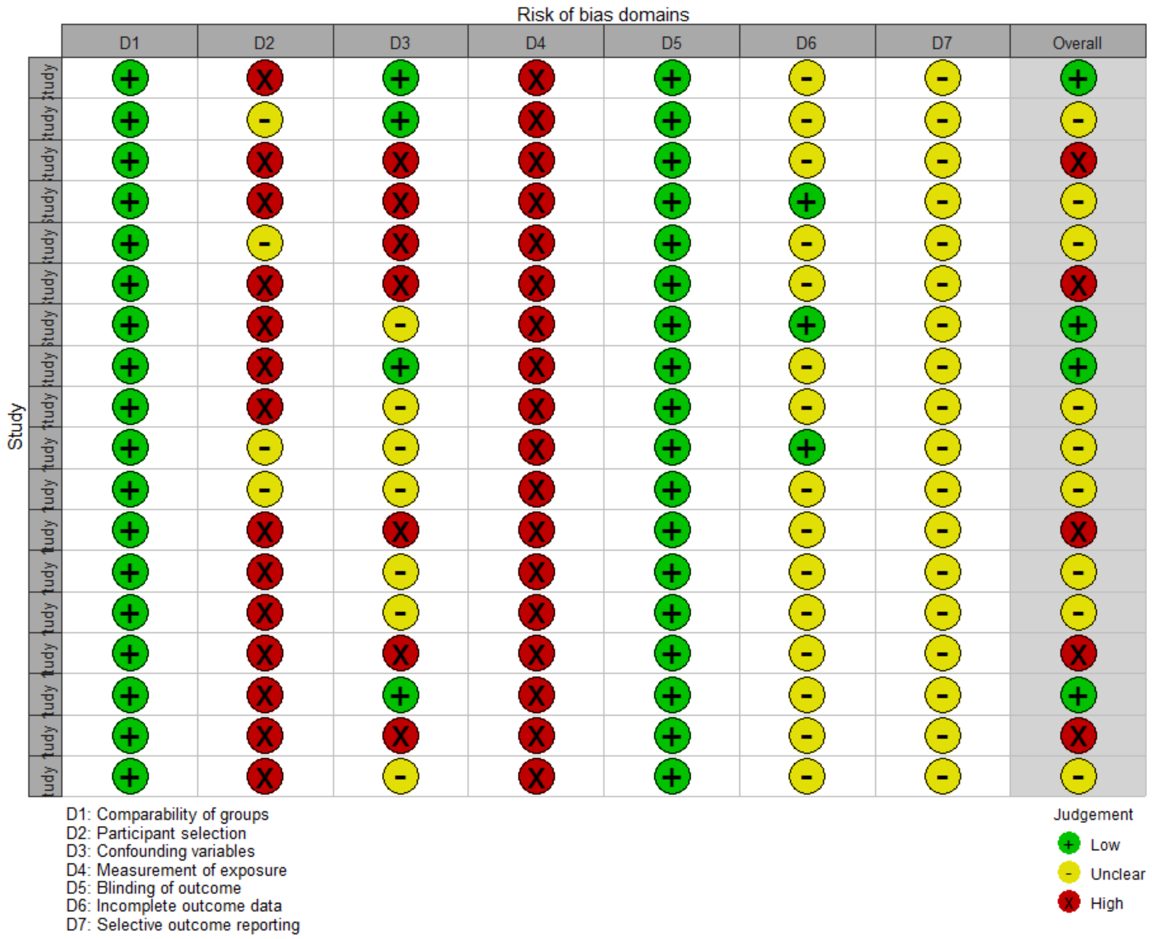
Risk of bias domains
D1
D2
D3
D4
D5
D6
D7
Overall
Study
D1: Comparability of groups
D2: Participant selection
D3: Confounding variables
D4: Measurement of exposure
D5: Blinding of outcome
D6: Incomplete outcome data
D7: Selective outcome reporting
Judgement
Low
Unclear
High

Supplemantary

Online Appendix : Search terms

Electronic Nicotine Delivery Systems
(((((((((((((((((((((((((((((((((((((((Electronic Nicotine Delivery System[Title/Abstract]) OR (Electronic Cigarettes[Title/Abstract])) OR (E-Cigs[Title/Abstract])) OR (E Cigs[Title/Abstract])) OR (E-Cig[Title/Abstract])) OR (E Cig[Title/Abstract])) OR (E-Cigarettes[Title/Abstract])) OR (E Cigarettes[Title/Abstract])) OR (E-Cigarette[Title/Abstract])) OR (E Cigarette[Title/Abstract])) OR (Electronic Cigarette[Title/Abstract])) OR (Cigarette, Electronic[Title/Abstract])) OR (Cigarettes, Electronic[Title/Abstract])) OR ("Electronic Nicotine Delivery Systems"[Mesh])) OR ("Vaping"[Mesh])) OR (E-Cig Use[Title/Abstract])) OR (E Cig Use[Title/Abstract])) OR (E-Cig Uses[Title/Abstract])) OR (Use, E-Cig[Title/Abstract])) OR (ECig Use[Title/Abstract])) OR (ECig Uses[Title/Abstract])) OR (Use, ECig[Title/Abstract])) OR (Vape[Title/Abstract])) OR (Vapes[Title/Abstract])) OR (E-Cigarette Use[Title/Abstract])) OR (E Cigarette Use[Title/Abstract])) OR (E-Cigarette Uses[Title/Abstract])) OR (Use, E-Cigarette[Title/Abstract])) OR (Nicotine Vaping[Title/Abstract])) OR (Nicotine Vapings[Title/Abstract])) OR (Vaping, Nicotine[Title/Abstract])) OR (Vapings, Nicotine[Title/Abstract])) OR (Ecigarette Use[Title/Abstract])) OR (Ecigarette Uses[Title/Abstract])) OR (Use, Ecigarette[Title/Abstract])) OR (Uses, Ecigarette[Title/Abstract])) OR (Electronic Cigarette Use[Title/Abstract])) OR (Cigarette Use, Electronic[Title/Abstract])) OR (Electronic Cigarette Uses[Title/Abstract])) OR (Use, Electronic Cigarette[Title/Abstract])
COVID-19
(((((((((((((((((((((((((((((((((((COVID 19[Title/Abstract]) OR (COVID-19 Virus

For peer review only

Disease[Title/Abstract])) OR (COVID 19 Virus Disease[Title/Abstract])) OR (COVID-19 Virus Diseases[Title/Abstract])) OR (Disease, COVID-19 Virus[Title/Abstract])) OR (Virus Disease, COVID-19[Title/Abstract])) OR (COVID-19 Virus Infection[Title/Abstract])) OR (COVID 19 Virus Infection[Title/Abstract])) OR (COVID-19 Virus Infections[Title/Abstract])) OR (Infection, COVID-19 Virus[Title/Abstract])) OR (Virus Infection, COVID-19[Title/Abstract])) OR (2019-nCoV Infection[Title/Abstract])) OR (2019 nCoV Infection[Title/Abstract])) OR (2019-nCoV Infections[Title/Abstract])) OR (Infection, 2019-nCoV[Title/Abstract])) OR (Coronavirus Disease-19[Title/Abstract])) OR (Coronavirus Disease 19[Title/Abstract])) OR (2019 Novel Coronavirus Disease[Title/Abstract])) OR (2019 Novel Coronavirus Infection[Title/Abstract])) OR (2019-nCoV Disease[Title/Abstract])) OR (2019 nCoV Disease[Title/Abstract])) OR (2019-nCoV Diseases[Title/Abstract])) OR (Disease, 2019-nCoV[Title/Abstract])) OR (COVID19[Title/Abstract])) OR (Coronavirus Disease 2019[Title/Abstract])) OR (Disease 2019, Coronavirus[Title/Abstract])) OR (SARS Coronavirus 2 Infection[Title/Abstract])) OR (Infection, SARS-CoV-2[Title/Abstract])) OR (SARS-CoV-2 Infection[Title/Abstract])) OR (SARS CoV 2 Infection[Title/Abstract])) OR (SARS-CoV-2 Infections[Title/Abstract])) OR (COVID-19 Pandemic[Title/Abstract])) OR (COVID 19 Pandemic[Title/Abstract])) OR (COVID-19 Pandemics[Title/Abstract])) OR (Pandemic, COVID-19[Title/Abstract])) OR ("COVID-19"[Mesh])
Tobacco, Tobacco Products, Tobacco Use
(((((Tobacco Uses[Title/Abstract]) OR (Tobacco Consumption[Title/Abstract])) OR (Consumption, Tobacco[Title/Abstract])) OR ("Tobacco Use"[Mesh])) OR ((((((((((((((Tobaccos[Title/Abstract]) OR (Nicotiana[Title/Abstract])) OR (Nicotianas[Title/Abstract])) OR (Nicotiana tabacum[Title/Abstract])) OR (Nicotiana tabacums[Title/Abstract])) OR (tabacum, Nicotiana[Title/Abstract])) OR

("Tobacco"[Mesh])) OR ("Smoking"[Mesh])) OR (Smoking Behaviors[Title/Abstract])) OR (Behavior, Smoking[Title/Abstract])) OR (Behaviors, Smoking[Title/Abstract])) OR (Smoking Behavior[Title/Abstract])) OR (Smoking Habit[Title/Abstract])) OR (Habit, Smoking[Title/Abstract])) OR (Habits, Smoking[Title/Abstract])) OR (Smoking Habits[Title/Abstract]))) OR ((((((((((((((((((((((((Product, Tobacco[Title/Abstract]) OR (Products, Tobacco[Title/Abstract])) OR (Tobacco Product[Title/Abstract])) OR (Cigarillos[Title/Abstract])) OR (Cigarillo[Title/Abstract])) OR (Pipe Tobacco[Title/Abstract])) OR (Pipe Tobaccos[Title/Abstract])) OR (Tobacco, Pipe[Title/Abstract])) OR (Cigars[Title/Abstract])) OR (Tobaccos, Pipe[Title/Abstract])) OR (Cigar[Title/Abstract])) OR (Kreteks[Title/Abstract])) OR (Kretek[Title/Abstract])) OR (Kreteks Tobacco[Title/Abstract])) OR (Kreteks Tobaccos[Title/Abstract])) OR (Tobacco, Kreteks[Title/Abstract])) OR (Tobaccos, Kreteks[Title/Abstract])) OR (Bidis[Title/Abstract])) OR (Bidi[Title/Abstract])) OR (Bidis Tobacco[Title/Abstract])) OR (Bidis Tobaccos[Title/Abstract])) OR (Tobacco, Bidis[Title/Abstract])) OR (Cigarettes[Title/Abstract])) OR (Cigarette[Title/Abstract])) OR ("Tobacco Products"[Mesh]))
Psychology
(("Psychology"[Mesh]) OR ("Psychology/diagnosis"[Mesh] OR "Psychology/psychology"[Mesh])) OR ((((((((((((((Side Effects, Psychological[Title/Abstract]) OR (Psychological Side Effect[Title/Abstract])) OR (Side Effect, Psychological[Title/Abstract])) OR (Psychological Side Effects[Title/Abstract])) OR (Psychosocial Factors[Title/Abstract])) OR (Factor, Psychosocial[Title/Abstract])) OR (Factors, Psychosocial[Title/Abstract])) OR (Psychosocial Factor[Title/Abstract])) OR (Psychological Factors[Title/Abstract])) OR (Factor, Psychological[Title/Abstract])) OR (Psychological Factor[Title/Abstract])) OR (Factors, Psychological[Title/Abstract])) OR

For peer review only

(Psychologist[Title/Abstract])) OR (Psychologists[Title/Abstract]))
Anxiety
(((((((Angst[Title/Abstract]) OR (Nervousness[Title/Abstract])) OR (Hypervigilance[Title/Abstract])) OR (Anxiousness[Title/Abstract])) OR (Social Anxiety[Title/Abstract])) OR (Anxieties, Social[Title/Abstract])) OR (Anxiety, Social[Title/Abstract])) OR (Social Anxieties[Title/Abstract])) OR (("Anxiety"[Mesh]) OR ("Anxiety/analysis"[Mesh] OR "Anxiety/diagnosis"[Mesh] OR "Anxiety/psychology"[Mesh]))
Stress, Psychological
((((((((((((Psychological Stresses[Title/Abstract]) OR (Stresses, Psychological[Title/Abstract])) OR (Life Stress[Title/Abstract])) OR (Life Stresses[Title/Abstract])) OR (Stress, Life[Title/Abstract])) OR (Stresses, Life[Title/Abstract])) OR (Stress, Psychologic[Title/Abstract])) OR (Psychologic Stress[Title/Abstract])) OR (Stressor, Psychological[Title/Abstract])) OR (Psychological Stressor[Title/Abstract])) OR (Psychological Stressors[Title/Abstract])) OR (Stressors, Psychological[Title/Abstract])) OR (Psychological Stress[Title/Abstract])) OR (("Stress, Psychological"[Mesh]) OR ("Stress, Psychological/analysis"[Mesh] OR "Stress, Psychological/diagnosis"[Mesh] OR "Stress, Psychological/psychology"[Mesh]))
Depression
(("Depression"[Mesh]) OR ("Depression/analysis"[Mesh] OR "Depression/diagnosis"[Mesh] OR "Depression/psychology"[Mesh])) OR (((((((((Depressions[Title/Abstract]) OR (Depressive Symptoms[Title/Abstract])) OR (Depressive Symptom[Title/Abstract])) OR (Symptom, Depressive[Title/Abstract])) OR (Symptoms, Depressive[Title/Abstract])) OR (Emotional Depression[Title/Abstract])) OR (Depression, Emotional[Title/Abstract])) OR (Depressions, Emotional[Title/Abstract])) OR

For peer review only

(Emotional Depressions[Title/Abstract]))

For peer review only

PRISMA 2020 Checklist

Section and Topic	Item #	Checklist item	Location where item is reported
**TITLE**			
Title	1	Identify the report as a systematic review.	1
**ABSTRACT**			
Abstract	2	See the PRISMA 2020 for Abstracts checklist.	2
**INTRODUCTION**			
Rationale	3	Describe the rationale for the review in the context of existing knowledge.	4
Objectives	4	Provide an explicit statement of the objective(s) or question(s) the review addresses.	5
**METHODS**			
Eligibility criteria	5	Specify the inclusion and exclusion criteria for the review and how studies were grouped for the syntheses.	6-7
Information sources	6	Specify all databases, registers, websites, organisations, reference lists and other sources searched or consulted to identify studies. Specify the date when each source was last searched or consulted.	6-7
Search strategy	7	Present the full search strategies for all databases, registers and websites, including any filters and limits used.	6
Selection process	8	Specify the methods used to decide whether a study met the inclusion criteria of the review, including how many reviewers screened each record and each report retrieved, whether they worked independently, and if applicable, details of automation tools used in the process.	6-7
Data collection process	9	Specify the methods used to collect data from reports, including how many reviewers collected data from each report, whether they worked independently, any processes for obtaining or confirming data from study investigators, and if applicable, details of automation tools used in the process.	6-7
Data items	10a	List and define all outcomes for which data were sought. Specify whether all results that were compatible with each outcome domain in each study were sought (e.g. for all measures, time points, analyses), and if not, the methods used to decide which results to collect.	6-7
	10b	List and define all other variables for which data were sought (e.g. participant and intervention characteristics, funding sources). Describe any assumptions made about any missing or unclear information.	6-7
Study risk of bias assessment	11	Specify the methods used to assess risk of bias in the included studies, including details of the tool(s) used, how many reviewers assessed each study and whether they worked independently, and if applicable, details of automation tools used in the process.	6-7
Effect measures	12	Specify for each outcome the effect measure(s) (e.g. risk ratio, mean difference) used in the synthesis or presentation of results.	7-8
Synthesis methods	13a	Describe the processes used to decide which studies were eligible for each synthesis (e.g. tabulating the study intervention characteristics and comparing against the planned groups for each synthesis (item #5)).	6-7
	13b	Describe any methods required to prepare the data for presentation or synthesis, such as handling of missing summary statistics, or data conversions.	6-8
	13c	Describe any methods used to tabulate or visually display results of individual studies and syntheses.	6-8
	13d	Describe any methods used to synthesize results and provide a rationale for the choice(s). If meta-analysis was performed, describe the model(s), method(s) to identify the presence and extent of statistical heterogeneity, and software package(s) used.	X
	13e	Describe any methods used to explore possible causes of heterogeneity among study results (e.g. subgroup analysis, meta-regression).	X
	13f	Describe any sensitivity analyses conducted to assess robustness of the synthesized results.	X
Reporting bias assessment	14	Describe any methods used to assess risk of bias due to missing results in a synthesis (arising from reporting biases).	6-8
Certainty	15	Describe any methods used to assess certainty (or confidence) in the body of evidence for an outcome.	X

For peer review only - http://bmjopen.bmj.com/site/about/guidelines.xhtml

PRISMA 2020 Checklist

Section and Topic	Item #	Checklist item	Location where item is reported
assessment			
**RESULTS**			
Study selection	16a	Describe the results of the search and selection process, from the number of records identified in the search to the number of studies included in the review, ideally using a flow diagram.	8
	16b	Cite studies that might appear to meet the inclusion criteria, but which were excluded, and explain why they were excluded.	8
Study characteristics	17	Cite each included study and present its characteristics.	9
Risk of bias in studies	18	Present assessments of risk of bias for each included study.	8
Results of individual studies	19	For all outcomes, present, for each study: (a) summary statistics for each group (where appropriate) and (b) an effect estimate and its precision (e.g. confidence/credible interval), ideally using structured tables or plots.	9-15
Results of syntheses	20a	For each synthesis, briefly summarise the characteristics and risk of bias among contributing studies.	9-15
	20b	Present results of all statistical syntheses conducted. If meta-analysis was done, present for each the summary estimate and its precision (e.g. confidence/credible interval) and measures of statistical heterogeneity. If comparing groups, describe the direction of the effect.	X
	20c	Present results of all investigations of possible causes of heterogeneity among study results.	X
	20d	Present results of all sensitivity analyses conducted to assess the robustness of the synthesized results.	X
Reporting biases	21	Present assessments of risk of bias due to missing results (arising from reporting biases) for each synthesis assessed.	7-8
Certainty of evidence	22	Present assessments of certainty (or confidence) in the body of evidence for each outcome assessed.	X
**DISCUSSION**			
Discussion	23a	Provide a general interpretation of the results in the context of other evidence.	16
	23b	Discuss any limitations of the evidence included in the review.	17-18
	23c	Discuss any limitations of the review processes used.	17-18
	23d	Discuss implications of the results for practice, policy, and future research.	18-19
**OTHER INFORMATION**			
Registration and protocol	24a	Provide registration information for the review, including register name and registration number, or state that the review was not registered.	X
	24b	Indicate where the review protocol can be accessed, or state that a protocol was not prepared.	X
	24c	Describe and explain any amendments to information provided at registration or in the protocol.	X
Support	25	Describe sources of financial or non-financial support for the review, and the role of the funders or sponsors in the review.	19
Competing interests	26	Declare any competing interests of review authors.	19
Availability of data, code and other materials	27	Report which of the following are publicly available and where they can be found: template data collection forms; data extracted from included studies; data used for all analyses; analytic code; any other materials used in the review.	6, Supplemantary part

For peer review only - http://bmjopen.bmj.com/site/about/guidelines.xhtml

From: Page MJ, McKenzie JE, Bossuyt PM, Boutron I, Hoffmann TC, Mulrow CD, et al. The PRISMA 2020 statement: an updated guideline for reporting systematic reviews. BMJ 2021;372:n71. doi: 10.1136/bmj.n71

PRISMA 2020 Checklist

For more information, visit: http://www.prisma-statement.org/

For peer review only

BMJ Open

A systematic review of changed smoking behavior, smoking cessation, and psychological states of smokers according to cigarette type during the COVID-19 pandemic

Journal:	*BMJ Open*
Manuscript ID	bmjopen-2021-055179.R1
Article Type:	Original research
Date Submitted by the Author:	27-Jan-2022
Complete List of Authors:	CHUN, HAE-RYOUNG; Seoul National University,<br>Cheon, Eunsil; Seoul National University, Graduate school of public health<br>Hwang, Ji-eun; Dankook University
**Primary Subject Heading**:	Smoking and tobacco
Secondary Subject Heading:	Epidemiology, Public health, Smoking and tobacco
Keywords:	COVID-19, PUBLIC HEALTH, MENTAL HEALTH

SCHOLARONE™
Manuscripts

I, the Submitting Author has the right to grant and does grant on behalf of all authors of the Work (as defined in the below author licence), an exclusive licence and/or a non-exclusive licence for contributions from authors who are: i) UK Crown employees; ii) where BMJ has agreed a CC-BY licence shall apply, and/or iii) in accordance with the terms applicable for US Federal Government officers or employees acting as part of their official duties; on a worldwide, perpetual, irrevocable, royalty-free basis to BMJ Publishing Group Ltd ("BMJ") its licensees and where the relevant Journal is co-owned by BMJ to the co-owners of the Journal, to publish the Work in this journal and any other BMJ products and to exploit all rights, as set out in our licence.

The Submitting Author accepts and understands that any supply made under these terms is made by BMJ to the Submitting Author unless you are acting as an employee on behalf of your employer or a postgraduate student of an affiliated institution which is paying any applicable article publishing charge ("APC") for Open Access articles. Where the Submitting Author wishes to make the Work available on an Open Access basis (and intends to pay the relevant APC), the terms of reuse of such Open Access shall be governed by a Creative Commons licence – details of these licences and which Creative Commons licence will apply to this Work are set out in our licence referred to above.

Other than as permitted in any relevant BMJ Author's Self Archiving Policies, I confirm this Work has not been accepted for publication elsewhere, is not being considered for publication elsewhere and does not duplicate material already published. I confirm all authors consent to publication of this Work and authorise the granting of this licence.

For peer review only

A systematic review of changed smoking behavior, smoking cessation, and psychological states of smokers according to cigarette type during the COVID-19 pandemic

1 Chun, Hae-ryoung; Seoul National University, Graduate school of public health; mamimi777@snu.ac.kr

2 Cheon, Eunsil; Seoul National University, Graduate school of public health; silverberry2@snu.ac.kr

* Correspondence to: Jieun Hwang Dankook University College of Health Science, 119, Dandae-ro, Dongnam-gu, Cheonan-si, Chungcheongnam-do, Republic of Korea loshjeve@gmail.com

For peer review only

For peer review only - http://bmjopen.bmj.com/site/about/guidelines.xhtml

ABSTRACT

Objectives: Although the global coronavirus disease 2019 (COVID-19) pandemic has increased interest in research involving high-risk smokers, studies examining changed smoking behaviors, cessation intentions, and associated psychological states among smokers are still scarce. This study aimed to systematically review the literature related to this subject.

Design: A systematic review of published articles on cigarettes and COVID-19-related topics

Data Sources: Our search was conducted in January 2021. We used the keywords COVID-19, cigarettes, electronic cigarettes (e-cigarettes), and psychological factors in PubMed and ScienceDirect and found papers published between January and December 2020.

Data selection: We included articles in full text, written in English, and that surveyed adults. The topics included smoking behavior, smoking cessation, psychological state of smokers, and COVID-19-related topics.

Data extraction and synthesis: Papers of low quality, based on quality assessment, were excluded. Thirteen papers were related to smoking behavior, nine papers were related to smoking cessation, and four papers were related to psychological states of smokers.

Results: Due to the COVID-19 lockdown, there was a habit of purchasing large quantities of cigarettes in advance. Additionally, smokers who only smoked cigarettes had increased their attempts and willingness to quit smoking, compared to e-cigarette users.

Conclusions: Due to the COVID-19 outbreak, the intention to quit smoking was different among smokers, according to cigarette type (cigarette only, e-cigarette, and dual users). As the COVID-19 pandemic continues, policies and campaigns that could increase smoking cessation intentions and attempts to quit smoking among smokers who are at high-risk of COVID-19 should be put in place. Additionally, for e-cigarette users with poor health-seeking behavior, interventions to increase the intention to quit smoking are needed.

Keyword: COVID-19 Pandemic, Quitting Smoking, Smoking Habits

STRENGTHS AND LIMITATIONS OF THIS STUDY

- This study is the first to systematically review the literature regarding changes in smoking behaviors, cessation intentions, and psychological states of smokers, based on study period, country, and age of participants during the coronavirus disease 2019 pandemic.
- This study could serve as primary data for establishing effective strategies that focus on specific tobacco product users by suggesting different smoking behaviors, smoking cessation intentions, and psychological changes, based on cigarette type.
- We did not investigate changes in adolescent smoking behavior, cessation intention, and psychological states because this study systematically reviewed literature targeting

For peer review only

adults.

- Since the articles collected for this systematic review were only cross-sectional designs, it was difficult to determine causality; therefore, only associations were presented.

Since our purpose was to investigate only the smoking situation at the beginning of the COVID-19 pandemic, we systematically reviewed only the articles published between January and December 2020.

For peer review only

For peer review only - http://bmjopen.bmj.com/site/about/guidelines.xhtml

1. INTRODUCTION

On January 30, 2020, the World Health Organization (WHO) declared coronavirus disease 2019 (COVID-19), an infectious disease of the respiratory tract characterized by severe acute respiratory syndrome,[1] a public health emergency of international concern (PHEIC).[2] In 2020, a total of 82,357,480 cases of COVID-19 were reported, including 1,802,390 deaths (https://covid19.who.int/). Due to the pandemic, different standards and steps to control the spread of the disease have been adopted around the world, but lockdown and social distancing measures to prevent COVID-19 infection have been implemented by almost all countries.

Smoking is a high-risk factor for COVID-19 infection and there are many systematic reviews and meta-analyses that report the association between COVID-19 and smoking.[3-5] Since tobacco is associated with chronic lung disease, physicians could expect more smokers to develop the severe form of COVID-19,[3] given the effect of tobacco on respiratory disease and immune function.[6] Smoking is associated with negative progression and side effects of COVID-19.[4] Smoking, including current or previous smoking history, significantly increases the severity and risk of death from COVID-19.[5] Previous studies have suggested a number of public health messages to continue focusing on smoking cessation efforts; however, smoking rates have not decreased.[7]

A few investigations have been conducted on whether smoking behavior[8] and cessation efforts[9] have changed during the COVID-19 pandemic. Previous studies have shown that smoking has decreased[8] and the number of quitting attempts has increased[9] since the COVID-19 pandemic. Since lockdowns and various social distancing measures were implemented in the early stages of the COVID-19 pandemic, they have the potential to affect smoking behavior and cessation intention. In some countries, it is expected that smoking behavior will change given the difficulty in freely purchasing tobacco products as non-essential items; individuals are locked down under the pandemic and are required to stay at home for prolonged periods.[10] Changes in daily life due to social distancing and lockdowns are expected to help reduce smoking consumption.[11] Therefore, it is necessary to examine the factors influencing smoking behavior and cessation intention in the early stages of the pandemic, which have been investigated in the literature.

There are currently insufficient studies on smokers' negative psychological states during the COVID-19 pandemic. Further research is needed because the relationship between negative psychological states and health behavior is unclear.[12] People experience higher levels of stress than usual with social isolation, employment, finances, responsibilities, and concerns about becoming ill from the virus.[13] Therefore, many smokers smoke to relieve this stress and these negative emotions,[14] but it is often counterproductive.[15] Previous research has shown that negative psychological states, such as stress, anxiety, and depression can influence tobacco use behavior.[16]

There are also insufficient studies to compare the smoking behavior, cessation intention, and psychological state of cigarette and e-cigarette users during the COVID-19 pandemic.[10] The daily use of e-cigarettes among e-cigarette smokers slightly increased during the COVID-19 lockdown;[17] therefore, it is necessary to investigate whether smoking behavior changes

For peer review only - http://bmjopen.bmj.com/site/about/guidelines.xhtml

depending on the cigarette type. Cigarette users are aware that they engage in harmful health behaviors. On the other hand, it is unclear whether e-cigarettes help smokers quit smoking.[18] E-cigarette users believe e-cigarettes are less harmful to health as they contain relatively less harmful ingredients than cigarettes.[19] They believe that smoking e-cigarettes are in itself an effort to quit smoking.[19]

Many studies have investigated whether smoking is a high-risk factor for COVID-19.[4, 5] However, relatively few studies have been published on whether smoking behavior, cessation intention, and psychological state have changed during the COVID-19 pandemic. In the future, it is expected that this study will establish a scientific basis and policy response to smoking behavior, cessation intention, and psychological state based on cigarette type, both during and post COVID-19. Therefore, we conducted a systematic review based on the hypothesis that there would be changes in smoking behavior, cessation intention, and psychological states of adult smokers, depending on the tobacco product used during the COVID-19 pandemic.

For peer review only - http://bmjopen.bmj.com/site/about/guidelines.xhtml

2. METHODS

2.1. Literature Search Strategy

We systematically reviewed the data according to the Preferred Reporting Items for Systematic Reviews and Meta-Analyses (PRISMA), shown in the Supplementary file. We searched articles using MeSH terms 'COVID-19, depression, stress, psychological, anxiety, tobacco, tobacco products, tobacco use, and electronic nicotine delivery system' in January 2021. Only papers published between January and December 2020 were included in the study. All search terms are listed in Supplementary Table 1.

A literature search was conducted on PubMed using the following MeSH terms: "(depression OR stress OR anxiety OR psychology) AND ((tobacco OR tobacco products OR tobacco use OR electronic nicotine delivery system) AND COVID-19))" in the title or the abstract in 2020. A literature search was conducted on ScienceDirect using the following search terms: "COVID-19 AND (e-cigarette OR "electronic cigarette" OR "electronic nicotine delivery" OR "vaping" OR "heat not burn" OR "heated tobacco product")," as well as COVID-19 AND (tobaccos OR cigarette OR kretek OR bidis OR "pipe tobacco" OR cigarillos).

2.2. Inclusion and Exclusion Criteria

Figure 1 shows a flowchart of the database searches, followed by the exclusion/inclusion strategy. The inclusion criteria were cases that did not meet the exclusion criteria. In the first phase (Figure 1), certain types of articles were excluded, including comments, letters, editorials, viewpoints, correspondence, and articles without full text. In addition, duplicate works and those not written in English, as well as studies that did not include humans or adults, were excluded. In the next phase (Figure 1), studies that were not related to tobacco or COVID-19 were excluded. In addition, articles were excluded if the topics were not related to changes in tobacco use and mental health caused by COVID-19. Finally, articles with a high risk of bias were excluded using the Risk of Bias Assessment Tool for Non-randomized Studies (RoBANS) (Figures 2 and 3).[20]

2.3. Data Extraction

Two independent reviewers (HR and JE) screened the literature and assessed each paper by reading the titles, abstracts, and full texts. Data from studies that fulfilled our inclusion criteria were extracted independently by three authors (HR, JE, and ES). The main data points included were the following: study details (author, journal, publication date, country, study design, study period, and funding), total number of participants, types of cigarettes, changes in smoking behavior, cessation intention, and psychological state.

Two authors (HR and ES) independently assessed the quality of included studies using the RoBANS.[20] If the opinions of the two authors were different, the quality evaluation was completed through further discussion with another researcher (JE). The RoBANS contains six

For peer review only

domains: selection of participants, confounding variables, measurement of exposure, blinding of outcome assessments, incomplete outcome data, and selective outcome reporting (Figures 2 and 3). The level of risk of bias was divided into three stages (high, unclear, or low risk). When the risk of bias in a study was high, it was excluded.

Using the backward snowballing method,[21, 22] we checked the references of the articles that met the inclusion criteria. We checked the titles, journals, abstracts, and full texts of the references of the articles. We excluded articles that did not meet the inclusion criteria. In addition, articles not related to the subject of this study were excluded. As a result, no additional papers were added.

3. RESULTS

3.1. **Literature Search and Literature Selection Results**

A total of 1,659 papers were found in PubMed and ScienceDirect, and 905 non-original papers were excluded. Eleven papers without full text and four papers not written in English were excluded. In addition, 413 papers were selected, excluding 102 papers that were not conducted in humans, 114 papers that were not conducted in adults, and 110 duplicate papers after the first screening phase. Papers unrelated to tobacco (n = 200) and COVID-19 (n = 21) were excluded. In addition, a total of 18 papers were included, excluding 166 papers that were not related to tobacco behavior and psychological states that changed during the COVID-19 pandemic, as well as eight papers targeting COVID-19 patients or medical personnel in the first screening phase.

3.2. **Assessment result of the risk of bias**

A total of 13 papers were selected after excluding five papers with a high risk of bias, as determined through quality evaluation of the 18 papers using the RoBANS(Figures 2 and 3).

3.3. Characteristics of included papers by three subjects

All 13 selected papers were published in 2020; three papers were conducted in the US, three in the UK, two in Italy, two in Turkey, and one each in China, India, and Australia. All were cross-sectional design studies that used surveys. Comments, letters, editorials, viewpoints, and correspondence were not included. In the included papers, the number of study participants varied from 93 to 52,002 and all participants were adults. We also presented the average age of the participants in the papers or the ratio by age group. Eight papers only targeted cigarette smokers and five papers reviewed e-cigarette and cigarette users together (Table 1). Table 1 shows that smokers' smoking behavior, cessation intentions, and psychological states changed due to the COVID-19 pandemic, presented in all 13 papers. Seven papers studied changes in smoking behavior, eight papers examined changes in smoking cessation, and five papers

For peer review only - http://bmjopen.bmj.com/site/about/guidelines.xhtml

examined changes in smokers' psychological state after the COVID-19 outbreak (Table 1).

3.4. Integrating results of the 13 selected papers

In the early stages of the COVID-19 pandemic, behavioral changes did not appear to be the same, such as increasing or decreasing the amount of cigarettes smoked, regardless of cigarette type. However, smoking cessation intentions and attempts were higher among cigarette users than e-cigarette users. In addition, if the average age of the study participants was ≥40 years, the number of attempts to quit smoking was higher. In the case of those in their 30s, the higher the awareness of COVID-19 infection, the higher the attempts to quit smoking. In addition, it was found that all smokers, regardless of the tobacco product used, had increased stress and anxiety during the COVID-19 pandemic, and the more they did not intend to quit, the more stressed they were. We present detailed information on smoking behavior, smoking cessation, and psychological state changes according to cigarette type during the COVID-19 pandemic in the six sub-headings that follow.

3.4.1. Changes in smoking behavior in e-cigarette users (or dual users) during the COVID-19 outbreak

According to Soule et al.,[23] the higher the reliance on e-cigarettes, the higher the incidence of cigarette use. There have also been increasing efforts to reduce e-cigarette use among dual users. However, mastery of e-cigarette-specific behaviors during the additional time spent at home improved e-cigarette skills and increased e-cigarette use.[23] According to the article by P. Caponnetto et al.,[24] daily cigarette consumption by dual users of cigarettes and e-cigarettes and cigarette users decreased slightly. Due to the COVID-19 outbreak, the method of purchasing products, including tobacco products, has changed; individuals buy large numbers of products at once.

3.4.2. Changes in smoking behavior in cigarette users during the COVID-19 outbreak

According to the results of articles by Cancello et al.[7] and Ren et al.,[25] tobacco consumption increased by 38% and 30%, respectively. Compared to before the COVID-19 pandemic, Chopra et al. showed a significant decrease in smoking consumption (0.02 [0.03], $P < 0.05$).[8] However, in the study by Ozcelik et al.,[26] 67.5% of participants reported no change in smoking behavior, which cannot be interpreted as a significant change in smoking behavior during the COVID-19 pandemic.

3.4.3. Changes in intentions and attempts to quit smoking among e-cigarette users (or dual users) during the COVID-19 outbreak

Tattan-Birch et al.[9] found that only a few smokers attempted to quit smoking due to the COVID-

For peer review only - http://bmjopen.bmj.com/site/about/guidelines.xhtml

19 outbreak. Approximately one in 10 current e-cigarette users reported an attempt to quit vaping because of COVID-19. Soule et al. reported that users with high reliance on e-cigarettes exerted less effort to reduce product usage than users with low reliance. According to a study by Caponnetto et al.,[10] while most exclusive cigarette smokers have considered quitting smoking, most exclusive e-cigarette users have not considered stopping e-cigarette use. According to a study by Chertok et al.,[27] 36.7% of all smokers attempted to quit smoking during the pandemic regardless of cigarette type, people who quit smoking due to COVID-19 perceived smoking as a high-risk factor for COVID-19 infection. In a study conducted in the US, all e-cigarette users had lower intentions and attempts to quit smoking.[23]

3.4.4. Changes in intentions and attempts to quit smoking among cigarette users during the COVID-19 outbreak

According to Jackson et al.,[28] COVID-19 shutdown has been associated with increased attempts to quit smoking. According to the results of the article by Tetik et al.,[29] which compared the success rates of smoking cessation before and during the pandemic, the COVID-19 outbreak effectively promoted smoking cessation. Only 12.8% of people who quit smoking during the COVID-19 outbreak did not restart smoking. According to Ozcelik et al.,[26] people with coronaphobia exhibited a significantly higher decrease or cessation in smoking than those with no change in smoking behavior or increase smoking consumption. In the US, a study of cigarette smokers found that the number of attempts to quit smoking had increased.[27,30] In the UK, all studies conducted on cigarette users found that attempts to quit smoking had increased.[9,31] In the case of cigarette users, there are more successful cases of quitting smoking compared to e-cigarette users. Smoking cessation attempts were higher among those ≥40 years. In the case of people in their 30s, the higher their awareness of the COVID-19 infection, the higher their attempts to quit smoking, and the less reliant they were on cigarettes, the higher their attempts to quit smoking.

3.4.5. The association between smoking and psychological changes in e-cigarette users (or dual users) during the COVID-19 outbreak

According to Soule et al.,[23] the greater the dependence on e-cigarettes, the higher the concern about COVID-19 infection than those with low dependence. In the article by S.D. Kowitt et al.,[30] the higher the awareness regarding the risk of contracting COVID-19, regardless of cigarette type, the greater the willingness to quit ($B = 0.38$, $p < 0.001$) and the attempt to quit smoking (OR: 1.31, 95% CI: 1.04–1.64). In addition, as social distancing ($B = 0.11$, $p = 0.01$) was enforced more strongly, the willingness to quit smoking increased. For both cigarette users and e-cigarette users, smokers are more stressed than non-smokers, and the more they have no intention of quitting, the more stressed they are.

3.4.6. The association between smoking and psychological changes in e-cigarette users (or

For peer review only - http://bmjopen.bmj.com/site/about/guidelines.xhtml

dual users) during the COVID-19 outbreak

Stanton et al.[32] found that people with increased tobacco consumption had a higher risk of depression, anxiety, and stress. Jackson et al.[33] reported that compared to non-smokers, current and former smokers had increased stress from COVID-19. Ozcelik et al.[26] indicated that among people with coronaphobia, those who reduced or stopped smoking felt more anxious than those who increased their smoking frequency.

For peer review only

For peer review only - http://bmjopen.bmj.com/site/about/guidelines.xhtml

Table 1 Characteristics of included papers by three subjects

First Author, Setting Period	Age	Type of cigarette(N,%)	Subject 1* Smoking behavior	Subject 2* Smoking cessation	Subject 3* Psychological states
H. Tattan-Birch[9]<br>England<br>April-May	Mean(SD)<br>52.4 (17.6)	Total : 3,179<br>-Smoking cigarette never:1,804(56.7%)<br>long term ex smokers: 834 (26.2%)<br>recent ex smokers: 72 (2.3%)<br>current smoker : 469 (14.8%)<br>- E-cigarettes only<br>no current use : 2,987 (94%)<br>current use : 192 (6%)		Quit Attempt<br>- Cigarette smoker: 12.2%<br>- E-cig: 10%	
E.K. Soule[23]<br>US<br>April	Mean(SD)<br>35.1(10.8)	-Total : E-cig use (n=93)<br>- Dual user of E-cig and the following products<br>1) Cigarettes : 50<br>2) Cigar : 11<br>3) Cigarillo or little cigar : 19<br>4) Smokeless : 7<br>5) Waterpipe : 11	1. Efforts to reduce the use of e-cigs have increased for dual users<br>(M = 3.71 vs. M = 3.02; p < 0.05),<br>but e-cig use skills have increased<br>(M = 3.48 vs. M = 2.27; p < 0.05)<br>and usage has increased<br>(M = 4.34 vs. M = 3.72;p < 0.05).<br>2. Users with high dependence on e-cigs have increased use of e-cigs compared to users with low dependence.<br>(M = 4.62 vs. M = 3.35; p < 0.001)	Users who depended more on e-cigs had less effort to reduce their use of e-cigs<br>(M = 3.10, SD = 0.63)<br>than users who were less dependent on e-cigs<br>(M = 3.79, SD = 0.61; p < 0.02).	Users who rely heavily on e-cigs are more concerned about COVID-19 than users who do not rely heavily on e-cigs<br>(M = 3.86 vs. M = 3.06; <0.05).
Chertok[27]<br>US Ohio<br>April	Mean(SD)<br>33.5 (14.6)	Total : 810<br>1. Cigarettes only: 115 (62.8%)<br>2. Both cigarettes and e-cig: 68(37.2%)<br>3. Not smoker: 627		(Not classified according to the type of cigarette, but surveyed for all smokers.)<br>- Attempt to quit : 36.7% (n = 66)<br>- Those who quit since COVID-19, those who desired to quit, perceived higher risk of infection.	

For peer review only

For peer review only - http://bmjopen.bmj.com/site/about/guidelines.xhtml

First Author,<br>Setting Period	Age	Type of cigarette(N,%)	Subject 1*<br>Smoking behavior	Subject 2*<br>Smoking cessation	Subject 3*<br>Psychological states
P.Caponnetto[10]<br>Italy<br>2nd-26th April	Mean(SD)<br>34.7 (14.11)	Total : 1,825<br>Dual user of<br>Cigarettes and E- Cigarettes(64,3.5%)<br>cigarette and HTPs (33,1.8%),<br>Exclusive cigarette smoker(582, 32%)<br>Exclusive user of HTPs(81,4.4%)<br>Exclusive user of e-cig (225, 12.3%)<br>Former smoker(293, 16%)<br>Never smoker(547, 30%)	1. Dual users of cigarette and e-cig and exclusive cigarette smokers'(sr value (-2.7 and -1.9)) daily consumption has decreased.<br>2. "Exclusive cigarette users" and the "Exclusive Ecig users" changed the way of purchasing products and the majority of them changed their habits by having large stocks of cigarettes or e-liquids/ cartridges at home to avoid going out every day.	Most exclusive cigarette smokers(sr=11.6) have considered quitting but most exclusive e-cig users(sr=-4.2) have not considered stopping the use of e-cigs.	
S.D. Kowitt[30]<br>US<br>23rd April-7th May	Mean(SD)<br>39.9 (13.4)	Total: 777<br>Cigarette only 651 (83.8%)<br>E-cig only 293(37.3%)<br>Smokeless tobacco only 170(21.9%)<br>Waterpipe tobacco only 103(13.3%)			(Not classified according to the type of cigarette, but surveyed for all smokers.)<br>-Having higher risk perceptions of COVID-19 was associated with higher quit intentions (B = 0.38, p < 0.001)<br>-Higher odds of quit attempt since COVID-19 started (aOR: 1.31, 95% CI: 1.04, 1.64).<br>-Greater social distancing efforts were associated with higher quit intentions (B = 0.11, p = 0.01).
R. Stanton[32]<br>Australia<br>April	Mean(SD)<br>50.5 (14.9)	Total = 1,491<br>Cigarettes only=172(11.5%)			Those who reported a negative change in smoking, they were more likely to have higher depression (aOR = 1.09, 95% CI = 1.04, 1.13), anxiety (aOR = 1.12, 95% CI = 1.06, 1.18), and stress (aOR = 1.10, 95% CI = 1.05, 1.15).
Jackson[31]<br>England<br>21st March-20th April	18-29: 19.5%<br>30's :15.0%<br>40's: 17.7%<br>50's: 17.5%<br>60's: 19.1%<br>≥70: 11.2%	Total: 53,002<br>Ex-smokers(cigarette) :13,602(25.7%)<br>Current smokers (cigarette only):8,057(15.2%)			Current and ex-smokers had higher odds than never smokers of reporting significant stress about becoming seriously ill from COVID-19 (current: OR=1.34 (1.27–1.43); ex-smokers: OR=1.22 (1.16–1.28)).
Jackson[33]<br>England<br>March-April	18–24: 13.4%<br>25–34: 16.9%<br>35–44: 15.7%<br>45–54: 17.0%<br>55–64: 14.5%<br>≥ 65: 22.5%	Cigarettes only: 20,558	The COVID-19 lockdown was not associated with a significant change in smoking prevalence 17.0% (after) versus 15.9% (before),<br>OR = 1.09, 95% CI = 0.95–1.24.	associated with increases in quit attempts 39.6 vs 29.1%, aOR=1.56, 95%CI=1.23–1.98, quit success 21.3 vs 13.9%,<br>aOR = 2.01, 95% CI = 1.22–3.33<br>and cessation (8.8 vs 4.1%, aOR = 2.63, 95% CI = 1.69–4.09) among past-year smokers	
Kayhan Tetik[34]<br>Turkey May	Mean(SD)<br>39.5 (12.1)	Only Cigarette users: 357		165(46.2%) smokers of participants, 86 (24.1%) did not quit despite this pandemic.	

For peer review only

First Author, Setting Period	Age	Type of cigarette(N,%)	Subject 1* Smoking behavior	Subject 2* Smoking cessation	Subject 3* Psychological states
Ren[25] China 14th February -29th March	Mean: 22.0 (min-max: 21.0–37.0)	Only Cigarette users:1,172	Some (30.1%) increased smoking during the pandemic.		
Ozcelik[26] Turkey 1st January-30th June	Mean: 41.2 (min–max: 18–82)	Cigarette only Current smoker (N= 111, 98%) Quitted smoking(N= 3, 2%)	77 people (67.5%) had no behavioral change associated with smoking	People with coronaphobia exhibited a significantly higher decrease or cessation of smoking than no change in smoking behavior (p: .006, odds ratio: 18.222 (2.301–144.305).	Decrease or cessation of smoking in people with coronaphobia was found significantly greater in the individuals who had severe anxiety than who increased smoking (p: .36, odds ratio:10.667 [1.171–97.185]).
R. Cancello[7] Italy 15th April- 4th May	18-30:14.5% 31-60:65.1% ≥60: 20.4%	Total: 490 Cigarette only smoker: 105(21.4%)	Cigarette Consumption increase: 38%		
Chopra[8] India 15th August- 30th August	Mean(SD) 33.33(14.5)	No smoking: 939(94.4%) Only Cigarette user: 56	Smoking consumption (0.02 , P <0.05) is significantly reduced.		

*Subject 1 is a case in which a paper contains changes in smoking behavior before and during the COVID-19 pandemic. Subject 2 is a case in which a paper contains perceptions of smoking or cessation intention before and during the COVID-19 pandemic. Subject 3 is a case in which a paper contains information on changes in psychological states, such as depression, stress, and anxiety before and during the COVID-19 pandemic.

For peer review only

For peer review only - http://bmjopen.bmj.com/site/about/guidelines.xhtml

4. DISCUSSION

We examined the changes in smoking behavior, cessation intentions, and psychological states caused by the COVID-19 outbreak in smokers (cigarette users, e-cigarette users, and dual users). Regardless of the cigarette type, in some cases, the amount of smoking either increased or decreased. However, compared to e-cigarette users, tobacco users' intentions and attempts to cease smoking increased. In addition, regardless of the type of cigarette, smokers showed a negative psychological state due to COVID-19. It was found that attempts and intentions to cease smoking increased as dependence on cigarettes decreased. Additionally, social distancing strengthened and awareness about COVID-19 increased.

The amount of smoking done by e-cigarette-only users increased with increased dependency. For dual users, those who use cigarettes and e-cigarettes, efforts to reduce product usage increased; however, there was a tendency to buy cigarettes in bulk, increase e-cigarette usage skills, and increase their use. This is thought to be the result of a tendency to recognize that using e-cigarettes is relatively less harmful to health than smoking cigarettes.[19] According to previous studies, many e-cigarette smokers recognize that e-cigarettes are relatively less harmful to health, their own and others', [19] because they contain far fewer harmful components [35] than cigarettes. However, it is not yet clear to what extent e-cigarettes can help smokers quit smoking.[18] For this reason, e-cigarette users should consider the risks of using e-cigarettes as much as cigarettes.

Cigarette users' efforts to reduce product usage and attempts to quit smoking during the pandemic have increased, but efforts to reduce product usage by e-cigarette users have decreased. In particular, the higher the dependence of e-cigarette users, the higher the worries related to COVID-19; however, efforts to reduce the amount of smoking were lower than users with low e-cigarette dependence. The reason for this could be that e-cigarette users think that using e-cigarettes is an attempt to quit smoking.[19] Therefore, e-cigarette users' intentions to quit smoking have not increased, even during the pandemic. A cohort analysis conducted in the US found that e-cigarette users did not quit smoking, compared to smokers who did not use e-cigarettes, and more than half of e-cigarette users still used e-cigarettes a year later.[36] In another study, it was not clear whether e-cigarette users were more likely to quit smoking.[37] Since many studies are still being conducted on the cessation effect of e-cigarettes, it should be recognized that e-cigarette use could be harmful to health.

Regardless of the cigarette type, the higher the awareness of the risks associated with contracting COVID-19, the higher the level of social distancing, since the start of COVID-19, and intent to quit smoking. In the case of tobacco users, the presence of coronaphobia increased smoking cessation intentions. Among them, those who reduced smoking felt more anxious than those who increased their amount of smoking. Smokers were more likely to quit after understanding the health risks of smoking due to the "vulnerability hypothesis," wherein smokers who care about their health would be inclined to quit smoking when they know it makes them vulnerable to certain diseases.[38, 39] This presents the same context as the results of this systematic review.

In contrast, previous studies have shown that smokers have higher levels of depression, anxiety,

For peer review only - http://bmjopen.bmj.com/site/about/guidelines.xhtml

and stress, as well as increased smoking amounts. According to a study by Klemperer et al.,[40] 28.3% of patients reduced their tobacco use due to fear of infectious diseases, while 30.3% showed increased tobacco use, anxiety levels were high in both groups. However, in the literature surveyed in this study, it was found that the higher the level of anxiety, the higher the attempt to quit smoking. Therefore, further research exploring the relationship between mental health and smoking behaviors during the COVID-19 pandemic using standardized tools to measure mental health conditions, such as anxiety, is needed.

Increased awareness of COVID-19 risk has led to increased smoking cessation attempts, which can be considered a desirable outcome for physical health in the long run. However, for mental health, including anxiety, depression, and increased stress during the pandemic, governments' response to address the increasing trend of smoking should be to conduct campaigns for risk awareness to protect their citizens' mental health. Research on the association between cigarette smoking and COVID-19 is increasing; the WHO also suggests that smokers may be considered at high risk of contracting COVID-19. However, due to the lack of research on the relationship between the use of liquid e-cigarettes and COVID-19, the willingness or attempts to quit e-cigarette use has not increased compared to cigarette-only smokers or dual users. As smokers have a high risk of contracting certain diseases, both during and after the COVID-19 pandemic, further studies on the risk of e-cigarettes and their impact on smoking cessation, especially regarding COVID-19 infection, should be conducted.

5. STRENGTH AND LIMITATION

A limitation of this study is that only cross-sectional studies were included. Since the purpose of this study was to investigate people's smoking behavior, smoking cessation, and psychological state in the early stages of the COVID-19 pandemic in 2020, all studies used questionnaire tools. There were no articles that had conducted qualitative or interventional studies on this topic at the time. Second, this study systematically reviewed only articles published between January and December 2020. As the vaccine was introduced in 2021, different policies to respond to COVID-19 were implemented in 2020. As a result, smoking behavior, smoking cessation, and psychological state in 2021 will differ to 2020. When reviewing articles published in 2021, it is necessary to examine the changes in smoking behavior, smoking cessation, and psychological state of smokers while considering vaccine variables. However, it is meaningful that this study looked at smoking usage behavior during the COVID-19 pandemic based on cigarette type.

6. CONCLUSION

In this study, we examined changes in smoking behaviors, smoking cessation intentions, and psychological states of smokers during COVID-19 according to cigarette type. We conducted

For peer review only - http://bmjopen.bmj.com/site/about/guidelines.xhtml

a systematic review of 13 papers to suggest future research and policy directions that the government may implement for COVID-19 and smokers. During the COVID-19 pandemic, cigarette users' consumption did not change significantly, but dual users and e-cigarette users with a high dependence on e-cigarettes showed increased consumption. Cigarette smokers' quit intentions, attempts, and success rates of cessation increased, and even those with coronaphobia showed increased intent to quit smoking. On the other hand, users of e-cigarettes demonstrated fewer attempts to quit. As the COVID-19 pandemic continues, policies and campaigns should be implemented to increase the intent and attempt to stop smoking. Research and interventions on COVID-19, smoking, and e-cigarette use should also be conducted in the medium to long term.

CONTRIBUTIORS

HC designed the study, analyzed and interpreted the data, and wrote the manuscript. JH and EC contributed to the study design and interpretation of the results.

FUNDING

This study was supported by the National Research Foundation of Korea (NRF) grant, funded by the Ministry of Science and ICT (MSIT) (No. 2020R1C1C1012562).

COMPETING INTERESTS

None.

PATIENT CONSENT FOR PUBLICATION

Not required.

ETHICS APPROVAL

This systematic review was based on published data. As researchers did not access any information that could lead to the identification of an individual patient, no ethical issues were raised in this research. Therefore, obtaining ethical approval and consent from the participants was waived.

PROVENANCE AND PEER REVIEW

Not commissioned; externally peer reviewed.

DATA SHARING STATEMENT

The data set used and/or analyzed for this review will be available from the corresponding author upon reasonable request.

REFERENCES

1. Berlin I, Thomas D, Le Faou A-L, Cornuz J. COVID-19 and smoking. *Nicotine and Tobacco*

For peer review only - http://bmjopen.bmj.com/site/about/guidelines.xhtml

Research. 2020;22(9):1650-1652.

2. Organization WH. COVID 19 Public Health Emergency of International Concern (PHEIC). Global research and innovation forum: towards a research roadmap. 2020;

3. Ni Y, Shi G, Qu J. Indoor PM2. 5, tobacco smoking and chronic lung diseases: A narrative review. *Environmental research*. 2020;181:108910.

4. Vardavas CI, Nikitara K. COVID-19 and smoking: A systematic review of the evidence. *Tobacco induced diseases*. 2020;18

5. Umnuaypornlert A, Kanchanasurakit S, Lucero-Prisno DEI, Saokaew S. Smoking and risk of negative outcomes among COVID-19 patients: A systematic review and meta-analysis. *Tobacco induced diseases*. 2021;19

6. Health UDo, Services H. The health consequences of smoking—50 years of progress: a report of the Surgeon General. Atlanta, GA: US Department of Health and Human Services, Centers for Disease ...; 2014.

7. Cancello R, Soranna D, Zambra G, Zambon A, Invitti C. Determinants of the lifestyle changes during COVID-19 pandemic in the residents of Northern Italy. *International journal of environmental research and public health*. 2020;17(17):6287.

8. Chopra S, Ranjan P, Singh V, et al. Impact of COVID-19 on lifestyle-related behaviours-a cross-sectional audit of responses from nine hundred and ninety-five participants from India. *Diabetes & Metabolic Syndrome: Clinical Research & Reviews*. 2020;14(6):2021-2030.

9. Tattan-Birch H, Perski O, Jackson S, Shahab L, West R, Brown J. COVID-19, smoking, vaping and quitting: a representative population survey in England. *Addiction*. 2020;

10. Caponnetto P, Inguscio L, Saitta C, Maglia M, Benfatto F, Polosa R. Smoking behavior and psychological dynamics during COVID-19 social distancing and stay-at-home policies: A survey. *Health psychology research*. 2020;8(1)

11. Wood W, Tam L, Witt MG. Changing circumstances, disrupting habits. *Journal of personality and social psychology*. 2005;88(6):918.

12. Stanton R, To QG, Khalesi S, et al. Depression, Anxiety and Stress during COVID-19: Associations with Changes in Physical Activity, Sleep, Tobacco and Alcohol Use in Australian Adults. *Int J Environ Res Public Health*. Jun 7 2020;17(11)doi:10.3390/ijerph17114065

13. Fancourt D, Bu F, Mak HW, Steptoe A. COVID-19 social study. *Results release*. 2020;15

14. Volkow ND. Collision of the COVID-19 and addiction epidemics. American College of Physicians; 2020.

15. Parrott AC. Does cigarette smoking cause stress? *American Psychologist*. 1999;54(10):817.

16. García-Álvarez L, Fuente-Tomás L, Sáiz PA, García-Portilla MP, Bobes J. Will changes in alcohol and tobacco use be seen during the COVID-19 lockdown? *Adicciones*. Apr 1 2020;32(2):85-89. ¿Se observarán cambios en el consumo de alcohol y tabaco durante el confinamiento por COVID-19? doi:10.20882/adicciones.1546

For peer review only - http://bmjopen.bmj.com/site/about/guidelines.xhtml

17. Adriaens K, Van Gucht D, Baeyens F. Differences between dual users and switchers center around vaping behavior and its experiences rather than beliefs and attitudes. *International journal of environmental research and public health*. 2018;15(1):12.

18. Grana RA, Popova L, Ling PM. A longitudinal analysis of electronic cigarette use and smoking cessation. *JAMA internal medicine*. 2014;174(5):812-813.

19. Etter JF, Bullen C. Electronic cigarette: users profile, utilization, satisfaction and perceived efficacy. *Addiction*. 2011;106(11):2017-2028.

20. Kim SY, Park JE, Lee YJ, et al. Testing a tool for assessing the risk of bias for nonrandomized studies showed moderate reliability and promising validity. *Journal of clinical epidemiology*. 2013;66(4):408-414.

21. Wohlin C. Guidelines for snowballing in systematic literature studies and a replication in software engineering. 2014:1-10.

22. Greenhalgh T, Peacock R. Effectiveness and efficiency of search methods in systematic reviews of complex evidence: audit of primary sources. *Bmj*. 2005;331(7524):1064-1065.

23. Soule EK, Mayne S, Snipes W, Guy MC, Breland A, Fagan P. Impacts of COVID-19 on electronic cigarette purchasing, use and related behaviors. *International Journal of Environmental Research and Public Health*. 2020;17(18):6762.

24. Caponnetto P, Inguscio L, Saitta C, Maglia M, Benfatto F, Polosa R. Smoking behavior and psychological dynamics during COVID-19 social distancing and stay-at-home policies: A survey. *Health Psychol Res*. May 27 2020;8(1):9124. doi:10.4081/hpr.2020.9124

25. Ren Y, Qian W, Li Z, et al. Public mental health under the long-term influence of COVID-19 in China: Geographical and temporal distribution. *Journal of Affective Disorders*. 2020;277:893-900.

26. Ozcelik N, Yilmaz Kara B. Effect of coronaphobia on smoking habits. *Journal of addictive diseases*. 2020:1-9.

27. Chertok IRA. Perceived risk of infection and smoking behavior change during COVID-19 in Ohio. *Public Health Nursing*. 2020;37(6):854-862.

28. Jackson SE, Garnett C, Shahab L, Oldham M, Brown J. Association of the Covid-19 lockdown with smoking, drinking, and attempts to quit in England: an analysis of 2019-2020 data. *medRxiv*. 2020;

29. Kayhan Tetik B, Gedik Tekinemre I, Taş S. The Effect of the COVID-19 Pandemic on Smoking Cessation Success. *J Community Health*. Jul 8 2020:1-5. doi:10.1007/s10900-020-00880-2

30. Kowitt SD, Cornacchione Ross J, Jarman KL, et al. Tobacco quit intentions and behaviors among cigar smokers in the United States in response to COVID-19. *International journal of environmental research and public health*. 2020;17(15):5368.

31. Jackson SE, Brown J, Shahab L, Steptoe A, Fancourt D. COVID-19, smoking and inequalities: a study of 53 002 adults in the UK. *Tobacco control*. 2020;

For peer review only

For peer review only - http://bmjopen.bmj.com/site/about/guidelines.xhtml

32. Stanton R, To QG, Khalesi S, et al. Depression, anxiety and stress during COVID-19: associations with changes in physical activity, sleep, tobacco and alcohol use in Australian adults. *International journal of environmental research and public health*. 2020;17(11):4065.
33. Jackson SE, Garnett C, Shahab L, Oldham M, Brown J. Association of the COVID-19 lockdown with smoking, drinking and attempts to quit in England: an analysis of 2019–20 data. *Addiction*. 2021;116(5):1233-1244.
34. Tetik BK, Tekinemre IG, Taş S. The effect of the COVID-19 pandemic on smoking cessation success. *Journal of Community Health*. 2020:1-5.
35. Goniewicz ML, Knysak J, Gawron M, et al. Levels of selected carcinogens and toxicants in vapour from electronic cigarettes. *Tobacco control*. 2014;23(2):133-139.
36. Pierce JP, Benmarhnia T, Chen R, et al. Role of e-cigarettes and pharmacotherapy during attempts to quit cigarette smoking: The PATH Study 2013-16. *PloS one*. 2020;15(9):e0237938.
37. Pasquereau A, Guignard R, Andler R, Nguyen-Thanh V. Electronic cigarettes, quit attempts and smoking cessation: a 6-month follow-up. *Addiction*. 2017;112(9):1620-1628.
38. Borrelli B, Hayes RB, Dunsiger S, Fava JL. Risk perception and smoking behavior in medically ill smokers: a prospective study. *Addiction*. 2010;105(6):1100-1108.
39. Weinstein ND. The precaution adoption process. *Health psychology*. 1988;7(4):355.
40. Klemperer EM, West JC, Peasley-Miklus C, Villanti AC. Change in Tobacco and Electronic Cigarette Use and Motivation to Quit in Response to COVID-19. *Nicotine Tob Res*. Aug 24 2020;22(9):1662-1663. doi:10.1093/ntr/ntaa072

For peer review only - http://bmjopen.bmj.com/site/about/guidelines.xhtml

Figure caption/ legend

Figure 1 Flow chart diagram

Legend : The flowchart shows the article selection process, the criteria for exclusion of articles, and the number of articles excluded.

Figure 2 Assessment of the risk of bias in identified studies using the RoBANS questionnaire

Legend : Author judgment for each RoBANS domain for the included articles. Green indicates a low risk of bias, yellow indicates unclear risk, and red indicates a high risk of bias. The six domains are "comparability of groups, participant selection, confounding variables, measurement of exposure, blinding of the outcome, incomplete outcome data, selective outcome reporting."

Figure 3 Assessment summary of the risk of bias in identified studies using the RoBANS questionnaire

Legend : Author judgment for each RoBANS domain for the included articles. Green circles indicate low risk of bias, yellow circles indicate unclear risk, and red circles indicate high risk of bias. The study numbers indicate the order of the articles presented in Table 1.

For peer review only - http://bmjopen.bmj.com/site/about/guidelines.xhtml

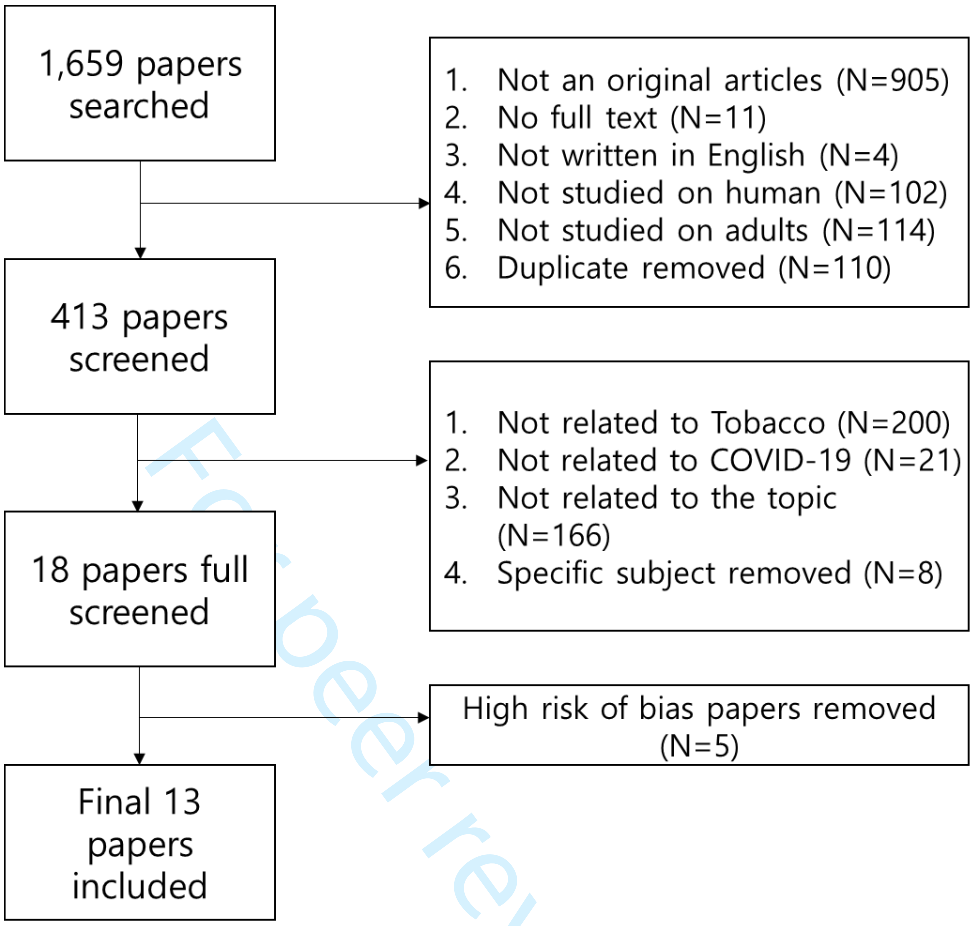

For peer review only

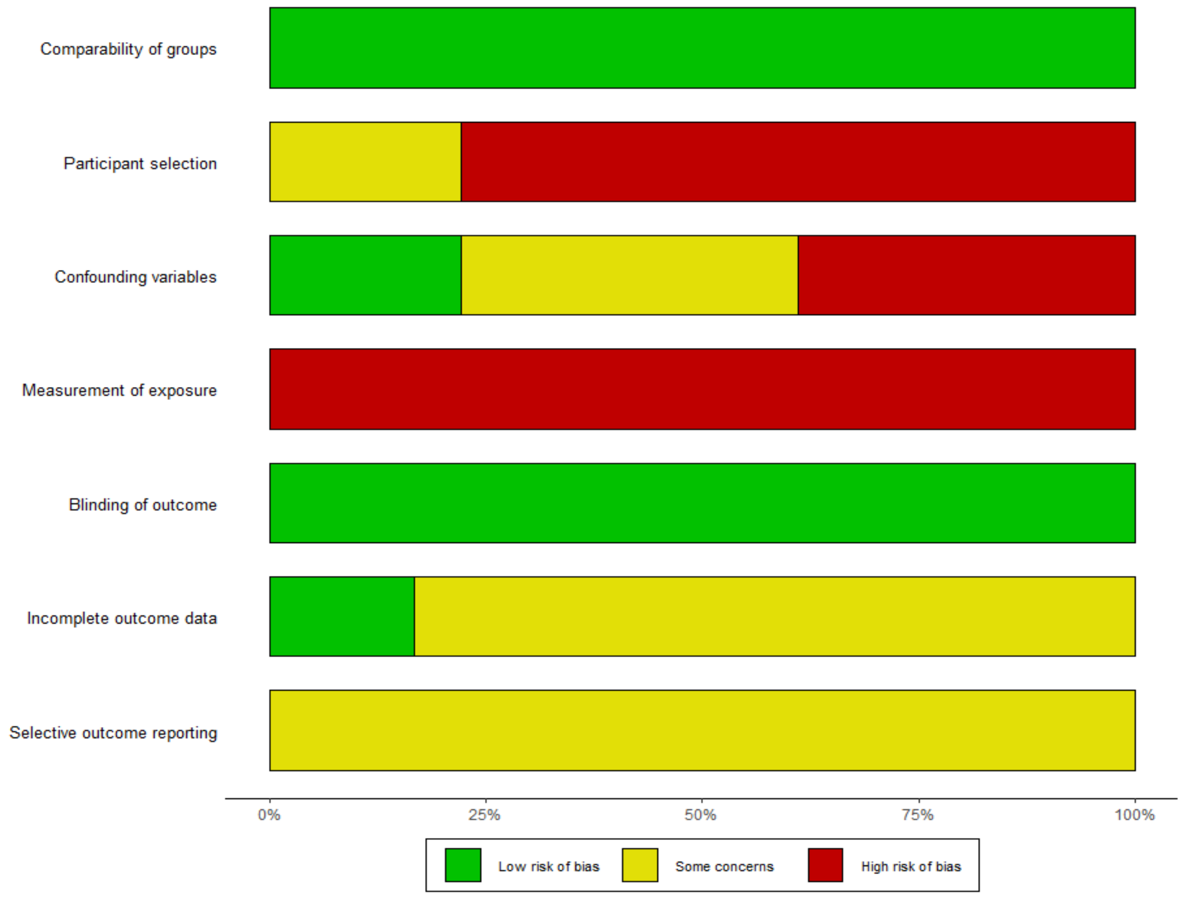
Comparability of groups
Participant selection
Confounding variables
Measurement of exposure
Blinding of outcome
Incomplete outcome data
Selective outcome reporting
0%
25%
50%
75%
100%
Low risk of bias
Some concerns
High risk of bias

view only

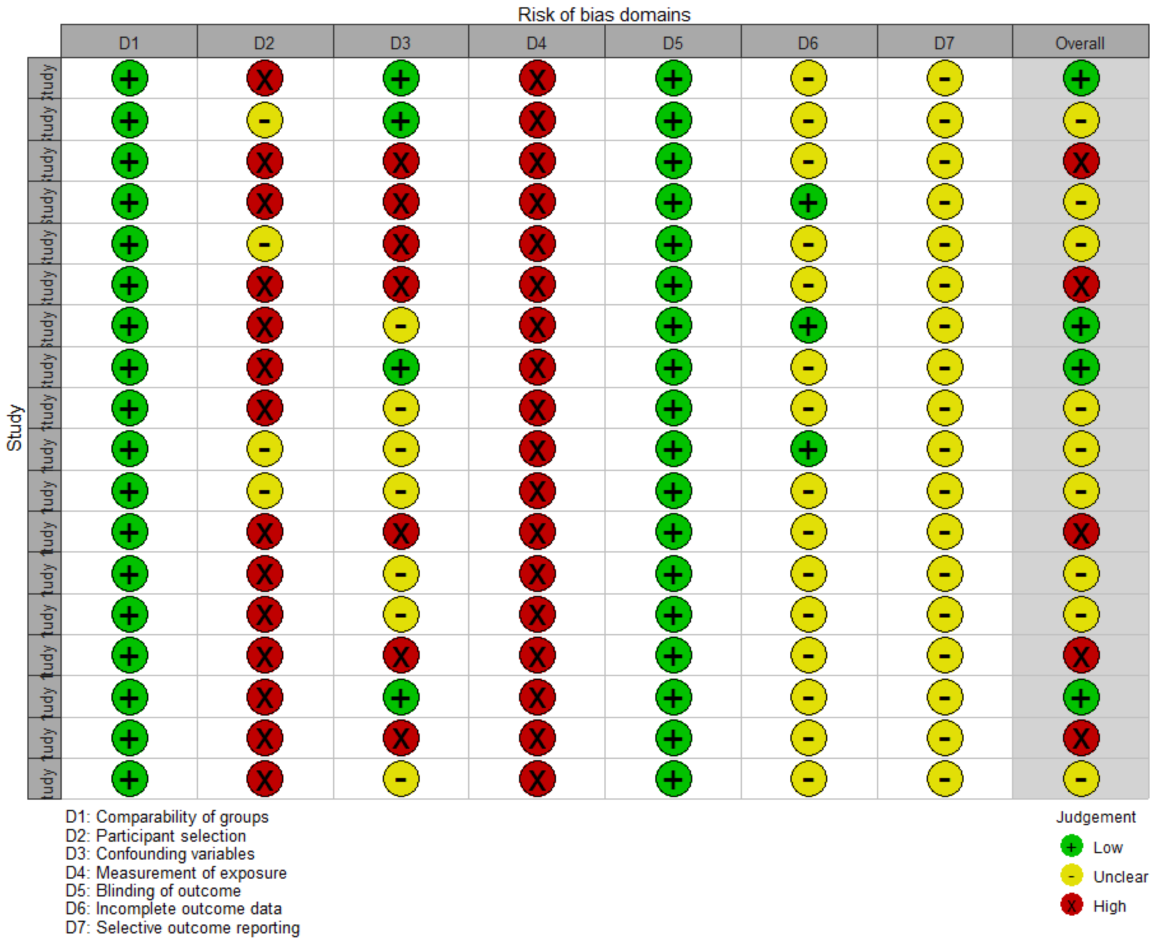
Risk of bias domains
D1
D2
D3
D4
D5
D6
D7
Overall
Study
D1: Comparability of groups
D2: Participant selection
D3: Confounding variables
D4: Measurement of exposure
D5: Blinding of outcome
D6: Incomplete outcome data
D7: Selective outcome reporting
Judgement
Low
Unclear
High

Supplemantary

Online Appendix : Search terms

Table 1 Pubmed search terms

Electronic Nicotine Delivery Systems
(((((((((((((((((((((((((((((((((((((((Electronic Nicotine Delivery System[Title/Abstract]) OR (Electronic Cigarettes[Title/Abstract])) OR (E-Cigs[Title/Abstract])) OR (E Cigs[Title/Abstract])) OR (E-Cig[Title/Abstract])) OR (E Cig[Title/Abstract])) OR (E-Cigarettes[Title/Abstract])) OR (E Cigarettes[Title/Abstract])) OR (E-Cigarette[Title/Abstract])) OR (E Cigarette[Title/Abstract])) OR (Electronic Cigarette[Title/Abstract])) OR (Cigarette, Electronic[Title/Abstract])) OR (Cigarettes, Electronic[Title/Abstract])) OR ("Electronic Nicotine Delivery Systems"[Mesh])) OR ("Vaping"[Mesh])) OR (E-Cig Use[Title/Abstract])) OR (E Cig Use[Title/Abstract])) OR (E-Cig Uses[Title/Abstract])) OR (Use, E-Cig[Title/Abstract])) OR (ECig Use[Title/Abstract])) OR (ECig Uses[Title/Abstract])) OR (Use, ECig[Title/Abstract])) OR (Vape[Title/Abstract])) OR (Vapes[Title/Abstract])) OR (E-Cigarette Use[Title/Abstract])) OR (E Cigarette Use[Title/Abstract])) OR (E-Cigarette Uses[Title/Abstract])) OR (Use, E-Cigarette[Title/Abstract])) OR (Nicotine Vaping[Title/Abstract])) OR (Nicotine Vapings[Title/Abstract])) OR (Vaping, Nicotine[Title/Abstract])) OR (Vapings, Nicotine[Title/Abstract])) OR (Ecigarette Use[Title/Abstract])) OR (Ecigarette Uses[Title/Abstract])) OR (Use, Ecigarette[Title/Abstract])) OR (Uses, Ecigarette[Title/Abstract])) OR (Electronic Cigarette Use[Title/Abstract])) OR (Cigarette Use, Electronic[Title/Abstract])) OR (Electronic Cigarette Uses[Title/Abstract])) OR (Use, Electronic Cigarette[Title/Abstract])
COVID-19
(((((((((((((((((((((((((((((((((((COVID 19[Title/Abstract]) OR (COVID-19 Virus Disease[Title/Abstract])) OR (COVID 19 Virus Disease[Title/Abstract])) OR (COVID-19 Virus Diseases[Title/Abstract])) OR (Disease, COVID-19 Virus[Title/Abstract])) OR (Virus Disease, COVID-19[Title/Abstract])) OR (COVID-19 Virus Infection[Title/Abstract])) OR (COVID 19 Virus Infection[Title/Abstract])) OR (COVID-19 Virus Infections[Title/Abstract])) OR (Infection, COVID-19 Virus[Title/Abstract])) OR (Virus Infection, COVID-19[Title/Abstract])) OR (2019-nCoV Infection[Title/Abstract])) OR (2019 nCoV Infection[Title/Abstract])) OR (2019-nCoV Infections[Title/Abstract])) OR (Infection, 2019-nCoV[Title/Abstract])) OR (Coronavirus Disease-19[Title/Abstract])) OR (Coronavirus Disease 19[Title/Abstract])) OR (2019 Novel Coronavirus Disease[Title/Abstract])) OR (2019 Novel Coronavirus Infection[Title/Abstract])) OR (2019-nCoV Disease[Title/Abstract])) OR (2019 nCoV Disease[Title/Abstract])) OR (2019-nCoV Diseases[Title/Abstract])) OR (Disease, 2019-nCoV[Title/Abstract])) OR (COVID19[Title/Abstract])) OR (Coronavirus Disease 2019[Title/Abstract])) OR (Disease 2019, Coronavirus[Title/Abstract])) OR (SARS Coronavirus 2 Infection[Title/Abstract])) OR (Infection, SARS-CoV-2[Title/Abstract])) OR (SARS-CoV-2 Infection[Title/Abstract])) OR (SARS CoV 2 Infection[Title/Abstract])) OR (SARS-CoV-2 Infections[Title/Abstract])) OR (COVID-19 Pandemic[Title/Abstract])) OR (COVID 19 Pandemic[Title/Abstract])) OR (COVID-19 Pandemics[Title/Abstract])) OR (Pandemic, COVID-19[Title/Abstract])) OR ("COVID-19"[Mesh])
Tobacco, Tobacco Products, Tobacco Use
(((((Tobacco Uses[Title/Abstract]) OR (Tobacco Consumption[Title/Abstract])) OR

(Consumption, Tobacco[Title/Abstract])) OR ("Tobacco Use"[Mesh])) OR (((((((((((((((Tobaccos[Title/Abstract]) OR (Nicotiana[Title/Abstract])) OR (Nicotianas[Title/Abstract])) OR (Nicotiana tabacum[Title/Abstract])) OR (Nicotiana tabacums[Title/Abstract])) OR (tabacum, Nicotiana[Title/Abstract])) OR ("Tobacco"[Mesh])) OR ("Smoking"[Mesh])) OR (Smoking Behaviors[Title/Abstract])) OR (Behavior, Smoking[Title/Abstract])) OR (Behaviors, Smoking[Title/Abstract])) OR (Smoking Behavior[Title/Abstract])) OR (Smoking Habit[Title/Abstract])) OR (Habit, Smoking[Title/Abstract])) OR (Habits, Smoking[Title/Abstract])) OR (Smoking Habits[Title/Abstract]))) OR ((((((((((((((((((((((((Product, Tobacco[Title/Abstract]) OR (Products, Tobacco[Title/Abstract])) OR (Tobacco Product[Title/Abstract])) OR (Cigarillos[Title/Abstract])) OR (Cigarillo[Title/Abstract])) OR (Pipe Tobacco[Title/Abstract])) OR (Pipe Tobaccos[Title/Abstract])) OR (Tobacco, Pipe[Title/Abstract])) OR (Cigars[Title/Abstract])) OR (Tobaccos, Pipe[Title/Abstract])) OR (Cigar[Title/Abstract])) OR (Kreteks[Title/Abstract])) OR (Kretek[Title/Abstract])) OR (Kreteks Tobacco[Title/Abstract])) OR (Kreteks Tobaccos[Title/Abstract])) OR (Tobacco, Kreteks[Title/Abstract])) OR (Tobaccos, Kreteks[Title/Abstract])) OR (Bidis[Title/Abstract])) OR (Bidi[Title/Abstract])) OR (Bidis Tobacco[Title/Abstract])) OR (Bidis Tobaccos[Title/Abstract])) OR (Tobacco, Bidis[Title/Abstract])) OR (Cigarettes[Title/Abstract])) OR (Cigarette[Title/Abstract])) OR ("Tobacco Products"[Mesh]))
Psychology
(("Psychology"[Mesh]) OR ("Psychology/diagnosis"[Mesh] OR "Psychology/psychology"[Mesh])) OR (((((((((((((Side Effects, Psychological[Title/Abstract]) OR (Psychological Side Effect[Title/Abstract])) OR (Side Effect, Psychological[Title/Abstract])) OR (Psychological Side Effects[Title/Abstract])) OR (Psychosocial Factors[Title/Abstract])) OR (Factor, Psychosocial[Title/Abstract])) OR (Factors, Psychosocial[Title/Abstract])) OR (Psychosocial Factor[Title/Abstract])) OR (Psychological Factors[Title/Abstract])) OR (Factor, Psychological[Title/Abstract])) OR (Psychological Factor[Title/Abstract])) OR (Factors, Psychological[Title/Abstract])) OR (Psychologist[Title/Abstract])) OR (Psychologists[Title/Abstract]))
Anxiety
((((((((Angst[Title/Abstract]) OR (Nervousness[Title/Abstract])) OR (Hypervigilance[Title/Abstract])) OR (Anxiousness[Title/Abstract])) OR (Social Anxiety[Title/Abstract])) OR (Anxieties, Social[Title/Abstract])) OR (Anxiety, Social[Title/Abstract])) OR (Social Anxieties[Title/Abstract])) OR (("Anxiety"[Mesh]) OR ("Anxiety/analysis"[Mesh] OR "Anxiety/diagnosis"[Mesh] OR "Anxiety/psychology"[Mesh]))
Stress, Psychological
((((((((((((Psychological Stresses[Title/Abstract]) OR (Stresses, Psychological[Title/Abstract])) OR (Life Stress[Title/Abstract])) OR (Life Stresses[Title/Abstract])) OR (Stress, Life[Title/Abstract])) OR (Stresses, Life[Title/Abstract])) OR (Stress, Psychologic[Title/Abstract])) OR (Psychologic Stress[Title/Abstract])) OR (Stressor, Psychological[Title/Abstract])) OR (Psychological Stressor[Title/Abstract])) OR (Psychological Stressors[Title/Abstract])) OR (Stressors, Psychological[Title/Abstract])) OR (Psychological Stress[Title/Abstract])) OR (("Stress, Psychological"[Mesh]) OR ("Stress, Psychological/analysis"[Mesh] OR "Stress, Psychological/diagnosis"[Mesh] OR "Stress, Psychological/psychology"[Mesh]))
Depression

(("Depression"[Mesh]) OR ("Depression/analysis"[Mesh] OR "Depression/diagnosis"[Mesh] OR "Depression/psychology"[Mesh])) OR ((((((((Depressions[Title/Abstract]) OR (Depressive Symptoms[Title/Abstract])) OR (Depressive Symptom[Title/Abstract])) OR (Symptom, Depressive[Title/Abstract])) OR (Symptoms, Depressive[Title/Abstract])) OR (Emotional Depression[Title/Abstract])) OR (Depression, Emotional[Title/Abstract])) OR (Depressions, Emotional[Title/Abstract])) OR (Emotional Depressions[Title/Abstract]))

Table 2 Science Direct search terms

Query
COVID AND (e-cigarette OR "electronic cigarette" OR "electronic nicotine delivery" OR "vaping" OR "heat not burn" OR "heated tobacco product")
COVID AND (tobaccos OR cigarette OR kretek OR bidis OR "pipe tobacco" OR cigarillos)

For peer review only

PRISMA 2020 Checklist

Section and Topic	Item #	Checklist item	Location where item is reported
**TITLE**			
Title	1	Identify the report as a systematic review.	Page 1
**ABSTRACT**			
Abstract	2	See the PRISMA 2020 for Abstracts checklist.	Page 2
**INTRODUCTION**			
Rationale	3	Describe the rationale for the review in the context of existing knowledge.	Page 5
Objectives	4	Provide an explicit statement of the objective(s) or question(s) the review addresses.	Page 7
**METHODS**			
Eligibility criteria	5	Specify the inclusion and exclusion criteria for the review and how studies were grouped for the syntheses.	Pages 8-9
Information sources	6	Specify all databases, registers, websites, organisations, reference lists and other sources searched or consulted to identify studies. Specify the date when each source was last searched or consulted.	Page 8
Search strategy	7	Present the full search strategies for all databases, registers and websites, including any filters and limits used.	Pages 8-9
Selection process	8	Specify the methods used to decide whether a study met the inclusion criteria of the review, including how many reviewers screened each record and each report retrieved, whether they worked independently, and if applicable, details of automation tools used in the process.	Page 9
Data collection process	9	Specify the methods used to collect data from reports, including how many reviewers collected data from each report, whether they worked independently, any processes for obtaining or confirming data from study investigators, and if applicable, details of automation tools used in the process.	Pages 8-9
Data items	10a	List and define all outcomes for which data were sought. Specify whether all results that were compatible with each outcome domain in each study were sought (e.g. for all measures, time points, analyses), and if not, the methods used to decide which results to collect.	Pages 8-9
	10b	List and define all other variables for which data were sought (e.g. participant and intervention characteristics, funding sources). Describe any assumptions made about any missing or unclear information.	Pages 8-9
Study risk of bias assessment	11	Specify the methods used to assess risk of bias in the included studies, including details of the tool(s) used, how many reviewers assessed each study and whether they worked independently, and if applicable, details of automation tools used in the process.	Pages 8-9
Effect measures	12	Specify for each outcome the effect measure(s) (e.g. risk ratio, mean difference) used in the synthesis or presentation of results.	Pages 8-9
Synthesis methods	13a	Describe the processes used to decide which studies were eligible for each synthesis (e.g. tabulating the study intervention characteristics and comparing against the planned groups for each synthesis (item #5)).	Pages 8-9
	13b	Describe any methods required to prepare the data for presentation or synthesis, such as handling of missing summary statistics, or data conversions.	Pages 8-9
	13c	Describe any methods used to tabulate or visually display results of individual studies and syntheses.	Pages 8-9
	13d	Describe any methods used to synthesize results and provide a rationale for the choice(s). If meta-analysis was performed, describe the model(s), method(s) to identify the presence and extent of statistical heterogeneity, and software package(s) used.	Meta-analysis is not performed
	13e	Describe any methods used to explore possible causes of heterogeneity among study results (e.g. subgroup analysis, meta-regression).	Meta-analysis is not performed
	13f	Describe any sensitivity analyses conducted to assess robustness of the synthesized results.	Meta-analysis is not

For peer review only - http://bmjopen.bmj.com/site/about/guidelines.xhtml

PRISMA 2020 Checklist

Section and Topic	Item #	Checklist item	Location where item is reported
			performed
Reporting bias assessment	14	Describe any methods used to assess risk of bias due to missing results in a synthesis (arising from reporting biases).	Pages 8-9
Certainty assessment	15	Describe any methods used to assess certainty (or confidence) in the body of evidence for an outcome.	Meta-analysis is not performed
**RESULTS**			
Study selection	16a	Describe the results of the search and selection process, from the number of records identified in the search to the number of studies included in the review, ideally using a flow diagram.	Pages 8-10
	16b	Cite studies that might appear to meet the inclusion criteria, but which were excluded, and explain why they were excluded.	Pages 8-10
Study characteristics	17	Cite each included study and present its characteristics.	Page 11
Risk of bias in studies	18	Present assessments of risk of bias for each included study.	Figures 2 and 3
Results of individual studies	19	For all outcomes, present, for each study: (a) summary statistics for each group (where appropriate) and (b) an effect estimate and its precision (e.g. confidence/credible interval), ideally using structured tables or plots.	Pages 12-15
Results of syntheses	20a	For each synthesis, briefly summarise the characteristics and risk of bias among contributing studies.	Page 11-12
	20b	Present results of all statistical syntheses conducted. If meta-analysis was done, present for each the summary estimate and its precision (e.g. confidence/credible interval) and measures of statistical heterogeneity. If comparing groups, describe the direction of the effect.	Meta-analysis is not performed
	20c	Present results of all investigations of possible causes of heterogeneity among study results.	Meta-analysis is not performed
	20d	Present results of all sensitivity analyses conducted to assess the robustness of the synthesized results.	Meta-analysis is not performed
Reporting biases	21	Present assessments of risk of bias due to missing results (arising from reporting biases) for each synthesis assessed.	Figures 2 and 3 Meta-analysis is not performed
Certainty of evidence	22	Present assessments of certainty (or confidence) in the body of evidence for each outcome assessed.	Meta-analysis is not performed
**DISCUSSION**			
Discussion	23a	Provide a general interpretation of the results in the context of other evidence.	Page 20-22
	23b	Discuss any limitations of the evidence included in the review.	Page 23

For peer review only

PRISMA 2020 Checklist

Section and Topic	Item #	Checklist item	Location where item is reported
	23c	Discuss any limitations of the review processes used.	Page 23
	23d	Discuss implications of the results for practice, policy, and future research.	Pages 22-23
**OTHER INFORMATION**			
Registration and protocol	24a	Provide registration information for the review, including register name and registration number, or state that the review was not registered.	X
	24b	Indicate where the review protocol can be accessed, or state that a protocol was not prepared.	X
	24c	Describe and explain any amendments to information provided at registration or in the protocol.	X
Support	25	Describe sources of financial or non-financial support for the review, and the role of the funders or sponsors in the review.	Page 24
Competing interests	26	Declare any competing interests of review authors.	Page 24
Availability of data, code and other materials	27	Report which of the following are publicly available and where they can be found: template data collection forms; data extracted from included studies; data used for all analyses; analytic code; any other materials used in the review.	Table 1

From: Page MJ, McKenzie JE, Bossuyt PM, Boutron I, Hoffmann TC, Mulrow CD, et al. The PRISMA 2020 statement: an updated guideline for reporting systematic reviews. BMJ 2021;372:n71. doi: 10.1136/bmj.n71

For more information, visit: http://www.prisma-statement.org/

For peer review only

BMJ Open

A systematic review of changed smoking behavior, smoking cessation, and psychological states of smokers according to cigarette type during the COVID-19 pandemic

Journal:	*BMJ Open*
Manuscript ID	bmjopen-2021-055179.R2
Article Type:	Original research
Date Submitted by the Author:	20-Apr-2022
Complete List of Authors:	CHUN, HAE-RYOUNG; Seoul National University,<br>Cheon, Eunsil; Seoul National University, Graduate school of public health<br>Hwang, Ji-eun; Seoul National University Institute of Health and Environment,
**Primary Subject Heading**:	Smoking and tobacco
Secondary Subject Heading:	Epidemiology, Public health, Smoking and tobacco
Keywords:	COVID-19, PUBLIC HEALTH, MENTAL HEALTH

SCHOLARONE™
Manuscripts

I, the Submitting Author has the right to grant and does grant on behalf of all authors of the Work (as defined in the below author licence), an exclusive licence and/or a non-exclusive licence for contributions from authors who are: i) UK Crown employees; ii) where BMJ has agreed a CC-BY licence shall apply, and/or iii) in accordance with the terms applicable for US Federal Government officers or employees acting as part of their official duties; on a worldwide, perpetual, irrevocable, royalty-free basis to BMJ Publishing Group Ltd ("BMJ") its licensees and where the relevant Journal is co-owned by BMJ to the co-owners of the Journal, to publish the Work in this journal and any other BMJ products and to exploit all rights, as set out in our licence.

The Submitting Author accepts and understands that any supply made under these terms is made by BMJ to the Submitting Author unless you are acting as an employee on behalf of your employer or a postgraduate student of an affiliated institution which is paying any applicable article publishing charge ("APC") for Open Access articles. Where the Submitting Author wishes to make the Work available on an Open Access basis (and intends to pay the relevant APC), the terms of reuse of such Open Access shall be governed by a Creative Commons licence – details of these licences and which Creative Commons licence will apply to this Work are set out in our licence referred to above.

Other than as permitted in any relevant BMJ Author's Self Archiving Policies, I confirm this Work has not been accepted for publication elsewhere, is not being considered for publication elsewhere and does not duplicate material already published. I confirm all authors consent to publication of this Work and authorise the granting of this licence.

For peer review only

A systematic review of changed smoking behavior, smoking cessation, and psychological states of smokers according to cigarette type during the COVID-19 pandemic

1 Chun, Hae-ryoung; Seoul National University, Graduate school of public health; mamimi777@snu.ac.kr

2 Cheon, Eunsil; Seoul National University, Graduate school of public health; silverberry2@snu.ac.kr

* Correspondence to: Ji-eun Hwang; 1. Seoul National University Institute of Health and Environment
1, Gwanak-ro, Gwanak-gu, Republic of Korea; loshjeve@gmail.com

For peer review only

ABSTRACT

Objectives: Although the global coronavirus disease 2019 (COVID-19) pandemic has increased interest in research involving high-risk smokers, studies examining changed smoking behaviors, cessation intentions, and associated psychological states among smokers are still scarce. This study aimed to systematically review the literature related to this subject.

Design: A systematic review of published articles on cigarettes and COVID-19-related topics

Data Sources: Our search was conducted in January 2021. We used the keywords COVID-19, cigarettes, electronic cigarettes (e-cigarettes), and psychological factors in PubMed and ScienceDirect and found papers published between January and December 2020.

Data selection: We included articles in full text, written in English, and that surveyed adults. The topics included smoking behavior, smoking cessation, psychological state of smokers, and COVID-19-related topics.

Data extraction and synthesis: Papers of low quality, based on quality assessment, were excluded. Thirteen papers were related to smoking behavior, nine papers were related to smoking cessation, and four papers were related to psychological states of smokers.

Results: Owing to the COVID-19 lockdown, cigarette users were habituated to purchasing large quantities of cigarettes in advance. Additionally, cigarette-only users increased their attempts and willingness to quit smoking, compared with e-cigarette-only users.

Conclusions: Owing to the COVID-19 outbreak, the intention to quit smoking was different among smokers, according to cigarette type (cigarette-only users, e-cigarette-only users, and dual users). With the ongoing COVID-19 pandemic, policies and campaigns to increase smoking cessation intentions and attempts to quit smoking among smokers at high risk of COVID-19 should be implemented. Additionally, e-cigarette-only users with poor health-seeking behavior require interventions to increase the intention to quit smoking.

Keyword: COVID-19 Pandemic, Quitting Smoking, Smoking Habits

STRENGTHS AND LIMITATIONS OF THIS STUDY

- This study is the first to systematically review the literature regarding changes in smoking behaviors, cessation intentions, and psychological states of smokers, based on the study period, country, and age of participants during the coronavirus disease 2019 pandemic.
- According to the Risk of Bias Assessment Tool for Non-randomized Studies (RoBANS), three researchers cross-checked and evaluated the articles' quality and excluded papers with a high risk of bias.
- Using the backward snowballing method, screening for articles selection was strictly performed by checking for any additional papers.

For peer review only

- Since the articles collected for this systematic review were only cross-sectional designs, it was difficult to determine causality; therefore, only associations were presented.

For peer review only

For peer review only - http://bmjopen.bmj.com/site/about/guidelines.xhtml

1. INTRODUCTION

On January 30, 2020, the World Health Organization (WHO) declared coronavirus disease 2019 (COVID-19), an infectious disease of the respiratory tract characterized by severe acute respiratory syndrome,[1] a public health emergency of international concern (PHEIC).[2] In 2020, a total of 350,594,336 cases of COVID-19 were reported, including 7,893,839 deaths[3, 4]. Due to the pandemic, different standards and steps to control the spread of the disease have been adopted worldwide, but almost all countries have implemented lockdown and social distancing measures to prevent COVID-19 infection.

Smoking is a high-risk factor for COVID-19 infection, and many systematic reviews and meta-analyses report the association between COVID-19 and smoking.[5, 6] Since tobacco is associated with chronic lung disease, physicians could expect more cigarette users to develop a severe form of COVID-19,[5] given the effect of tobacco on respiratory disease and immune function.[7] Smoking is associated with negative progression and side effects of COVID-19.[6] Smoking, including current or previous smoking history, significantly increases the severity and risk of death from COVID-19.[8] Similar to cigarette use, e-cigarette use can cause oxidative stress and inflammatory response in the lungs, making e-cigarette users more susceptible to bacterial or viral infections.[9] Although no literature indicates that cigarettes are the direct cause of COVID-19 infection, smokers have a higher risk of severe COVID-19 symptoms than nonsmokers. [10] Previous studies have suggested several public health messages to continue focusing on smoking cessation efforts; however, smoking rates have not decreased.[11]

Few investigations have focused on whether smoking behavior[12] and cessation efforts[13] have changed during the COVID-19 pandemic. Previous studies have shown that smoking has decreased[12] and quitting attempts have increased[13] during the pandemic. Since lockdowns and various social distancing measures were implemented in the early stages of the COVID-19 pandemic, they have the potential to affect smoking behavior and cessation intention. In some countries, it is expected that smoking behavior will change given the difficulty in freely purchasing tobacco products as non-essential items; individuals are locked down under the pandemic and are required to stay at home for prolonged periods.[14] Changes in daily life due to social distancing and lockdowns are expected to help reduce smoking consumption.[15] Therefore, it is necessary to examine the factors influencing smoking behavior and cessation intention in the early stages of the pandemic, which have been investigated in the literature.

There are insufficient studies on smokers' negative psychological states during the COVID-19 pandemic. Further research is needed because the relationship between negative psychological states and health behavior is unclear.[16] People experience higher stress levels than usual because of social isolation, employment insecurity, finances uncertainty, responsibilities, and concerns about illness from the virus.[17] Therefore, many cigarette users smoke to relieve stress and negative emotions,[18] but it is often counterproductive.[19] Previous research has shown that negative psychological states, such as stress, anxiety, and depression, can influence tobacco-use behavior.[20]

There are insufficient studies to compare the smoking behavior, cessation intention, and psychological state of cigarette and e-cigarette users during the COVID-19 pandemic.[14] The

For peer review only

daily use of e-cigarettes among e-cigarette users slightly increased during the COVID-19 lockdown;[21] therefore, it is necessary to investigate whether smoking behavior changes according to the cigarette type. Cigarette users are aware that they engage in harmful health behaviors. However, it is unclear whether e-cigarettes help cigarette users quit smoking.[22] E-cigarette users believe e-cigarettes are less harmful to their health as they contain relatively fewer harmful ingredients than cigarettes.[23] Therefore, they believe that smoking e-cigarettes is an effort to quit smoking.[23]

In addition, after the COVID-19 pandemic, smoking behavior may change by age depending on the cigarette type. The use of e-cigarettes increases among persons in their early 20s compared with old persons.[24] In addition, it is known that older persons are more vulnerable to COVID-19 infection and can develop severe infection symptoms.[25] Therefore, there may be an increase in the older persons' intention to quit smoking cigarettes. However, young people who use e-cigarettes may not be less willing to quit smoking.

Many studies have investigated whether smoking is a high-risk factor for COVID-19.[6, 8] However, relatively few studies have been published on whether smoking behavior, cessation intention, and psychological state have changed during the COVID-19 pandemic. In the future, it is expected that this study will establish a scientific basis and policy response to smoking behavior, cessation intention, and psychological state based on cigarette type, both during and post COVID-19. Therefore, we conducted a systematic review based on the hypothesis that there would be changes in smoking behavior, cessation intention, and psychological states of adult smokers, depending on the tobacco product used during the COVID-19 pandemic.

For peer review only - http://bmjopen.bmj.com/site/about/guidelines.xhtml

2. METHODS

2.1. Literature Search Strategy

We systematically reviewed the data according to the Preferred Reporting Items for Systematic Reviews and Meta-Analyses (PRISMA), shown in the Supplementary file. We searched articles using MeSH terms ‘COVID-19, depression, stress, psychological, anxiety, tobacco, tobacco products, tobacco use, and electronic nicotine delivery system’ in January 2021. Only papers published between January and December 2020 were included in the study. All search terms are listed in Supplementary Table 1.

A literature search was conducted on PubMed using the following MeSH terms: "(depression OR stress OR anxiety OR psychology) AND ((tobacco OR tobacco products OR tobacco use OR electronic nicotine delivery system) AND COVID-19))" in the title or the abstract in 2020. A literature search was conducted on ScienceDirect using the following search terms: "COVID-19 AND (e-cigarette OR "electronic cigarette" OR "electronic nicotine delivery" OR "vaping" OR "heat not burn" OR "heated tobacco product")," as well as COVID-19 AND (tobaccos OR cigarette OR kretek OR bidis OR "pipe tobacco" OR cigarillos).

2.2. Inclusion and Exclusion Criteria

Figure 1 shows a flowchart of the database searches, followed by the exclusion/inclusion strategy. The inclusion criteria were cases that did not meet the exclusion criteria. In the first phase (Figure 1), certain types of articles were excluded, including comments, letters, editorials, viewpoints, correspondence, and articles without full text. In addition, duplicate works and those not written in English and studies that did not include humans or adults were excluded. In the next phase (Figure 1), studies unrelated to tobacco or COVID-19 were excluded. In addition, articles were excluded if the topics were not related to changes in tobacco use and mental health caused by COVID-19. Finally, articles with a high risk of bias were excluded using the Risk of Bias Assessment Tool for Non-randomized Studies (RoBANS) (Figures 2 and 3).[26]

2.3. Data Extraction

Two independent reviewers (HR and JE) screened the literature and assessed each paper by reading the titles, abstracts, and full texts. Three authors extracted data from studies that fulfilled our inclusion criteria independently (HR, JE, and ES). The primary data points included were the following: study details (author, journal, publication date, country, study design, study period, and funding), the total number of participants, types of cigarettes, changes in smoking behavior, cessation intention, and psychological state.

Two authors (HR and ES) independently assessed the quality of included studies using the RoBANS.[26] If the opinions of the two authors were different, the quality evaluation was completed through further discussion with another researcher (JE). The RoBANS contains six

For peer review only - http://bmjopen.bmj.com/site/about/guidelines.xhtml

domains: selection of participants, confounding variables, measurement of exposure, blinding of outcome assessments, incomplete outcome data, and selective outcome reporting (Figures 2 and 3). The risk of bias was divided into three stages (high, unclear, or low risk). When the risk of bias in a study was high, it was excluded.

Using the backward snowballing method,[27, 28] we checked the references of the articles that met the inclusion criteria. We checked the titles, journals, abstracts, and full texts of the references of the articles. We excluded articles that did not meet the inclusion criteria. In addition, articles not related to the subject of this study were excluded. As a result, no additional papers were added.

3. RESULTS

3.1. Literature Search and Literature Selection Results

A total of 1,659 papers were found in PubMed and ScienceDirect, and 905 non-original papers were excluded. Eleven papers without full text and four papers not written in English were excluded. In addition, 413 papers were selected, excluding 102 papers that were not conducted in humans, 114 papers that were not conducted in adults, and 110 duplicate papers after the first screening phase. Papers unrelated to tobacco (n = 200) and COVID-19 (n = 21) were excluded. In addition, a total of 18 papers were included, excluding 166 papers that were not related to tobacco behavior and psychological states that changed during the COVID-19 pandemic, as well as eight papers targeting COVID-19 patients or medical personnel in the first screening phase.

3.2. Assessment result of the risk of bias

13 papers were selected after excluding five papers with a high risk of bias, as determined through quality evaluation of the 18 papers using the RoBANS(Figures 2 and 3).

3.3. Characteristics of included papers by three subjects

All 13 selected papers were published in 2020; three were conducted in the US, three in the UK, two in Italy, two in Turkey, and one in China, India, and Australia. All were cross-sectional design studies that used surveys. Comments, letters, editorials, viewpoints, and correspondence were not included. In the included papers, the number of study participants varied from 93 to 52,002, and all participants were adults. We also presented the average age of the participants in the papers or the ratio by age group. Eight papers targeted cigarette-only users, and five reviewed e-cigarette and cigarette users together (Table 1). Table 1 shows that smokers' smoking behavior, cessation intentions, and psychological states changed due to the COVID-19 pandemic, presented in all 13 papers. Seven papers studied changes in smoking behavior, eight papers examined changes in smoking cessation, and five papers examined changes in

For peer review only - http://bmjopen.bmj.com/site/about/guidelines.xhtml

smokers' psychological state after the COVID-19 outbreak (Table 1).

3.4. Integrating results of the 13 selected papers

Changes in frequency of smoking occurred in the early stages of the COVID-19 pandemic, regardless of cigarette type. However, smoking cessation intentions and attempts were higher among cigarette-only users than e-cigarette-only users. When the average age of participants was presented, and only papers surveyed according to the cigarette type were examined, the smoking behavior of cigarette-only users was found to increase in the paper with an average age of 22. In a study targeting participants on average in their 50s or older, cigarette-only users showed a higher intention to quit smoking than e-cigarette-only users. Regardless of the average age of participants, in most studies, the amount of e-cigarette-only users' usage did not decrease, and the intention to quit smoking did not increase. In addition, it was found that all smokers, regardless of the tobacco product used, had increased stress and anxiety during the COVID-19 pandemic, and the more they did not intend to quit, the more stressed they were. We present detailed information on smoking behavior, smoking cessation, and psychological state changes according to cigarette type during the COVID-19 pandemic in the following six sub-headings.

3.4.1. Changes in smoking behavior in e-cigarette users (or dual users) during the COVID-19 outbreak

According to Soule et al.,[29] the higher the reliance on e-cigarettes, the higher the incidence of cigarette use. There have also been increasing efforts to reduce e-cigarette use among dual users. However, mastery of e-cigarette-specific behaviors during the additional time spent at home improved e-cigarette skills and increased e-cigarette use.[29] According to the article by P. Caponnetto et al.,[30] daily cigarette consumption by dual users of cigarettes and e-cigarettes and cigarette users decreased slightly. Due to the COVID-19 outbreak, purchasing products, including tobacco products, has changed; individuals buy large numbers of products at once.

3.4.2. Changes in smoking behavior in cigarette-only users during the COVID-19 outbreak

According to the results of articles by Cancello et al.[11] and Ren et al.,[31] only cigarette consumption increased by 38% and 30%, respectively. Compared to before the COVID-19 pandemic, Chopra et al. showed a significant decrease in smoking consumption (0.02 [0.03], P <0.05).[12] However, in the study by Ozcelik et al.,[32] 67.5% of participants reported no change in smoking behavior, which cannot be interpreted as a significant change in smoking behavior during the COVID-19 pandemic.

3.4.3. Changes in intentions and attempts to quit smoking among e-cigarette users (or dual users) during the COVID-19 outbreak

For peer review only - http://bmjopen.bmj.com/site/about/guidelines.xhtml

Tattan-Birch et al.[13] found that a few cigarette-only users and e-cigarette-only users attempted to quit smoking due to the COVID-19 outbreak. Approximately one in 10 current e-cigarette-only users reported an attempt to quit vaping because of COVID-19. Soule et al. reported that users with high reliance on e-cigarettes exerted less effort to reduce product usage than users with low reliance. [29] According to a study by Caponnetto et al., while most cigarette-only users have considered quitting smoking, most e-cigarette-only users have not considered stopping e-cigarette use.[14] According to a study by Chertok et al., 36.7% of all smokers attempted to quit smoking during the pandemic regardless of cigarette type; people who quit smoking due to COVID-19 perceived smoking as a high-risk factor COVID-19 infection.[33]

3.4.4. Changes in intentions and attempts to quit smoking among cigarette users during the COVID-19 outbreak

According to Jackson et al.,[34] COVID-19 shutdowns have increased attempts to quit smoking. According to the results of the article by Tetik et al.,[35] which compared the success rates of smoking cessation before and during the pandemic, the COVID-19 outbreak effectively promoted smoking cessation. Only 12.8% of people who quit smoking during the COVID-19 outbreak did not restart smoking. According to Ozcelik et al.,[32] people with coronaphobia exhibited a significantly higher decrease or cessation in smoking than those with no change in smoking behavior or increased smoking consumption. A study of cigarette-only users found that the number of attempts to quit smoking had increased in the US.[33,36] In the UK, all studies conducted on cigarette-only users found that attempts to quit smoking had increased.[13, 37] In the case of cigarette-only users, there are more successful quitting smoking cases than e-cigarette-only users.[13]

3.4.5. The association between smoking and psychological changes in e-cigarette users (or dual users) during the COVID-19 outbreak

According to Soule et al., the greater the dependence on e-cigarettes, the higher the concern about COVID-19 infection than those with low dependence.[29] In the article by S.D. Kowitt et al., the higher the awareness regarding the risk of contracting COVID-19, regardless of cigarette type, the greater the willingness to quit ($B = 0.38$, $p < 0.001$) and the attempt to quit smoking (OR: 1.31, 95% CI: 1.04–1.64).[36] In addition, as social distancing ($B = 0.11$, $p = 0.01$) was enforced more strongly, the willingness to quit smoking increased.[36]

3.4.6. The association between smoking and psychological changes in cigarette users during the COVID-19 outbreak

Stanton et al. found that people with increased tobacco consumption had a higher risk of depression, anxiety, and stress. [38] Jackson et al. reported that compared to non-smokers, current

For peer review only - http://bmjopen.bmj.com/site/about/guidelines.xhtml

and former cigarette-only users had increased stress from COVID-19.[39] Ozcelik et al. indicated that among people with coronaphobia, those who reduced or stopped smoking felt more anxious than those who increased their smoking frequency. [32]

For peer review only

For peer review only - http://bmjopen.bmj.com/site/about/guidelines.xhtml

Table 1 Characteristics of included papers by three subjects

First Author,<br>Setting<br>Period	Age	Type of cigarette(N,%)	Subject 1*<br>Smoking behavior	Subject 2*<br>Smoking cessation	Subject 3*<br>Psychological states
H. Tattan-Birch[13]<br>England<br>April-May	Mean(SD)<br>52.4 (17.6)	Total : 3,179<br>-Cigarette-only users<br>Never:1,804(56.7%)<br>Long term ex-cigarette users<br>: 834 (26.2%)<br>Recent ex-cigarette users<br>: 72 (2.3%)<br>Current smoker : 469 (14.8%)<br>-E-cigarettes-only users<br>no current use : 2,987 (94%)<br>current use : 192 (6%)		Quit Attempt<br>- Cigarette-only users: 12.2%<br>- E-cig: 10%	
E.K. Soule[29]<br>US<br>April	Mean(SD)<br>35.1(10.8)	-Total: E-cig use (n=93)<br>- Dual user of E-cig and the following products<br>1) Cigarettes: 50<br>2) Cigar: 11<br>3) Cigarillo or little cigar: 19<br>4) Smokeless: 7<br>5) Waterpipe: 11	1. Efforts to reduce the use of e-cigs have increased for dual users (Mean = 3.71 vs. 3.02; p < 0.05), but e-cig use skills have increased (Mean = 3.48 vs. 2.27; p < 0.05) and usage has increased (Mean = 4.34 vs. 3.72; p < 0.05).<br>2. Users with high dependence on e-cigs have increased use of e-cigs compared to users with low dependence.<br>(Mean = 4.62 vs. 3.35; p < 0.001)	Users depended more on e-cigs had less effort to reduce their use of e-cigs (Mean = 3.10, SD = 0.63) than users who were less dependent on e-cigs (Mean = 3.79, SD = 0.61; p < 0.02).	Users who rely heavily on e-cigs are more concerned about COVID-19 than users who do not rely heavily on e-cigs (Mean = 3.86 vs. 3.06; p < 0.05).
Chertok[33]<br>US Ohio<br>April	Mean(SD)<br>33.5 (14.6)	Total: 810<br>1. Cigarettes-only: 115 (62.8%)<br>2. Dual users of cigarettes and e-cig: 68(37.2%)<br>3. Not smoker: 627		(Not classified according to the type of cigarette but surveyed for all smokers.)<br>- Attempt to quit: 36.7% (n = 66)<br>- Those who quit since COVID-19, those who desired to quit perceived a higher risk of infection.	

For peer review only - http://bmjopen.bmj.com/site/about/guidelines.xhtml

First Author, Setting Period	Age	Type of cigarette(N,%)	Subject 1* Smoking behavior	Subject 2* Smoking cessation	Subject 3* Psychological states
P.Caponnetto[14] Italy 2nd-26th April	Mean(SD) 34.7 (14.11)	Total: 1,825<br>Dual user of Cigarettes and E-Cigarettes (64,3.5%)<br>Cigarette and HTPs (33,1.8%),<br>Cigarette-only users (582, 32%)<br>HTPs-only user (81,4.4%)<br>E-cig-only users (225, 12.3%)<br>Former smoker (293, 16%)<br>Never smoker (547, 30%)	1. Dual users of cigarette and e-cig, and cigarette-only users' daily consumption has decreased. (Standardized residual (SR) value -2.7 and -1.9)<br>2. "Cigarette-only users" and the "E-cig-only users" changed the way of purchasing products, and the majority of them changed their habits by having large stocks of cigarettes or e-liquids/cartridges at home avoid going out every day.	Most cigarette-only users (SR=11.6) have considered quitting<br><br>E-cig-only users (SR=-4.2) have not considered stopping the use of e-cigs.	
S.D. Kowitt[36] US 23rd April-7th May	Mean(SD) 39.9 (13.4)	Total: 777<br>Cigarette-only users, 651 (83.8%)<br>E-cig only 293(37.3%)<br>Smokeless tobacco only 170(21.9%)<br>Waterpipe tobacco only 103(13.3%)			(Not classified according to the type of cigarette but surveyed for all smokers.)<br>-Having higher risk perceptions of COVID-19 was associated with higher quit intentions (B = 0.38, p < 0.001)<br>-Greater social distancing efforts were associated with higher quit intentions (B = 0.11, p = 0.01).<br>-Higher odds of quitting attempts since COVID-19 started (aOR: 1.31, 95% CI: 1.04, 1.64).
R. Stanton[38] Australia April	Mean(SD) 50.5 (14.9)	Total = 1,491<br>Cigarettes-only=172(11.5%)			Those who reported a negative change in smoking were more likely to have higher depression (aOR = 1.09, 95% CI = 1.04, 1.13), anxiety (aOR = 1.12, 95% CI = 1.06, 1.18), and stress (aOR = 1.10, 95% CI = 1.05, 1.15).
Jackson[37] England	18-29: 19.5%	Total: 53,002<br>Ex-cigarette users			Current and ex- cigarette users had higher odds than never smokers of

For peer review only - http://bmjopen.bmj.com/site/about/guidelines.xhtml

First Author, Setting Period	Age	Type of cigarette(N,%)	Subject 1* Smoking behavior	Subject 2* Smoking cessation	Subject 3* Psychological states
21st March-20th April	30's :15.0%<br>40's: 17.7%<br>50's: 17.5%<br>60's: 19.1%<br>≥70: 11.2%	Current cigarette users:13,602(25.7%)<br>Cigarette-only users: 8,057(15.2%)			reporting Significant stress about becoming seriously ill from the COVID-19 pandemic.<br>Current cigarette users<br>: OR=1.34, 95% CI = 1.27–1.43;<br>Ex- cigarette users<br>: OR=1.22, 95% CI = 1.16–1.28
Jackson[39] England March-April	18–24:13.4%<br>25–34:16.9%<br>35–44:15.7%<br>45–54:17.0%<br>55–64:14.5%<br>≥ 65: 22.5%	Cigarette-only users: 20,558	The COVID-19 lockdown was not associated with a significant change in smoking prevalence<br>17.0% (after) versus 15.9% (before), OR = 1.09, 95% CI = 0.95–1.24.	The COVID-19 lockdown was associated with increases in quit attempts 39.6 vs 29.1%,<br>aOR=1.56, 95%CI=1.23–1.98,<br>quit success 21.3 vs 13.9%,<br>aOR = 2.01, 95% CI = 1.22–3.33<br>and cessation (8.8 vs 4.1%,<br>aOR = 2.63, 95% CI = 1.69–4.09)<br>among past-year cigarette-only users	
Kayhan Tetik[40] Turkey May	Mean(SD)<br>39.5(12.1)	Cigarette-only users: 357		165(46.2%) cigarette-only users of participants, 86 (24.1%) did not quit despite this pandemic.	
Ren[31] China 14th February -29th March	Mean: 22.0 (min-max: 21.0–37.0)	Cigarette-only users:1,172	Some (30.1%) increased smoking during the pandemic.		
Ozcelik[32] Turkey 1stJanuary-30th June	Mean: 41.2 (min-max: 18–82)	Current cigarette-only users (N= 111, 98%)<br>Quitted smoking (N= 3, 2%)	77 people (67.5%) had no behavioral change associated with smoking	People with coronaphobia exhibited a significantly higher decrease or cessation of smoking than no change in smoking behavior<br>(OR: 18.22, 95% CI = 2.30–144.31).	Decrease or cessation of smoking in people with coronaphobia was found to be significantly more significant in the individuals who had severe anxiety than in those who increased their smoking<br>(OR:10.67, 95% CI = 1.17–97.19).
R. Cancello[11] Italy	18-30:14.5%<br>31-	Total: 490<br>Cigarette-only users: 105(21.4%)	Cigarette Consumption increase: 38%		

For peer review only - http://bmjopen.bmj.com/site/about/guidelines.xhtml

First Author, Setting Period	Age	Type of cigarette(N,%)	Subject 1* Smoking behavior	Subject 2* Smoking cessation	Subject 3* Psychological states
15th April- 4th May	60:65.1%<br>≥60: 20.4%				
Chopra[12]<br>India<br>15th August- 30th August	Mean(SD)<br>33.33(14.5)	No smoking: 939(94.4%)<br>Cigarette-only users: 56	Smoking consumption is significantly reduced (Mean=0.02, $P < 0.05$).		

*Subject 1 is a case in which a paper contains changes in smoking behavior before and during the COVID-19 pandemic. Subject 2 is a case in which a paper contains perceptions of smoking or cessation intention before and during the COVID-19 pandemic. Subject 3 is a case in which a paper contains information on changes in psychological states, such as depression, stress, and anxiety before and during the COVID-19 pandemic.

For peer review only

4. DISCUSSION

We examined the changes in smoking behavior, cessation intentions, and psychological states caused by the COVID-19 outbreak in smokers (cigarette-only users, e-cigarette-only users, and dual users). Regardless of the cigarette type, the amount of smoking either increased or decreased in some cases. However, compared to e-cigarette-only users, most cigarette-only users' intentions and attempts to cease smoking increased. In addition, regardless of the type of cigarette, smokers showed a negative psychological state due to COVID-19. It was found that attempts and intentions to cease smoking increased as dependence on cigarettes decreased. Additionally, social distancing strengthened, and awareness about COVID-19 increased.

The amount of smoking done by e-cigarette-only users increased with increased dependency. For dual users, those who use cigarettes and e-cigarettes, efforts to reduce product usage increased; however, there was a tendency to buy cigarettes in bulk, increase e-cigarette usage skills, and increase their use. This is thought to result from a tendency to recognize that using e-cigarettes is relatively less harmful to health than smoking cigarettes.[23] According to previous studies, many e-cigarette users recognize that e-cigarettes are relatively less harmful to health, their own and others, [23] because they contain far fewer harmful components [41] than cigarettes. However, it is not yet clear how e-cigarettes can help cigarette users quit smoking.[22] For this reason, e-cigarette users should consider the risks of using e-cigarettes as much as cigarettes.

Cigarette-only users' efforts to reduce product usage and quit smoking during the pandemic increased, especially when the average age group was high. In other words, the higher the age group, the higher the risk of COVID-19 infection and symptom severity. Therefore, it was found that the use of cigarettes decreased.[25] Efforts to reduce product usage by e-cigarette users, regardless of age, decreased. In particular, the higher the dependence of e-cigarette users, the higher were the worries related to COVID-19; however, efforts to reduce the amount of smoking were lower among users with low e-cigarette dependence. This could be because e-cigarette users think that using e-cigarettes is an attempt to quit smoking.[23] Therefore, e-cigarette users' intentions to quit smoking have not increased, even during the pandemic. A cohort analysis conducted in the US found that e-cigarette users did not quit smoking, compared to cigarette-only users, and more than half of e-cigarette users still used e-cigarettes a year later.[42] In another study, it was unclear whether e-cigarette users were more likely to quit smoking.[43] Since many studies are still being conducted on the cessation effect of e-cigarettes, it should be recognized that e-cigarette use could be harmful to health.

Regardless of the cigarette type, the higher the awareness of the risks associated with contracting COVID-19, the higher the level of social distancing since the start of COVID-19 and intent to quit smoking. In the case of cigarette-only users, coronaphobia increased smoking cessation intentions. Among them, those who reduced smoking felt more anxious than those who increased their amount of smoking. Smokers were more likely to quit after understanding the health risks of smoking due to the "vulnerability hypothesis," wherein smokers who care about their health would be inclined to quit smoking when they know it makes them vulnerable to certain diseases.[44, 45] This presents the same context as the results of this systematic review.

In contrast, previous studies have shown that smokers have higher levels of depression, anxiety,

For peer review only - http://bmjopen.bmj.com/site/about/guidelines.xhtml

stress, and increased smoking amounts. According to a study by Klemperer et al.,[46] 28.3% of patients reduced their tobacco use due to fear of infectious diseases, while 30.3% showed increased tobacco use and anxiety levels in both groups. However, in the literature surveyed in this study, it was found that the higher the level of anxiety, the higher the attempt to quit smoking. Therefore, further research exploring the relationship between mental health and smoking behaviors during the COVID-19 pandemic using standardized tools to measure mental health conditions, such as anxiety, is needed.

Increased awareness of COVID-19 risk has led to increased smoking cessation attempts, which can be considered a desirable outcome for physical health in the long run. However, for mental health, including anxiety, depression, and increased stress during the pandemic, governments' response to address the increasing trend of smoking should be to conduct campaigns for risk awareness to protect their citizens' mental health. Research on the association between cigarette smoking and COVID-19 increases; the WHO also suggests that smokers may be considered at high risk of contracting COVID-19. However, because of the lack of research on the relationship between the use of liquid e-cigarettes and COVID-19, the willingness or attempts to quit e-cigarette use have not increased compared with cigarette-only users or dual users. Therefore, this study was able to review only cross-sectional studies. As smokers have a high risk of contracting certain diseases, both during and after the COVID-19 pandemic, further studies on the risk of e-cigarettes and their impact on smoking cessation, especially regarding COVID-19 infection, should be conducted.

This study is the first to systematically review the literature regarding changes in smoking behaviors, cessation intentions, and psychological states of smokers, based on the study period, country, and age of participants during the COVID-19 pandemic. Since this study aimed to investigate people's smoking behavior, smoking cessation, and psychological state in the early stages of the COVID-19 pandemic in 2020, all studies used questionnaire tools. There were no articles that had conducted qualitative or interventional studies on this topic. Therefore, only cross-sectional studies were included. This study only systematically reviewed articles published between January and December 2020. According to the results of this study, as social distancing and lockdown were strengthened in the early stages of the pandemic, smoking behavior, smoking cessation intention, and psychological changes appeared. However, in 2021, as vaccinations began to occur worldwide, social distancing decreased, as did people's willingness to participate in social distancing and stay-at-home.[47] Accordingly, smoking behavior, smoking cessation, and psychological status in 2021 may differ from that in 2020. When reviewing articles published in 2021, it is necessary to examine the changes in smoking behavior, smoking cessation, and psychological state of smokers while considering vaccine variables.

However, it is meaningful that this study looked at smoking usage behavior during the COVID-19 pandemic based on cigarette type. This study could serve as primary data to estaablish effective strategies that focus on specific tobacco-product users by suggesting different smoking behaviors, smoking cessation intentions, and psychological changes based on cigarette type.

For peer review only - http://bmjopen.bmj.com/site/about/guidelines.xhtml

5. CONCLUSION

In this study, we examined changes in smoking behaviors, smoking cessation intentions, and psychological states of smokers during COVID-19 according to cigarette type. We conducted a systematic review of 13 papers to suggest future research and policy directions that the government may implement for COVID-19 and smokers. During the COVID-19 pandemic, cigarette-only users' consumption did not change significantly, but dual users and e-cigarette-only users with a high dependence on e-cigarettes showed increased consumption. Cigarette users' quit intentions, attempts, and cessation success rates increased, and even those with coronaphobia showed increased intent to quit smoking. On the other hand, users of e-cigarettes demonstrated fewer attempts to quit. As the COVID-19 pandemic continues, policies and campaigns should be implemented to increase the intent and attempt to stop smoking. Research and interventions on COVID-19, smoking, and e-cigarette use should also be conducted in the medium to long term.

CONTRIBUTIORS

HC designed the study, analyzed and interpreted the data, and wrote the manuscript. JH and EC contributed to the study design and interpretation of the results.

FUNDING

This study was supported by the National Research Foundation of Korea (NRF) grant, funded by the Ministry of Science and ICT (MSIT) (No. 2020R1C1C1012562).

COMPETING INTERESTS

None.

PATIENT CONSENT FOR PUBLICATION

Not required.

ETHICS APPROVAL

This systematic review was based on published data. As researchers did not access any information that could lead to the identification of an individual patient, no ethical issues were raised in this research. Therefore, obtaining ethical approval and consent from the participants was waived.

PROVENANCE AND PEER REVIEW

Not commissioned; externally peer reviewed.

DATA SHARING STATEMENT

The data set used and/or analyzed for this review will be available from the corresponding author upon reasonable request.

For peer review only

For peer review only - http://bmjopen.bmj.com/site/about/guidelines.xhtml

REFERENCES

1. Berlin I, Thomas D, Le Faou A-L, Cornuz J. COVID-19 and smoking. *Nicotine and Tobacco Research*. 2020;22(9):1650-1652.
2. Organization WH. COVID 19 Public Health Emergency of International Concern (PHEIC). Global research and innovation forum: towards a research roadmap. 2020;
3. Our World in Data. Explore the global data on confirmed COVID-19 cases. doi:https://ourworldindata.org/covid-cases Accessed December 31, 2020.
4. Our World in Data. Explore the global data on confirmed COVID-19 deaths. doi:https://ourworldindata.org/covid-deaths Accessed December 31, 2020.
5. Ni Y, Shi G, Qu J. Indoor PM2. 5, tobacco smoking and chronic lung diseases: A narrative review. *Environmental research*. 2020;181:108910.
6. Vardavas CI, Nikitara K. COVID-19 and smoking: A systematic review of the evidence. *Tobacco induced diseases*. 2020;18
7. Health UDo, Services H. The health consequences of smoking—50 years of progress: a report of the Surgeon General. Atlanta, GA: US Department of Health and Human Services, Centers for Disease ...; 2014.
8. Umnuaypornlert A, Kanchanasurakit S, Lucero-Prisno DEI, Saokaew S. Smoking and risk of negative outcomes among COVID-19 patients: A systematic review and meta-analysis. *Tobacco induced diseases*. 2021;19
9. Hwang JH, Lyes M, Sladewski K, et al. Electronic cigarette inhalation alters innate immunity and airway cytokines while increasing the virulence of colonizing bacteria. *Journal of molecular medicine*. 2016;94(6):667-679.
10. Kaur G, Lungarella G, Rahman I. SARS-CoV-2 COVID-19 susceptibility and lung inflammatory storm by smoking and vaping. *Journal of Inflammation*. 2020/06/10 2020;17(1):21. doi:10.1186/s12950-020-00250-8
11. Cancello R, Soranna D, Zambra G, Zambon A, Invitti C. Determinants of the lifestyle changes during COVID-19 pandemic in the residents of Northern Italy. *International journal of environmental research and public health*. 2020;17(17):6287.
12. Chopra S, Ranjan P, Singh V, et al. Impact of COVID-19 on lifestyle-related behaviours-a cross-sectional audit of responses from nine hundred and ninety-five participants from India. *Diabetes & Metabolic Syndrome: Clinical Research & Reviews*. 2020;14(6):2021-2030.
13. Tattan-Birch H, Perski O, Jackson S, Shahab L, West R, Brown J. COVID-19, smoking, vaping and quitting: a representative population survey in England. *Addiction*. 2020;
14. Caponnetto P, Inguscio L, Saitta C, Maglia M, Benfatto F, Polosa R. Smoking behavior and psychological dynamics during COVID-19 social distancing and stay-at-home policies: A survey. *Health psychology research*. 2020;8(1)

For peer review only - http://bmjopen.bmj.com/site/about/guidelines.xhtml

15. Wood W, Tam L, Witt MG. Changing circumstances, disrupting habits. *Journal of personality and social psychology*. 2005;88(6):918.

16. Stanton R, To QG, Khalesi S, et al. Depression, Anxiety and Stress during COVID-19: Associations with Changes in Physical Activity, Sleep, Tobacco and Alcohol Use in Australian Adults. *Int J Environ Res Public Health*. Jun 7 2020;17(11)doi:10.3390/ijerph17114065

17. Fancourt D, Bu F, Mak HW, Steptoe A. COVID-19 social study. *Results release*. 2020;15

18. Volkow ND. Collision of the COVID-19 and addiction epidemics. American College of Physicians; 2020.

19. Parrott AC. Does cigarette smoking cause stress? *American Psychologist*. 1999;54(10):817.

20. García-Álvarez L, Fuente-Tomás L, Sáiz PA, García-Portilla MP, Bobes J. Will changes in alcohol and tobacco use be seen during the COVID-19 lockdown? *Adicciones*. Apr 1 2020;32(2):85-89. ¿Se observarán cambios en el consumo de alcohol y tabaco durante el confinamiento por COVID-19? doi:10.20882/adicciones.1546

21. Adriaens K, Van Gucht D, Baeyens F. Differences between dual users and switchers center around vaping behavior and its experiences rather than beliefs and attitudes. *International journal of environmental research and public health*. 2018;15(1):12.

22. Grana RA, Popova L, Ling PM. A longitudinal analysis of electronic cigarette use and smoking cessation. *JAMA internal medicine*. 2014;174(5):812-813.

23. Etter JF, Bullen C. Electronic cigarette: users profile, utilization, satisfaction and perceived efficacy. *Addiction*. 2011;106(11):2017-2028.

24. Chadi N, Schroeder R, Jensen JW, Levy S. Association between electronic cigarette use and marijuana use among adolescents and young adults: a systematic review and meta-analysis. *JAMA pediatrics*. 2019;173(10):e192574-e192574.

25. Chen Y, Klein SL, Garibaldi BT, et al. Aging in COVID-19: Vulnerability, immunity and intervention. *Ageing research reviews*. 2021;65:101205.

26. Kim SY, Park JE, Lee YJ, et al. Testing a tool for assessing the risk of bias for nonrandomized studies showed moderate reliability and promising validity. *Journal of clinical epidemiology*. 2013;66(4):408-414.

27. Wohlin C. Guidelines for snowballing in systematic literature studies and a replication in software engineering. 2014:1-10.

28. Greenhalgh T, Peacock R. Effectiveness and efficiency of search methods in systematic reviews of complex evidence: audit of primary sources. *Bmj*. 2005;331(7524):1064-1065.

29. Soule EK, Mayne S, Snipes W, Guy MC, Breland A, Fagan P. Impacts of COVID-19 on electronic cigarette purchasing, use and related behaviors. *International Journal of Environmental Research and Public Health*. 2020;17(18):6762.

30. Caponnetto P, Inguscio L, Saitta C, Maglia M, Benfatto F, Polosa R. Smoking behavior and psychological dynamics during COVID-19 social distancing and stay-at-home policies: A survey.

For peer review only

For peer review only - http://bmjopen.bmj.com/site/about/guidelines.xhtml

Health Psychol Res. May 27 2020;8(1):9124. doi:10.4081/hpr.2020.9124
31. Ren Y, Qian W, Li Z, et al. Public mental health under the long-term influence of COVID-19 in China: Geographical and temporal distribution. *Journal of Affective Disorders*. 2020;277:893-900.
32. Ozcelik N, Yilmaz Kara B. Effect of coronaphobia on smoking habits. *Journal of addictive diseases*. 2020:1-9.
33. Chertok IRA. Perceived risk of infection and smoking behavior change during COVID-19 in Ohio. *Public Health Nursing*. 2020;37(6):854-862.
34. Jackson SE, Garnett C, Shahab L, Oldham M, Brown J. Association of the Covid-19 lockdown with smoking, drinking, and attempts to quit in England: an analysis of 2019-2020 data. *medRxiv*. 2020;
35. Kayhan Tetik B, Gedik Tekinemre I, Taş S. The Effect of the COVID-19 Pandemic on Smoking Cessation Success. *J Community Health*. Jul 8 2020:1-5. doi:10.1007/s10900-020-00880-2
36. Kowitt SD, Cornacchione Ross J, Jarman KL, et al. Tobacco quit intentions and behaviors among cigar smokers in the United States in response to COVID-19. *International journal of environmental research and public health*. 2020;17(15):5368.
37. Jackson SE, Brown J, Shahab L, Steptoe A, Fancourt D. COVID-19, smoking and inequalities: a study of 53 002 adults in the UK. *Tobacco control*. 2020;
38. Stanton R, To QG, Khalesi S, et al. Depression, anxiety and stress during COVID-19: associations with changes in physical activity, sleep, tobacco and alcohol use in Australian adults. *International journal of environmental research and public health*. 2020;17(11):4065.
39. Jackson SE, Garnett C, Shahab L, Oldham M, Brown J. Association of the COVID-19 lockdown with smoking, drinking and attempts to quit in England: an analysis of 2019–20 data. *Addiction*. 2021;116(5):1233-1244.
40. Tetik BK, Tekinemre IG, Taş S. The effect of the COVID-19 pandemic on smoking cessation success. *Journal of Community Health*. 2020:1-5.
41. Goniewicz ML, Knysak J, Gawron M, et al. Levels of selected carcinogens and toxicants in vapour from electronic cigarettes. *Tobacco control*. 2014;23(2):133-139.
42. Pierce JP, Benmarhnia T, Chen R, et al. Role of e-cigarettes and pharmacotherapy during attempts to quit cigarette smoking: The PATH Study 2013-16. *PloS one*. 2020;15(9):e0237938.
43. Pasquereau A, Guignard R, Andler R, Nguyen-Thanh V. Electronic cigarettes, quit attempts and smoking cessation: a 6-month follow-up. *Addiction*. 2017;112(9):1620-1628.
44. Borrelli B, Hayes RB, Dunsiger S, Fava JL. Risk perception and smoking behavior in medically ill smokers: a prospective study. *Addiction*. 2010;105(6):1100-1108.
45. Weinstein ND. The precaution adoption process. *Health psychology*. 1988;7(4):355.
46. Klemperer EM, West JC, Peasley-Miklus C, Villanti AC. Change in Tobacco and Electronic Cigarette Use and Motivation to Quit in Response to COVID-19. *Nicotine Tob Res*. Aug 24

For peer review only - http://bmjopen.bmj.com/site/about/guidelines.xhtml

2020;22(9):1662-1663. doi:10.1093/ntr/ntaa072

47. Andersson O, Campos-Mercade P, Meier AN, Wengström E. Anticipation of COVID-19 vaccines reduces willingness to socially distance. *Journal of health economics*. 2021;80:102530.

For peer review only

For peer review only - http://bmjopen.bmj.com/site/about/guidelines.xhtml

Figure caption/ legend

Figure 1 Flow chart diagram

Legend : The flowchart shows the article selection process, the criteria for exclusion of articles, and the number of articles excluded.

Figure 2 Assessment of the risk of bias in identified studies using the RoBANS questionnaire

Legend : Author judgment for each RoBANS domain for the included articles. Green indicates a low risk of bias, yellow indicates unclear risk, and red indicates a high risk of bias. The six domains are "comparability of groups, participant selection, confounding variables, measurement of exposure, blinding of the outcome, incomplete outcome data, selective outcome reporting."

Figure 3 Assessment summary of the risk of bias in identified studies using the RoBANS questionnaire

Legend : Author judgment for each RoBANS domain for the included articles. Green circles indicate low risk of bias, yellow circles indicate unclear risk, and red circles indicate high risk of bias. The study numbers indicate the order of the articles presented in Table 1.

For peer review only - http://bmjopen.bmj.com/site/about/guidelines.xhtml

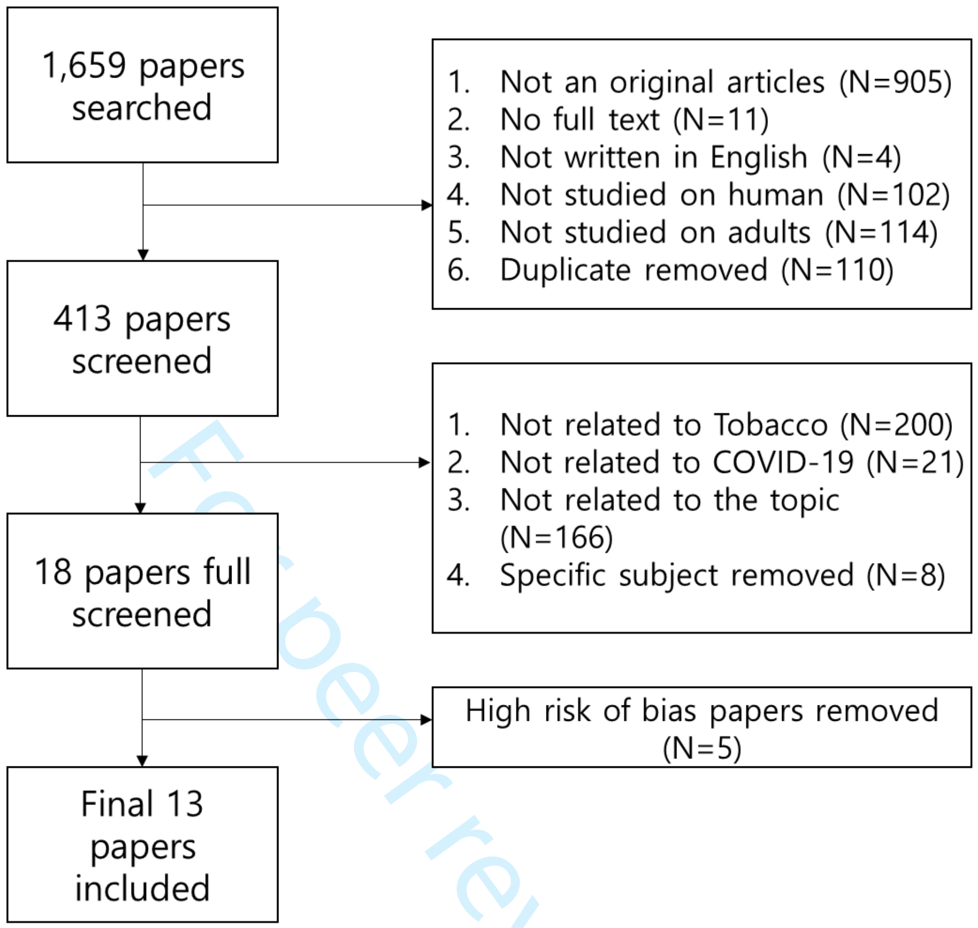
1,659 papers searched
1. Not an original articles (N=905)
2. No full text (N=11)
3. Not written in English (N=4)
4. Not studied on human (N=102)
5. Not studied on adults (N=114)
6. Duplicate removed (N=110)
413 papers screened
1. Not related to Tobacco (N=200)
2. Not related to COVID-19 (N=21)
3. Not related to the topic (N=166)
4. Specific subject removed (N=8)
18 papers full screened
High risk of bias papers removed (N=5)
Final 13 papers included

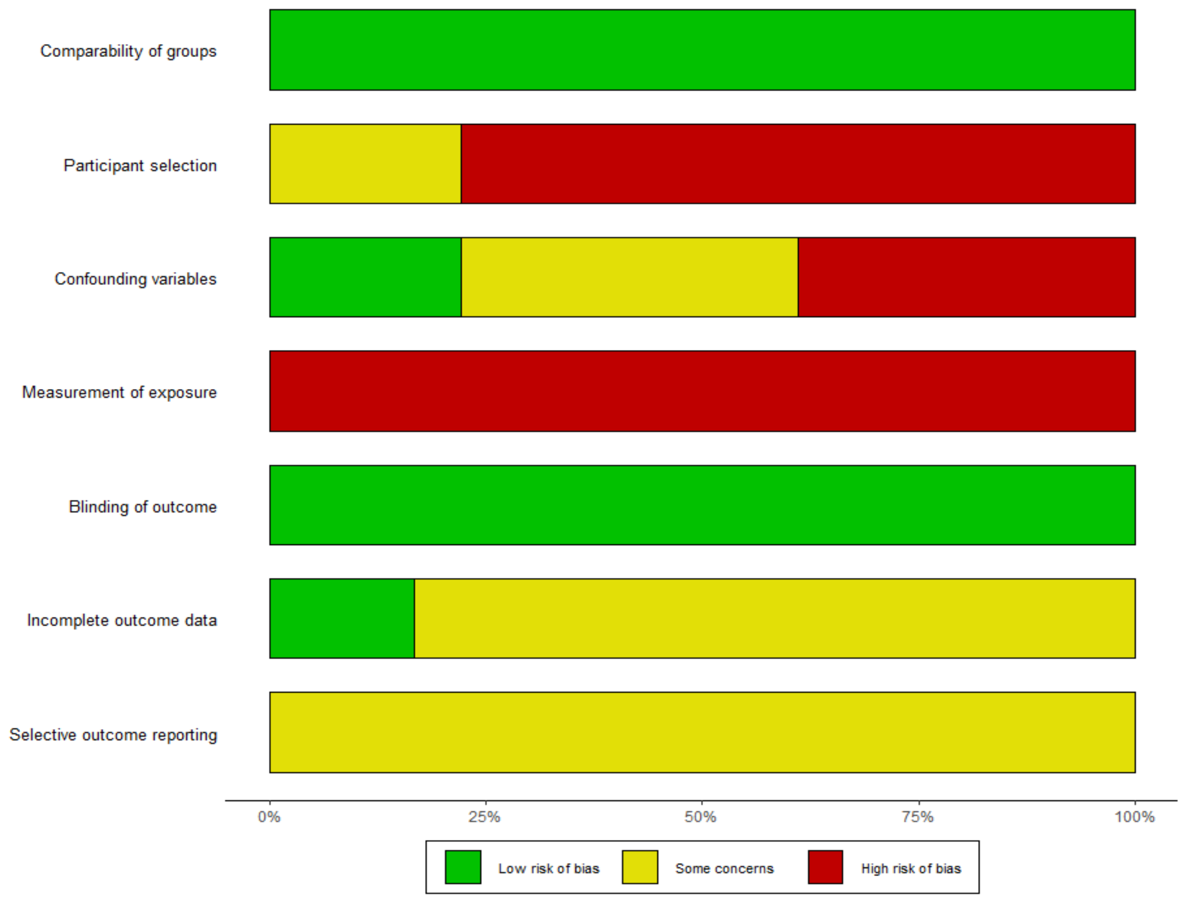
Comparability of groups
Participant selection
Confounding variables
Measurement of exposure
Blinding of outcome
Incomplete outcome data
Selective outcome reporting
0%
25%
50%
75%
100%
Low risk of bias
Some concerns
High risk of bias

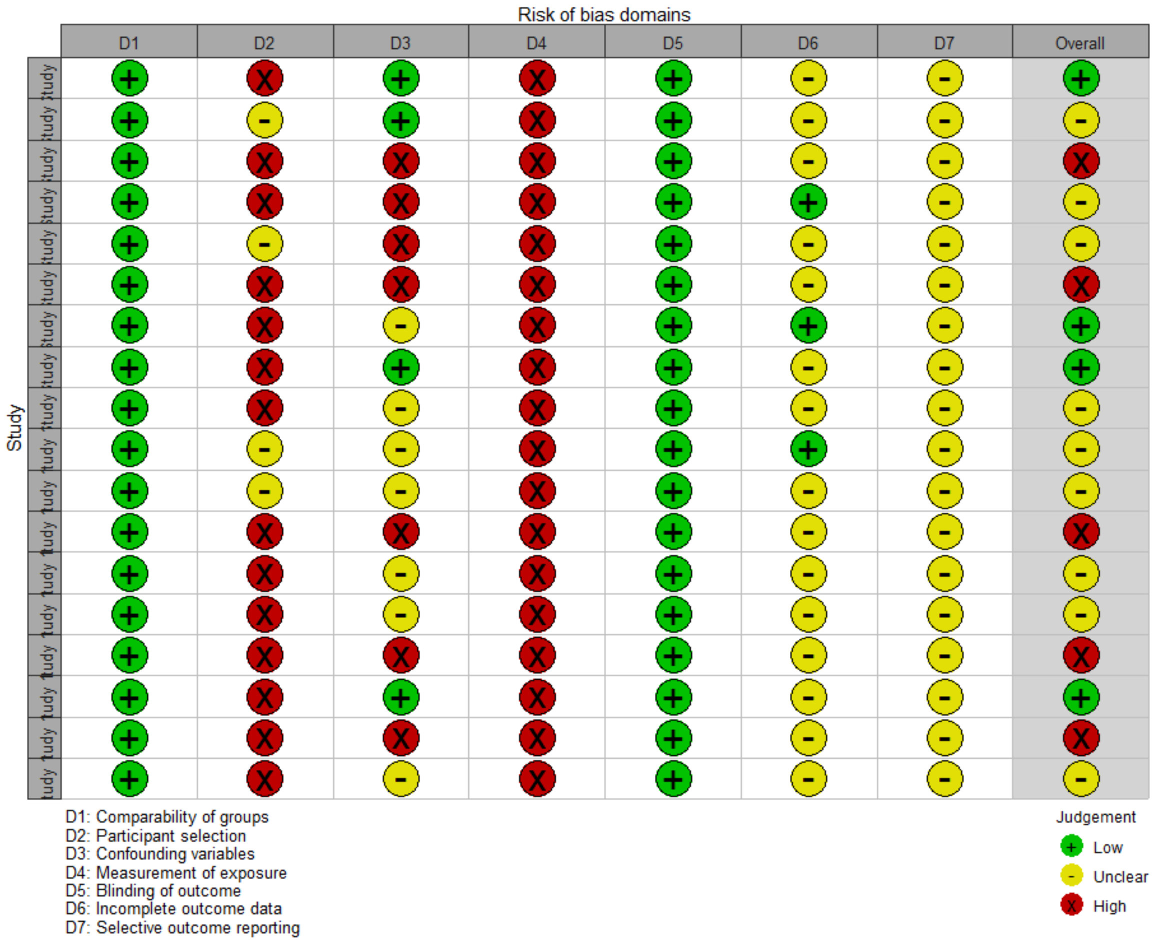

For peer review only

Supplemantary

Online Appendix：Search terms

Table 1 Pubmed search terms

Electronic Nicotine Delivery Systems
(((((((((((((((((((((((((((((((((((((((Electronic Nicotine Delivery System[Title/Abstract]) OR (Electronic Cigarettes[Title/Abstract])) OR (E-Cigs[Title/Abstract])) OR (E Cigs[Title/Abstract])) OR (E-Cig[Title/Abstract])) OR (E Cig[Title/Abstract])) OR (E-Cigarettes[Title/Abstract])) OR (E Cigarettes[Title/Abstract])) OR (E-Cigarette[Title/Abstract])) OR (E Cigarette[Title/Abstract])) OR (Electronic Cigarette[Title/Abstract])) OR (Cigarette, Electronic[Title/Abstract])) OR (Cigarettes, Electronic[Title/Abstract])) OR ("Electronic Nicotine Delivery Systems"[Mesh])) OR ("Vaping"[Mesh])) OR (E-Cig Use[Title/Abstract])) OR (E Cig Use[Title/Abstract])) OR (E-Cig Uses[Title/Abstract])) OR (Use, E-Cig[Title/Abstract])) OR (ECig Use[Title/Abstract])) OR (ECig Uses[Title/Abstract])) OR (Use, ECig[Title/Abstract])) OR (Vape[Title/Abstract])) OR (Vapes[Title/Abstract])) OR (E-Cigarette Use[Title/Abstract])) OR (E Cigarette Use[Title/Abstract])) OR (E-Cigarette Uses[Title/Abstract])) OR (Use, E-Cigarette[Title/Abstract])) OR (Nicotine Vaping[Title/Abstract])) OR (Nicotine Vapings[Title/Abstract])) OR (Vaping, Nicotine[Title/Abstract])) OR (Vapings, Nicotine[Title/Abstract])) OR (Ecigarette Use[Title/Abstract])) OR (Ecigarette Uses[Title/Abstract])) OR (Use, Ecigarette[Title/Abstract])) OR (Uses, Ecigarette[Title/Abstract])) OR (Electronic Cigarette Use[Title/Abstract])) OR (Cigarette Use, Electronic[Title/Abstract])) OR (Electronic Cigarette Uses[Title/Abstract])) OR (Use, Electronic Cigarette[Title/Abstract])
COVID-19
(((((((((((((((((((((((((((((((((((COVID 19[Title/Abstract]) OR (COVID-19 Virus Disease[Title/Abstract])) OR (COVID 19 Virus Disease[Title/Abstract])) OR (COVID-19 Virus Diseases[Title/Abstract])) OR (Disease, COVID-19 Virus[Title/Abstract])) OR (Virus Disease, COVID-19[Title/Abstract])) OR (COVID-19 Virus Infection[Title/Abstract])) OR (COVID 19 Virus Infection[Title/Abstract])) OR (COVID-19 Virus Infections[Title/Abstract])) OR (Infection, COVID-19 Virus[Title/Abstract])) OR (Virus Infection, COVID-19[Title/Abstract])) OR (2019-nCoV Infection[Title/Abstract])) OR (2019 nCoV Infection[Title/Abstract])) OR (2019-nCoV Infections[Title/Abstract])) OR (Infection, 2019-nCoV[Title/Abstract])) OR (Coronavirus Disease-19[Title/Abstract])) OR (Coronavirus Disease 19[Title/Abstract])) OR (2019 Novel Coronavirus Disease[Title/Abstract])) OR (2019 Novel Coronavirus Infection[Title/Abstract])) OR (2019-nCoV Disease[Title/Abstract])) OR (2019 nCoV Disease[Title/Abstract])) OR (2019-nCoV

Diseases[Title/Abstract])) OR (Disease, 2019-nCoV[Title/Abstract])) OR (COVID19[Title/Abstract])) OR (Coronavirus Disease 2019[Title/Abstract])) OR (Disease 2019, Coronavirus[Title/Abstract])) OR (SARS Coronavirus 2 Infection[Title/Abstract])) OR (Infection, SARS-CoV-2[Title/Abstract])) OR (SARS-CoV-2 Infection[Title/Abstract])) OR (SARS CoV 2 Infection[Title/Abstract])) OR (SARS-CoV-2 Infections[Title/Abstract])) OR (COVID-19 Pandemic[Title/Abstract])) OR (COVID 19 Pandemic[Title/Abstract])) OR (COVID-19 Pandemics[Title/Abstract])) OR (Pandemic, COVID-19[Title/Abstract])) OR ("COVID-19"[Mesh])
Tobacco, Tobacco Products, Tobacco Use
(((((Tobacco Uses[Title/Abstract]) OR (Tobacco Consumption[Title/Abstract])) OR (Consumption, Tobacco[Title/Abstract])) OR ("Tobacco Use"[Mesh])) OR ((((((((((((((((Tobaccos[Title/Abstract]) OR (Nicotiana[Title/Abstract])) OR (Nicotianas[Title/Abstract])) OR (Nicotiana tabacum[Title/Abstract])) OR (Nicotiana tabacums[Title/Abstract])) OR (tabacum, Nicotiana[Title/Abstract])) OR ("Tobacco"[Mesh])) OR ("Smoking"[Mesh])) OR (Smoking Behaviors[Title/Abstract])) OR (Behavior, Smoking[Title/Abstract])) OR (Behaviors, Smoking[Title/Abstract])) OR (Smoking Behavior[Title/Abstract])) OR (Smoking Habit[Title/Abstract])) OR (Habit, Smoking[Title/Abstract])) OR (Habits, Smoking[Title/Abstract])) OR (Smoking Habits[Title/Abstract]))) OR ((((((((((((((((((((((((((Product, Tobacco[Title/Abstract]) OR (Products, Tobacco[Title/Abstract])) OR (Tobacco Product[Title/Abstract])) OR (Cigarillos[Title/Abstract])) OR (Cigarillo[Title/Abstract])) OR (Pipe Tobacco[Title/Abstract])) OR (Pipe Tobaccos[Title/Abstract])) OR (Tobacco, Pipe[Title/Abstract])) OR (Cigars[Title/Abstract])) OR (Tobaccos, Pipe[Title/Abstract])) OR (Cigar[Title/Abstract])) OR (Kreteks[Title/Abstract])) OR (Kretek[Title/Abstract])) OR (Kreteks Tobacco[Title/Abstract])) OR (Kreteks Tobaccos[Title/Abstract])) OR (Tobacco, Kreteks[Title/Abstract])) OR (Tobaccos, Kreteks[Title/Abstract])) OR (Bidis[Title/Abstract])) OR (Bidi[Title/Abstract])) OR (Bidis Tobacco[Title/Abstract])) OR (Bidis Tobaccos[Title/Abstract])) OR (Tobacco, Bidis[Title/Abstract])) OR (Cigarettes[Title/Abstract])) OR (Cigarette[Title/Abstract])) OR ("Tobacco Products"[Mesh]))
Psychology
(("Psychology"[Mesh]) OR ("Psychology/diagnosis"[Mesh] OR "Psychology/psychology"[Mesh])) OR ((((((((((((((Side Effects, Psychological[Title/Abstract]) OR (Psychological Side Effect[Title/Abstract])) OR (Side Effect, Psychological[Title/Abstract])) OR (Psychological Side Effects[Title/Abstract])) OR (Psychosocial Factors[Title/Abstract])) OR (Factor, Psychosocial[Title/Abstract])) OR (Factors, Psychosocial[Title/Abstract])) OR (Psychosocial Factor[Title/Abstract])) OR (Psychological Factors[Title/Abstract])) OR

For peer review only

(Factor, Psychological[Title/Abstract])) OR (Psychological Factor[Title/Abstract])) OR (Factors, Psychological[Title/Abstract])) OR (Psychologist[Title/Abstract])) OR (Psychologists[Title/Abstract]))
Anxiety
(((((((Angst[Title/Abstract]) OR (Nervousness[Title/Abstract])) OR (Hypervigilance[Title/Abstract])) OR (Anxiousness[Title/Abstract])) OR (Social Anxiety[Title/Abstract])) OR (Anxieties, Social[Title/Abstract])) OR (Anxiety, Social[Title/Abstract])) OR (Social Anxieties[Title/Abstract])) OR (("Anxiety"[Mesh]) OR ("Anxiety/analysis"[Mesh] OR "Anxiety/diagnosis"[Mesh] OR "Anxiety/psychology"[Mesh]))
Stress, Psychological
((((((((((((Psychological Stresses[Title/Abstract]) OR (Stresses, Psychological[Title/Abstract])) OR (Life Stress[Title/Abstract])) OR (Life Stresses[Title/Abstract])) OR (Stress, Life[Title/Abstract])) OR (Stresses, Life[Title/Abstract])) OR (Stress, Psychologic[Title/Abstract])) OR (Psychologic Stress[Title/Abstract])) OR (Stressor, Psychological[Title/Abstract])) OR (Psychological Stressor[Title/Abstract])) OR (Psychological Stressors[Title/Abstract])) OR (Stressors, Psychological[Title/Abstract])) OR (Psychological Stress[Title/Abstract])) OR (("Stress, Psychological"[Mesh]) OR ("Stress, Psychological/analysis"[Mesh] OR "Stress, Psychological/diagnosis"[Mesh] OR "Stress, Psychological/psychology"[Mesh]))
Depression
(("Depression"[Mesh]) OR ("Depression/analysis"[Mesh] OR "Depression/diagnosis"[Mesh] OR "Depression/psychology"[Mesh])) OR ((((((((Depressions[Title/Abstract]) OR (Depressive Symptoms[Title/Abstract])) OR (Depressive Symptom[Title/Abstract])) OR (Symptom, Depressive[Title/Abstract])) OR (Symptoms, Depressive[Title/Abstract])) OR (Emotional Depression[Title/Abstract])) OR (Depression, Emotional[Title/Abstract])) OR (Depressions, Emotional[Title/Abstract])) OR (Emotional Depressions[Title/Abstract]))

Table 2 Science Direct search terms

Query
COVID AND (e-cigarette OR "electronic cigarette" OR "electronic nicotine delivery" OR "vaping" OR "heat not burn" OR "heated tobacco product")
COVID AND (tobaccos OR cigarette OR kretek OR bidis OR "pipe tobacco" OR cigarillos)

For peer review only
